# Impact of clostridial glucosylating toxins on the proteome of colonic cells determined by isotope-coded protein labeling and LC-MALDI

**DOI:** 10.1186/1477-5956-9-48

**Published:** 2011-08-17

**Authors:** Nelli Jochim, Ralf Gerhard, Ingo Just, Andreas Pich

**Affiliations:** 1 Hannover Medical School, Institute of Toxicology, Carl-Neuberg-Str. 1, D-30625 Hannover, Germany

**Keywords:** *C. difficile*-associated diarrhea, colonic cells, ICPL™, relative quantification, Toxin A

## Abstract

**Background:**

The anaerobe *Clostridium difficile *produces two major virulence factors toxin A and B that inactivate Rho proteins by glucosylation of a pivotal threonine residue. Purified toxins induce reorganization of the cytoskeleton and cell death in colonic cells. Whether all toxin effects on target cells depend on catalytic glucosyltransferase activity is unclear at present. Thus, we conducted a proteome approach to compare the protein profile of target cells treated either with wild type toxin A (rTcdA wt) or with a catalytically inactive mutant toxin A (mutant rTcdA). Relative protein quantification was feasible using isotope-coded protein labeling techniques (ICPL) and mass spectrometry (LC-MALDI).

**Results:**

Altogether we found a significant differential expression of thirty proteins after treatment with rTcdA wt or mutant rTcdA. Mutant rTcdA caused up-regulation of seven proteins and sixteen proteins were responsive to rTcdA wt after 5 h. Long-term effect of rTcdA wt on protein expression was the down-regulation of eleven proteins. Up- or down-regulation of several proteins was verified by western blot analysis confirming the MS results.

**Conclusion:**

Our results indicate incubation time-dependent effects of the clostridial glucosylating toxin A on colonic cells. The rTcdA wt impact more cellular functions than actin cytoskeleton reorganization and apoptosis. Furthermore, these data give insight into glucosyltransferase independent effects of clostridial glucosylating toxins on target cells after short incubation time. Additionally, our data reveal pro-inflammatory and proliferative effects of mutant rTcdA after short-term incubation.

## Background

*Clostridium difficile *is a spore-forming anaerobe, which produces two major virulence factors, Toxin A (TcdA) and Toxin B (TcdB) [1]. TcdA and TcdB are the causative agents of the *C. difficile*-associated diarrhea (CDAD), a nosocomial infection with increasing morbidity and mortality due to the emergence of hypervirulent strains [2,3]. Treatment with broad-spectrum antibiotics contributes to colonization of the colon with toxin producing *C. difficile*. The CDAD is characterized by a loss of mucosal barrier function, secretory diarrhea and colonic inflammation [4].

TcdA and TcdB are homologous single chain toxins and are composed of an N-terminally located glucosyltransferase domain and a large delivery domain. The latter comprises a receptor binding domain, a transmembrane domain and a cysteine protease domain [4]. The glucosyltranferase domain includes an aspartate-any amino acid-aspartate motif (D-X-D) and a conserved tryptophan that participates in the coordination of a manganese ions and the sugar donor UDP-glucose, which are essential for enzymatic glucosyltransferase activity [1]. The mutation of the D-X-D motif to N-X-N decreases glucosyltransferase activity by factor of 6,900 compared to that of wild type recombinant TcdA, so that the mutant toxin is in fact catalytically inactive [5]. The toxins monoglucosylate the Rho GTPases Rho, Rac, and Cdc42 and are therefore assigned as clostridial glucosylating toxins [6]. Rho proteins regulate cell morphology, gene transcription, and cell proliferation [7]. The inactivation of Rho, Rac and Cdc42 causes actin depolymerization resulting in cell rounding (cytopathic effect) and eventually leads to cell death (cytotoxic effect).

Several studies reported glucosyltransferase-independent effects of TcdA on colonic cells resulting in activation of mitogen-activated protein kinases, generation of reactive oxygen species and stimulation of protein kinases PKC α and β [8-10]. The apoptotic effects have been assumed to be triggered independently of the glucosyltransferase activity.

However, the studies from Gerhard *et al*. show a dependence on active TcdA leading to glucosylation of Rho GTPases for induction of apoptosis [11]. Thus, it is still unclear, how TcdA renders cells apoptotic. To further provide insights into these effects we investigated the changes in protein expression of epithelial colorectal adenocarcinoma cells (Caco-2) which are targets of *Clostridium difficile *toxins. The investigated cell line is much more susceptible to TcdA than to TcdB particular if the toxin is added from the apical side as obvious in cell culture plates [12].

To compare different cellular response to wild type TcdA (rTcdA wt) and enzyme deficient mutant TcdA (mutant rTcdA) the cytosolic fractions from Caco-2 cells treated with rTcdA wt or mutant rTcdA were analyzed applying isotope-coded protein labeling (ICPL™). ICPL is a useful and efficient approach for quantitative proteomics based on isotope tagging at free lysine residues and the N-terminus of intact proteins [13,14]. The complexity of cellular extracts was reduced by means of one-dimensional SDS-PAGE and reversed phase chromatography. Protein identification and quantification was performed by high resolution mass spectrometry (MALDI-TOF/TOF-MS) and data base analysis. Our results indicate biphasic activity of rTcdA wt (short and long term effects), and evidence is provided that the catalytic inactive toxin modifies the protein profile of colonic cells.

## Results

### Proteomic analysis of Caco-2 cells

**Figure 1:**
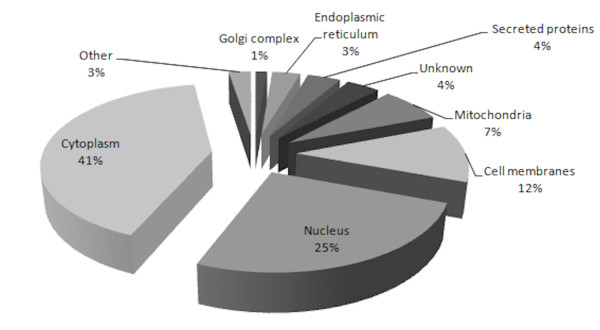
**Analysis of the Caco-2 proteome**.Caco-2 cells were harvested after reaching confluence, lysed and proteins were isolated. LC-MALDI-TOF/TOF analysis was performed after in-gel digestion of 200 μg of proteins from whole cell lysate. More than 2,000 proteins were identified and can be ordered to different cell compartments. The percentage of identified proteins for ER, secreted or unknown localization is given. The proteins of microsomes, cell junction, cell projection, endosomes, peroxisomes, lysosomes and centrosomes are summarized and named as other.

To study the proteome of colonic cells, a large scale proteome analysis was performed with Caco-2 cells grown under standard conditions. Protein extracts were separated by one-dimensional SDS-PAGE and subsequently proteins from different gel slices were digested and the resulting peptides were analyzed using an LC-MALDI approach. Overall, 2,042 proteins were identified by at least 2 peptides (Additional file 1). Most of the identified proteins could be assigned to the cytoplasm (844) or the nucleus (533) (Figure 1). 231 proteins (11%) of the identified proteins are known to appear in the membrane fraction of cells. Thus, with this technique a comprehensive amount of hydrophobic proteins could be identified including integrins, cadherins, small GTPases and various other proteins. A few of the identified proteins (49) are known to distribute in microsomes, cell membrane projection, endosomes, peroxysomes, lysosomes, centrosomes, and cell junctions. About 144 proteins (7%) are located at the mitochondria, 73 proteins (4%) are known to be secreted, 61 proteins (3%) belong to the endoplasmic reticulum, and 29 proteins (2%) are located at the trans-golgi network in intact cells. The localization of 78 proteins (4%) is unclear. The function of the identified proteins contributed to all dominant cellular activities such as growth, proliferation, motility, adhesion, protein transport, signal transduction, metabolism, cell cycle control, transcription, and translation. Some of the identified proteins, e.g. IgGF-binding protein, Cytokeratin-18, Src substrate cortactin are typically overexpressed in colorectal adenocarcinoma cells [15].

### Treatment of Caco-2 cells with rTcdA wt and mutant rTcdA

**Figure 2:**
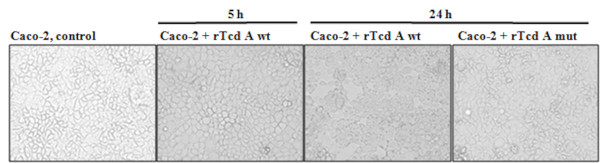
**Cytopathic effect of toxin A on Caco-2 cells**. Caco-2 cells were treated with 500 ng/ml recombinant toxin A wild type or with mutant recombinant toxin A in DMEM medium. Caco-2 cells were document before (0 h) and after 5 h or 24 h of toxin A treatment.

**Figure 3:**
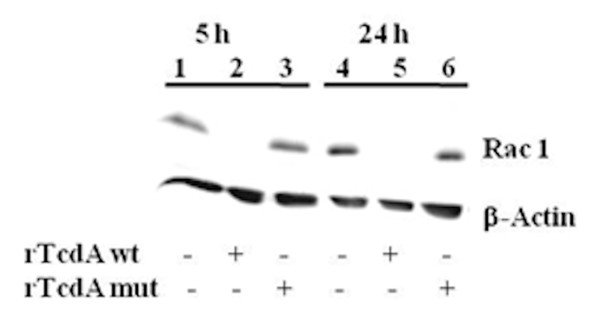
**rTcdA glucosylates Rac-1 in Caco-2 cells**. The cytopathic effect was shown by a Rac-1 glucosylation sensitive western blot analysis. The used antibody did not bind to glucosylated Rac-1. 1 and 4: control cells; 2 and 5: rTcdA wt treated cells; 3 and 6: mutant rTcdA treated cells.

Different toxin concentrations were tested to ensure that Caco-2 cells responded strongly to the clostridial toxins but remain viable. Optimal conditions were achieved when cells near confluence were treated with 0.5 μg/ml of rTcdA wt or mutant rTcdA for 5 h and 24 h (Figure 2). Cells treated with rTcdA wt started to round up after 2 h and after 5 h 70% and after 24 h nearly all Caco-2 cells rounded up as a result of the cytopathic activity of rTcdA wt. In contrast, control cells or cells treated with mutant rTcdA did not show any cell rounding and had a similar morphology. To confirm toxin activity, western blot analysis of cell lysates was performed using a Rac1 specific antibody that only recognizes non-glucosylated Rac1. The intracellular activity of rTcdA wt resulted in a decreasing concentration of nonglucosylated Rac1 visualized by intensity of the Rac1 band (Figure 3). In control cells and cells treated with mutant rTcdA only non-glucosylated Rac1 was present, whereas Rac1 from the cells treated with rTcdA wild type was not detected because it was completely glucosylated by the toxin.

### Quantitative proteomic analysis of Caco-2 cells treated with rTcdA wt and mutant rTcdA, respectively, for 5 h

To estimate the variation of the ICPL technique equal amounts of proteins from control cells were labeled with the duplex reagent in a 1:1 ratio. Mass spectrometric analysis was performed for three technical replicates (data not shown). The protein load was normalized by comparing the intensities of all analyzed peptides leading to normalization factors of 1.2 at maximum. The variability of this quantification method was determined for all labeled proteins to be between 1.4 and 0.7, the mean standard deviation was 0.22. Therefore, proteins with regulation factors above 1.4 were assigned as up-regulated and below 0.7 as down-regulated.

Proteins of the cytosolic fraction of control cells were obtained and two biological replicates were prepared and labeled. Cells treated for 5 h with rTcdA wt, and mutant rTcdA were labeled with heavy and medium ICPL-reagent. Untreated control cells were labeled with the light ICPL version. Subsequently, proteins were combined and analyzed. Overall, 152 different proteins were identified that had been quantified by at least 2 labeled peptides. It was carefully checked whether for ICPL-labeled peptides corresponding unlabeled peptides were present. No unlabeled peptide was detected if a labeled one was present. This analysis revealed the identification of 327 proteins by at least two unique peptides from the cytoplasm of Caco-2 cells and quantification of 278 proteins by at least one unique peptide.

**Figure 4:**
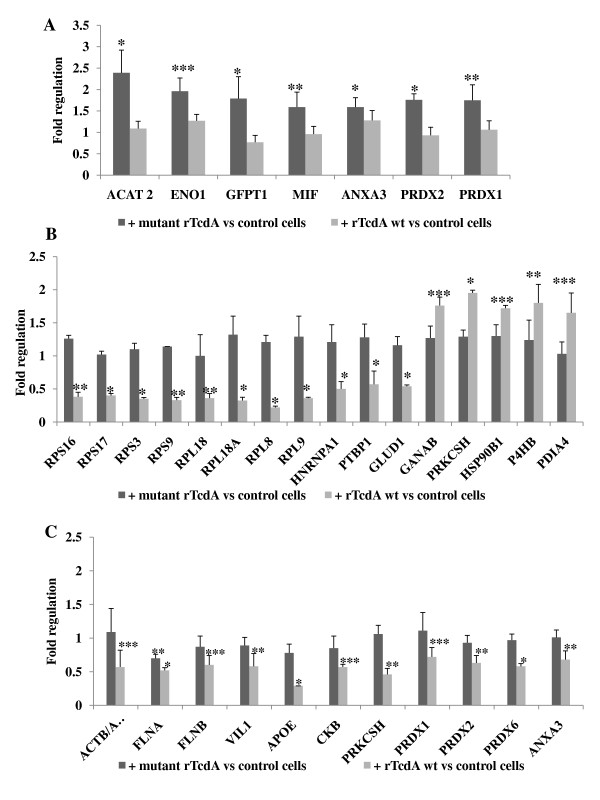
**Regulated proteins in Caco-2 cells after treatment with rTcdA wt or mutant rTcdA**. The regulation factors were calculated from all peak area of peptides identified for each protein and normalized to correct protein load. Statistical evaluation was performed using GraphPad Prism 5 (GraphPad Software, Inc.). *Bars *represent mean ± SD; *, p < 0.05, **, p < 0.001, ***, p > 0.0001 *versus *control cells. For each protein the corresponding regulation factor of mutant rTcdA or rTcdA wt approach is shown. **A) **After 5 h of treatment with mutant rTcdA; **B) **After 5 h of treatment with rTcdA wt; **C) **After 24 h of treatment with rTcdA wt or with mutant rTcdA.

False discovery rates for two replicates were 0.7% and 0.74% with p < 0.05. Based on normalization seven proteins in Caco-2 cells were up-regulated in response to treatment with mutant rTcdA (Table 1, Figure 4A, Additional file 2). The rTcdA wt treatment induced up-regulation of five proteins and down-regulation of eleven proteins in Caco-2 cells (Table 1, Figure 4B, Additional file 2). However, different proteins were responsive to rTcdA wt or mutant rTcdA treatment. Slight differences were observed in retention time for the deuteriumcontaining ICPL reagents. To overcome this problem the retention time tolerance was set to 4-times fractionation size in the WARP-LC method.

**Table 1:** Regulated proteins in Caco-2 cells after treatment with rTcdA wt or mutant rTcdA using ICPL-LC-MS-technique

	after treatment with mutant rTcdA, 5 h						
**Nr**.	**Protein name**	**Accession number**	**Gene name**	**Number of peptides for quantitation**	**regulation factor^1^**	**SD^2^**	**T-test^3^**

1	Acetyl-CoA acetyltransferase, cytosolic variant	Q9BWD1	ACAT 2	8	2.39	0.53	0.047
2	Alpha-enolase	P06733	ENO1	3	1.96	0.31	<0.0001
3	Annexin A3	P12429	ANXA3	5	1.59	0.22	0.015
4	Glucosamine--fructose-6-phosphate aminotransferase	Q06210	GFPT1	4	1.79	0.51	0.005
5	Macrophage migration inhibitory factor	P14174	MIF	6	1.59	0.35	0.042
6	Peroxiredoxin-1	Q06830	PRDX2	10	1.75	0.36	0.001
7	Peroxiredoxin-2	P32119	PRDX1	6	1.76	0.14	0.003

	**after treatment with rTcdA wt, 5 h**						

**Nr**.	**Protein name**	**Accession number**	**Gene name**	**Number of peptides for quantitation**	**regulation factor**	**SD**	**T-test**

1	40S ribosomal protein S16	P62249	RPS16	3	0.38	0.07	0.01
2	40S ribosomal protein S17	P08708	RPS17	9	0.40	0.03	0.018
3	40S ribosomal protein S3	P23396	RPS3	5	0.35	0.02	0.004
4	40S ribosomal protein S9	P46781	RPS9	9	0.33	0.04	0.003
5	60S ribosomal protein L18	Q07020	RPL18	8	0.36	0.07	0.007
6	60S ribosomal protein L18a	Q02543	RPL18A	4	0.33	0.05	0.004
7	60S ribosomal protein L8	P62917	RPL8	5	0.22	0.02	0.024
8	60S ribosomal protein L9	P32969	RPL9	6	0.36	0.01	0.04
9	Glutamate dehydrogenase 1, mitochondrial	P00367	GLUD1	7	0.54	0.02	0.042
10	Heterogeneous nuclear ribonucleoprotein A1	P09651	HNRNPA1	8	0.50	0.11	0.024
11	Polypyrimidine tract-binding protein 1	P26599	PTBP1	5	0.57	0.2	0.03
12	Glucosidase 2 subunit alpha	Q14697	GANAB	19	1.76	0.13	<0.0001
13	Glucosidase 2 subunit beta	P14314	PRKCSH	6	1.95	0.04	0.038
14	Endoplasmin	P14625	HSP90B1	32	1.72	0.04	<0.0001
15	Protein disulfide-isomerase	P07237	P4HB	21	1.80	0.28	0.008
16	Protein disulfide-isomerase A4	P13667	PDIA4	10	1.65	0.3	0.0003

	**after treatment with mutant rTcdA, 24 h**						

**Nr**.	**Protein name**	**Accession number**	**Gene name**	**Number of peptides for quantification**	**regulation factor**	**SD**	**T-test**

1	Filamin A	P21333	FLNA	4	0.7	0.06	0.015

	**after treatment with rTcdA wt, 24 h**						

**Nr**.	**Protein name**	**Accession number**	**Gene name**	**Number of peptides for quantification**	**regulation factor (average)**	**SD**	**T-test^3^**

1	Actin, cytoplasmic 1	P60709	ACTB	46	0.57	0.25	<0.0001
2	Filamin A	P21333	FLNA	4	0.52	0.04	0.01
3	Filamin B	O75369	FLNB	65	0.6	0.14	<0.0001
4	Villin-1	P09327	VIL1	11	0.58	0.19	0.008
5	Apolipoprotein E	P02649	APOE	4	0.29	0.00	0.041
6	Creatine kinase B-type	P12277	CKB	23	0.57	0.04	<0.0001
7	Glucosidase 2 subunit beta	P14314	PRKCSH	11	0.46	0.09	0.007
8	Peroxiredoxin-1	Q06830	PRDX1	22	0.72	0.14	<0.0001
9	Peroxiredoxin-2	P32119	PRDX2	15	0.63	0.11	0.004
10	Peroxiredoxin-6	P30041	PRDX6	6	0.58	0.04	0.026
11	Annexin A3	P12429	ANXA3	11	0.68	0.13	0.006

^1 ^The change in abundance of the proteins is expressed by the calculated ratio between with rTcdA wt or mutant rTcdA treated cells and control cells of all labeled peptides and the average ratio was determined.

^2 ^SD: standard deviation

^3 ^T-test: Student's t-test was used for calculation of p-values, p < 0.05

The quantitative MS data for the proteins Rac1 and clathrin heavy chain 1 provided evidence for an up-regulation after treatment with both rTcdA wt and mutant rTcdA. Since not enough peptides for these proteins could be quantified, the statistical tests (*n *≤ 3) failed to show significance and the proteins were not included in the list of regulated proteins. However, clathrin is involved in TcdA uptake and Rac1 belongs to the main targets of TcdA in the cytoplasm. Thus, particular attention was paid to these two proteins and the MS analysis was additionally verified by western blot analysis using specific anti-clathrin heavy chain 1 and anti-Rac1 antibodies (see below).

### Quantitative proteome analysis of Caco-2 cells treated with rTcdA wt and mutant rTcdA, respectively, for 24 h

Cytosolic proteins from control cells and cells treated with either rTcdA wt or mutant rTcdA were labeled with light, heavy, and medium ICPL-reagent, respectively. Two technical replicates of the first biological replicate and one technical replicate from the second biological replicate were prepared and analyzed. 485 proteins were identified by at least two unique peptides, 420 proteins were quantified by at least one unique peptide and 286 proteins were quantified by at least two unique peptides. The false discovery rates for all replicates were 1.05%, 1.16% and 0.83% with p < 0.05. Eleven proteins were significantly down-regulated by rTcdA wt and one of them, filamin A, was down-regulated after treatment with mutant rTcdA, too (Table 1, Figure 4C, Additional file 2). Due to high similarities actin isoforms, beta-actin and gamma-actin, could not be distinguished by mass spectrometric analysis, thus both isoforms were supposed to be down-regulated in response to rTcdA wt. Four proteins were similarly regulated by both, rTcdA wt and mutant rTcdA but due to a limited amount of detectable ICPL-labeled peptides the quantitative data were just below statistical significance (data not shown). In all cases only one peptide that was labeled with the light, medium and heavy ICPL reagent was detectable. Three of these proteins, glutathione S-transferase A2, annexin A4 and Rac1 might be up-regulated whereas one protein, mitsugumin23, might be down-regulated. Thus, a western blot analysis was done at least for Rac1 using specific antibodies (see below).

### Verification of quantitative proteome data by Western Blot analyses

**Figure 5:**
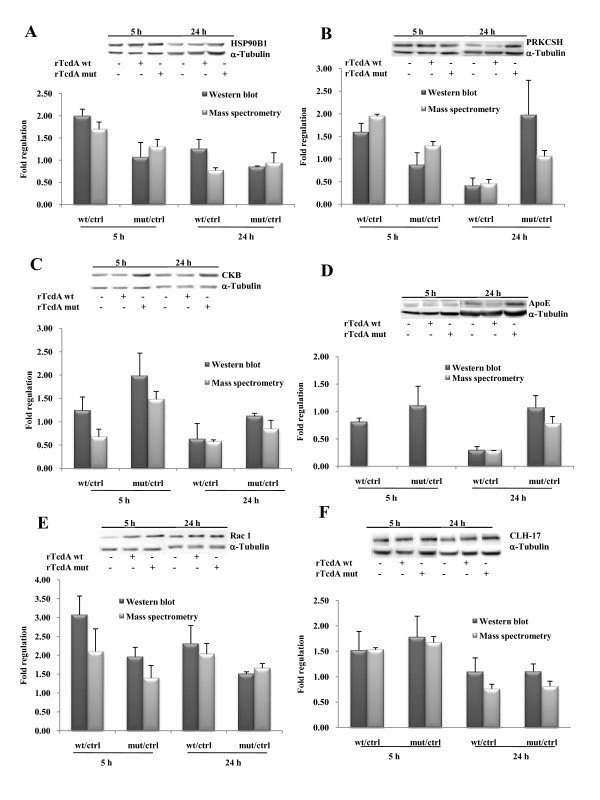
**Western blot analyses of regulated proteins**. Caco-2 cells were treated with rTcdA wt or mutant rTcdA (500 ng/mL) for 5 h or 24 h. After fractionation the cytosolic proteins were separated on 12% SDS-PAGE and probed with antibodies against regulated ptoteins. The western blot quantitation was performed on a Kodak Image Station 440 CF by analysis with the Kodak 1D Image software, for normalization of samples α-tubulin was used. The *bars *represent fold regulation factors referred to values of control samples (mean values + SD, n ≥ 3). The mass spectrometry data of corresponding proteins were shown to compare the results of this study. **A) **Endoplasmin (HSP90B1); **B) **Glucosidase 2 subunit beta (PRKCSH); **C) **Creatin kinase b-type (CKB); **D) **Apolipoprotein E (ApoE); **E) **Rac1; **F) **Clathrin, heavy chain 1 (CLH-17).

Western blot analyses were performed in order to verify the quantitative proteome data obtained by the ICPL experiments. The proteins endoplasmin (HSP90B1), glucosidase 2 subunit beta (PRKCSH), creatin kinase B (CKB), and apolipoprotein E (Apo-E) were selected for these further investigations. All these proteins showed a considerable regulation as detected using the ICPL technique. Rac1 and clathrin heavy chain (CLH-17) whose regulation was just below the significant threshold as evident after ICPL based analysis (s. above) were additionally quantified by Western bot analysis (Figure 5). Quantitative results obtained for endoplasmin and apolipoprotein E were consistent with the quantitative proteome data as shown in Figure 5A, D. Some discrepancies were observed using anti-glucosidase 2 subunit beta and anti-creatin kinase B-type antibodies (Figure 5B, C). Glucosidase 2 subunit beta seemed to be up-regulated by 2-fold after treatment with mutant rTcdA for 24 h, whereas ICPL data showed no changes in protein expression of glucosidase 2 subunit beta under these conditions. However, in cells treated with rTcdA wt the analyses using antibodies against glucosidase 2 beta fit well to the quantitative mass spectrometry data. The western blot analyses for creatin kinase B-type also indicated differences to the ICPL-based mass spectrometry data for the 5 h time point. Under these conditions creatine kinase B-type was found up-regulated by western blot analysis after treatment with mutant rTcdA but no regulation was detected using ICPL analysis (Figure 5C). The data acquired for apolipoprotein E after 24 h treatment with rTcdA wt and mutant rTcdA corresponded well to the western blot results. After 5 h treatment with rTcdA wt or mutant rTcdA apolipoprotein E was not detected by mass spectrometry; however the protein was clearly detected by western blot analysis and showed no significant changes at protein level after 5 h in the cytosolic fraction. The proteins clathrin heavy chain 1 and Rac1 were clearly identified by the mass spectrometry approach but no statistical indications for an up-regulation were found by the ICPL analysis, because only one peptide of each protein was labeled by the heavy, medium, and light ICPL-reagent (s. above). However, western blot analyses proved an up-regulation of these proteins. In the Figure 5E and 5F the values of the ICPL-labeled single peptide and the data from western blot analysis are shown.

## Discussion

The main virulence factors of *C. difficile *are the toxins TcdA and TcdB that inactivate Rho proteins in eukaryotic cells. The toxins act in the cytosol of target cells to catalyze the transfer of a glucose moiety onto a crucial threonine residue of Rho proteins. Delivery of the toxins into the cytosol and their effects on Rho proteins and Rho-dependent signaling in host cells is investigated [4,7] but proteins and pathways involved in processes downstream of Rho proteins are largely unclear at present. In particular, the question whether large clostridial cytotoxins affect target cells independently of the intrinsic glucosyltransferase activity and thus independently of Rho proteins is controversially discussed [4,16]. Hence we initiated a toxicoproteomic study to analyze cellular effects of large clostridial cytotoxins using recombinant wild type or mutant TcdA. A similar study has been conducted to elucidate the function of the C3 exoenzyme of C. botulinum where several proteins were identified to be responsive to C3 treatment in neuronal cells [17]. Thus, a comprehensive proteome analysis was conducted using the ICPL technique and LC-MALDI-MS. ICPL labeling occurs at lysine residues and prevents trypsin cleavage after these residues. Thus, the tryptic digestion was altered to an ArgC specificity leading to the generation of slightly larger peptides on average. However, quantification was only possible for half of the identified proteins. Although lysine is overrepresented in human proteins (>7%), several of the identified peptides did not contain lysine residues. Additionally, few lysine residues remained unlabeled at all or escaped labeling. For these peptides containing unlabeled lysine residues no corresponding peptides with a labeled lysine were detected. Also the opposite was not observed: for all identified ICPL-labeled peptides no unlabeled counterpart was detected. From this observation we concluded that ICPL labeling is either complete or completely lacking for a particular peptide.

Overall 30 proteins responded to the treatment with rTcdA wt or mutant rTcdA (Figure 4). Short time effects (5 h) were responsible for up- or down-regulation of sixteen proteins due to treatment with rTcdA wt; seven proteins were identified by mass spectrometry as up-regulated by mutant rTcdA (Figure 4A, B). Moreover, two proteins, Rac1 and clathrin heavy chain 1, were up-regulated by both rTcdA wt and mutant rTcdA as determined by MS and western blot techniques (Figure 5E, F). After long time treatment (24 h) with rTcdA wt, the concentration of eleven proteins was altered (Figure 4C). Additionally, one of the latter proteins, filamin A (FLNA), was significantly down-regulated after treatment with mutant rTcdA. These data strongly indicate particular effects of the catalytic inactive mutant rTcdA. This provides further evidence that large clostridial cytotoxins alter cellular processes of target cells independently of the intrinsic glucosyltransferase activity. Additionally, cellular processes independent from cytoskeleton homeostasis are influenced by the clostridial cytotoxins.

Short-time transferase-independent effects of toxins include changes in concentration of proteins involved in energy metabolism and signal transduction. Interestingly, annexin A3 (ANXA3) and the pro-inflammatory cytokine macrophage migration inhibitory factor (MIF) were up-regulated, which are known to be involved in response to bacterial pathogens [18,19]. These proteins were not up-regulated in the cells treated with rTcdA wt for 5 h, but rTcdA wt induces down regulation of annexin A3 after 24 h (Figure 4C). MIF suppresses pro-oxidative stress-induced apoptosis and contributes to the regulation of cell survival [20]. Thus, mutant rTcdA signaling further facilitates cell proliferation of Caco-2 cells and should be clearly different from that of rTcdA wt. Rac1 is a major target of rTcdA wt and was up-regulated after treatment with wild type as well as with mutant toxin. Thus, Rac1 up-regulation facilitated via a Rho-independent signaling pathway that might also adjust up-regulation of MIF, annexin A3, clathrin heavy chain 1, peroxiredoxins, metabolic enzymes, and probably other so far unrecognized proteins. Up-regulation of Rac1 and other proteins by the catalytic inactive toxin might be interpreted as a protection response of the colonic cells that is induced by so far unknown mechanisms or a general effect related to endocytotic uptake of toxin. Thus, Rho-independent signal transduction mechanism might be responsible to detect and respond to bacterial infections. Another cellular target of rTcdA wt, RhoB, is well known to be up regulated in response to TcdA wt treatment but is not induced by the catalytic inactive rTcdA mutant. RhoB induction is thought to have a proapoptotic function [11,21].

Caco-2 cells treated for 5 h with rTcdA wt responded in a considerable decrease of ribosomal proteins that are involved in ribosome function and mRNA trafficking [22]. However, ribosomal proteins possess extra-ribosomal functions [22,23]. The 40S ribosomal protein S3 (RPS3) is involved in maturation of 40S ribosomal subunit and exhibits apoptotic function [24,25]. Lee *et.al*. have shown, that RPS3 phosphorylated by Akt accumulates in the nucleus possesses reduced proapoptotic properties [26]. The proteins glutamate dehydrogenase 1, mitochondrial (GLUD1), heterogeneous nuclear ribonucleoprotein A1 (hnRNPA1), and polypyrimidine tract-binding protein 1 (PTBP1) were down regulated after short term rTcdA wt treatment (Figure 4B). The latter two proteins are also involved in mRNA metabolism, processing, and transport [27-30]. HnRNP A1 becomes localized in the cytoplasm upon transcription inhibition and belongs to transcription-sensitive group of hnR proteins. The hnRNPA1 shuttling activity is required for survival and granulocytic differentiation of normal myeloid precursors [31,32]. The cellular distribution upon transcription inhibition of PTBP1 is transcription-insensitive; the protein is localized in the nucleus [33]. The reduced concentration of ribosomal proteins was identified in the cytoplasmic protein fraction and might indicate a more nuclear localization of these proteins that can reside in both, the nuclear and the cytoplasmic compartment. Thus, a disruption of the balanced shuttling between cytoplasm and nucleus can be assumed due to the treatment with rTcdA wt. However, after 24 h treatment of Caco-2 cells with rTcdA wt no changes in expression of the ribosomal proteins, hnRNPA1, and PTBP1 were observed. Thus, the down-regulation of these proteins in cytosol is terminated as an early effect of the clostridial cytotoxins.

Protein glucosidase 2 (alpha and beta subunit, GANAB and PRKCSH), the chaperons endoplasmin (HSP90B1), protein disulfide-isomerases (P4HB and PDIA4) were induced after 5 h treatment with rTcdA wt. (Figure 4B, 5A, B). Endoplasmin is a glycoprotein [34] that has been linked to the Toll-like receptor pathway [35], response to hypoxia [36], and this protein is required for innate immunity but not cell viability [37]. It forms a large chaperone multiprotein complex with protein disulfide-isomerase A4 and other chaperonins and is essential for translocation of *C. botulinum *C2 toxin and uptake of *C. perfringens *iota toxin into eukaryotic cells [38,39]. Indeed, endoplasmin has been reported to bind to TcdA at the cell surface and is translocated together with TcdA into the cytoplasm [40]. We propose that toxin-dependent endoplasmin translocation from membrane into the cytoplasma led to an increased cytoplasmic concentration of this protein that was not observed after treatment with mutant rTcdA.

Glucosidase 2 beta catalyzes the removal of three glucose residues from the peptide-bound Glc_3_-Man_9_-GlcNAc_2 _oligosaccharide remodelling the asparagine-linked precursor [41] and UDP-glucose:glycoprotein glycosyltransferase. The latter plays a possible role in control of protein glycosylation [42] and regulation of N-glycoprotein transport or protein degradation [43,44]. These data indicate that post-translational modifications like glycosylation are influenced by large clostridial toxins. However, after 24 h treatment with rTcdA wt glucosidase 2 beta decreased by 2-fold.

After the long-term treatment of cells with rTcdA wt several cytoskeletal proteins (beta-, gamma-actin, filamine A and B, villin 1) were down regulated. This is in good accordance to the cytopathic cell rounding of rTcdA wt treated cells. Interestingly, filamine A was also down-regulated by rTcdA mutant. Thus, a Rho-independent signaling should be involved in filamine regulation.

Peroxiredoxins 1, 2, 6 were found down-regulated after treatment with rTcdA wt for 24 h; peroxiredoxins 1 and 2 were up-regulated after short time incubation with mutant rTcdA (Figure 4A, C). Kim *et.al*. reported that *C. difficile *Toxin A induces the release of reactive oxygen species (ROS) and influences the regulation of cyclooxigenase-2 and prostaglandin E_2 _synthesis by ROS [9,45]. The expression changes of peroxiredoxins are involved in redox control and might be induced after 5 h to protect cells from ROS. Down regulation of these protecting enzymes after 24 h of toxin treatment might be a hint that the cells have become more apoptotic.

The presented data indicate that large clostridial cytotoxins like TcdA act not only on Rho proteins and induce Rho-dependent pathways. Rho-independent effects evoked by a catalytic inactive toxin mutant point to new cellular target structures in eukaryotic cells and so far unknown ways of signaling. For the first time evidence has been provided that TcdA influences more cellular functions than cytoskeleton homeostasis and apoptosis. The response of colonic cells to TcdA is time-dependent and different protein patterns are evident after short (5 h) and long (24 h) incubation times. Activity and probably location of ribosomal proteins, metabolic enzymes, and protection enzymes like peroxiredoxin were modified due to action of TcdA.

## Materials and methods

### Cell culture and preparation of cellular fractions

Caco-2 cells were grown in Dulbecco's minimum essential medium (DMEM) containing 10% fetal calf serum (FCS), 100 U/ml penicillin and 0.1 mg/ml streptomycin and 1% non-essential amino acids and were maintained at 37°C in an atmosphere of 5% CO_2 _in air. Cells were subcultured twice a week and cells were grown for one day before treatment with rTcdA wt or mutant rTcdA that was deficient in glucosyltransferase activity [5]. The toxins were added to the medium in a concentration of 0.5 μg/ml (0.0016 mM). After 5 h or 24 h the medium was removed and cells were washed three times with PBS. Two biological replicates for 5 h and for 24 h samples were produced. The cells were scratched from the culture flasks with a cell scraper and resuspended in 1 ml of buffer A (10 mM HEPES, pH 7.9, 10 mM KCl, 300 mM sucrose, 1.5 mM MgCl_2_, 0.1% NP-40, 0.5 mM PMSF, 0.04% 25 × Complete EDTA-free) and cytosolic and nuclear protein preparation were performed as described [46]. Briefly, after incubation on ice for 5 minutes cell lysates were centrifugated (13 200 rpm for 30 second), so the supernatant contained cytosolic proteins. Nuclear pellet was washed by resuspending in buffer A and centrifugated, as well. Eventually, the nuclei were resuspended in 100 μL buffer B (20 mM HEPES, pH 7.9, 100 mM KCl, 100 mM NaCl, 0.5 mM PMSF, 20% (v/v) glycerol, 0.04% (v/v) 25 × Complete EDTA-free), sonicated for 10 seconds and centrifugated (13.200 rpm for 30 second). Quality of the separation was controlled by western blotting using lamin B and GAPDH as marker proteins. Protein concentration was determined using Bradford assay. The protein extracts were stored at -80°C.

### Preparation of toxins

All experiments were performed with purified, recombinant wildtype TcdA and glucosyltransferase deficient mutant TcdA expressed in *Bacillus megaterium *expression system. The His-tagged fusion proteins were purified by Ni2+ affinity chromatography [47]. The *tcdA *gene from *C. difficile *strain VPI 10463 (GenBank accession no. X51797) was used for overexpression. Purity and protein concentration of the overexpressed protein were controlled and the activity was monitored by toxin-induced cell rounding. The absence of cytotoxic impurities acting on isolated mitochondria was checked using a cytochrome *C *release assay as described previously [47,48].

### ICPL-labeling of cytosolic proteins and in-gel digestion

Before ICPL-labeling procedure acetone precipitation of proteins from each biological replicate were carried out. To one volume of sample 5 volumes of acetone cooled to -20°C were added and incubated over night at -20°C. Precipitated proteins were pelleted by centrifugation at 15,000 g at 4°C and 15 min. Pellets were washed with cold 80% (v/v) aceton (-20°C) and dried. Proteins were solved in ICPL lysis buffer containing Guanidine-HCl, protein concentration was determined using Bradford assay and the concentration was adjusted to 5 mg/ml by adding ICPL Lysis buffer. Equal amounts (100 μg) of cytosolic proteins of untreated Caco-2 cells, Caco-2 cells treated with rTcdA wt or Caco-2 cells treated with mutant rTcdA were labeled with ICPL™-Triplex reagent using the ICPL™ kit from Serva Electrophoresis GmbH (Heidelberg, Germany) as described by the manufacturer [49]. First, the cysteine residues were reduced using 0.5 μl of reduction solution (TCEP-HCl, tris(2-carbox-ethy1)phosphine, 200 mM) and alkylated using 0.5 μL of 400 mM iodoacetamide (IAA). To label the N-terminus and free amino groups at the lysine residues 3 μL of C^12^- or C^13 ^or D^2 ^- nicotinoyloxysuccinimide (Nic-reagent) were added to each mixture and incubated for 3 h in the dark. Thereafter all three ICPL labeled samples were combined and proteins were precipitated by acetone to remove excess labeling reagent (as described above). Proteins were solved in Laemmli sample buffer, separated by gradient SDS-PAGE (8.5%-18%) and stained with Coomassie brilliant blue. The gel lane was cut into 20-25 pieces and in-gel digestion was performed; proteins were destained with 50 mM NH_4_HCO_3 _in 50% (v/v) acetonitrile (ACN) [50]. Then the gel pieces were dried with 100% ACN, vacuum evaporated and rehydrated with 20 μL of 5 ng/μL modified trypsin (Promega) in 20 mM NH_4_HCO_3_, 10% ACN. After incubation on ice for 1 h, residual trypsin solution was removed and substituted by 20 mM NH_4_HCO_3 _in 10% ACN. The digestion was performed over night at 37°C. Supernatants were collected and the gel pieces were washed with 50% ACN in 0.2% (v/v) TFA and then dried in 10% (v/v) ACN. All supernatant containing peptides were pooled and dried. The dried peptide samples were stored at -80°C until analysis. For separation by high performance liquid chromatography (HPLC) samples were dissolved in 100 μL 2% (v/v) ACN in 0.1% (v/v) TFA.

### Liquid chromatography

Peptide separation was done by reversed phase chromatography using a nano-HPLC system (Dionex GmbH, Idstein, Germany) which consists of an autosampler (Famos), a loading pump (Switchos), a gradient pump (Ultimate) and a microfraction collector (Probot). An aliquot of 20 μL of each sample was injected onto a C18 trap column (PepMap 300 μm × 5 mm, 3 μm, 100 Å, Dionex) with 2% (v/v) acetonitrile in 0.1% (v/v) TFA at a flow rate of 30 μL/min. After switching the trap column into the nanoflow (200 nL/min), the peptides were eluted onto a separation column (PepMap, C18 reversed phase material, 75 μm × 150 mm, 3 μm, 100 Å, Dionex GmbH, Idstein, Germany) and separated at a flow rate of 200 nL/min using eluent A with 5% (v/v) acetonitrile in 0.1% (v/v) TFA and eluent B with 80% (v/v) acetonitrile in 0.1% (v/v) TFA with a gradient from 10% to 40% (v/v) eluent B in 134 min and 40% (v/v) to 100% (v/v) eluent B in 10 min. 384 fractions of 94 nL and spotting time of 23 s were spotted directly onto a manually prespotted MALDI Anchor Chip 600/384 target plate (Bruker Daltonics GmbH, Bremen, Germany) with a continuous sheath flow of 2.5 μL/min 5% (v/v) acetonitrile in 0.1% (v/v) TFA.

### MALDI TOF/TOF

For target preparation the α-cyano-4-hydroxy-cynnamic acid (CHCA) matrix composed of 4 mg/ml CHCA in 50% (v/v) acetonitrile, 0.1% (v/v) TFA was diluted 1:4 and 0.6 μL of this matrix solution was spotted on each spot of a MALDI Anchor Chip 600/384 target plate. After sample spotting recrystallization was performed with 0.2 μL of 5% (v/v) acetonitrile and 0.1% (v/v) TFA. For external calibration 0.3 μL of peptide calibration standard (Bruker Daltonik GmbH) was spotted onto the target calibration spots. Mass spectra were recorded with an Ultraflex TOF/TOF I mass spectrometer (Bruker Daltonics GmbH, Bremen, Germany) operating with FlexControl 2.4, FlexAnalysis 2.4, BioTools 3.0 and WARP-LC 1.1 software. To identify proteins, searches against the MSDB database (version 2007, 148 210 sequences for human) using Mascot 2.2 software (Matrix Science, in-house server) were carried out. For FDR calculation the BTTask.tas of each run was converted to .mgf by software programm TasFileMGFExporter (provided by Bruker Daltonik GmbH), all .mgf files from one sample were combined by Notepad++ v.5.6.8, and automatic decoy searches against MSDB data base using Mascot 2.2 were performed. Searches were performed considering variable modifications like oxidation of methionin, carbamidomethylation of cysteins and ICPL modifications. One missed cleavage was allowed and the mass tolerance was set to <100 ppm for precursor ions and ≤ 70 ppm for fragment ions. Peptides were termed identified if the peptide ion score was > 25 and proteins were identified with at least two unique peptides. Decoy database searches were performed with the MSDB random database and the same parameters as described above to define the false discovery rate. To calculate the heavy/light (H/L) or medium/light (M/L) ratios of a protein at least two unique peptides labeled with heavy medium and light reagent were chosen and the average ratio was determined. Mass spectral data are available under accession number 18334-18339, PRIDE database [51].

### Western blot

Mouse monoclonal anti-Rac1 (clone 23A8) was purchased from Millipore (Billerica, USA), mouse monoclonal anti-GAPDH was from Zytomed systems (Zytomed systems GmbH, Germany), mouse monoclonal anti-Rac1 (Clone 102) and mouse anti-Clathrin heavy chain 1 were from BD Transduction Laboratories (Erembodegem, Belgium), mouse monoclonal anti-α-Tubulin, rabbit polyclonal anti-Creatine kinase B-Type and rabbit polyclonal anti-HSP90B1 were from Sigma (Saint Louis, USA), mouse monoclonal anti-Glucosidase IIβ (D-1) and goat polyclonal anti-Lamin B were from Santa Cruz Biotechnology (Santa Cruz, USA) and mouse monoclonal anti-Apolipoprotein E was from MBL (Naka-Ku Nagoya, Japan). For the analysis of complete lysates or cytosolic proteins by SDS-PAGE and Western blotting, samples were dissolved in Laemmli sample buffer, incubated at 95°C for 5 min and loaded onto the gel [52]. Proteins were separated on 15, 12, or 7.5% polyacrylamide gels with 5% stacking gel. For Western blotting, proteins were transferred onto nitrocellulose membranes by a semi-dry blot system (Trans-Blot^® ^SD Semi Dry, BioRad Laboratories) for 72 min at 17 V using 25 mM Tris, 192 mM glycine buffer containing 20% (v/v) methanol. The membranes were blocked with 5% (w/v) non fat dried milk in TBST (50 mM Tris, pH 7.2, 150 mM NaCl, 0.05% (v/v) Tween 20) for 60 min and incubated with primary antibody over night at 4°C. Primary antibodies were probed with the horseradish peroxidase-conjugated secondary antibodies at room temperature for 60 min and detected by enhanced chemiluminescence reaction Femto (Pierce, Thermo Fisher Scientific Inc., Rockford, IL, USA). The western blot quantitation was performed on a Kodak Image Station 440 CF by analysis with the Kodak 1D Image software.

### Statistical analysis

The two sided Student's *t*-test was performed to describe significant regulation using peakareas of the entire isotopic cluster of labeled peptides. A value of *p *< 0.05 was accepted as significant and assigned with asterics. The *t*-test was only used for studies with *n *≥ 3 peak areas. All values are given as mean values ± standard deviation.

## Abbreviations

**CDAD**: *C. difficile*-associated diarrhea; **rTcdA**: recombinant toxin A; **ICPL™**: isotope-coded protein labeling.

## Competing interests

The authors declare that they have no competing interests.

## Authors' contributions

NJ carried out the proteomic studies, western blotting and wrote the manuscript. RG has contributed to the obtaining of the rTcdA wt and mutant rTcdA and design of the study. AP and IJ participate in the design of the study and have been involved in writing the manuscript. All the authors have read and approved the final manuscript.

## Supplementary Material

Additional file 1 **Identified proteins in Caco-2 cells using LC-MALDI-technique**. Click here for file

Additional file 2 **A summary of regulated proteins in Caco-2 cells after treatment with rTcdA wt or mutant rTcdA**. Click here for file
