# Supplementary material for: Impact of clostridial glucosylating toxins on the proteome of colonic cells determined by isotope-coded protein labeling and LC-MALDI
Source: Proteome Sci. 2011 Aug 17;9:48. doi: 10.1186/1477-5956-9-48 (PMC3176154; doi:10.1186/1477-5956-9-48)
Supplement: Additional file 1 — Identified proteins in Caco-2 cells using LC-MALDI-technique. [file 1477-5956-9-48-S1.DOCX]

**Additional file 1**

**Identified proteins in Caco-2 cells using LC-MALDI-technique**

| **Nr.** | **Protein name** | **Accession number** | **Score** | **Sequence coverage [%]** | **Number of unique peptides identified** |
| --- | --- | --- | --- | --- | --- |
| 1 | 10 kDa heat shock protein, mitochondrial | P61604 | 346 | 75.26 | 8 |
| 2 | 116 kDa U5 small nuclear ribonucleoprotein component | Q15029 | 221 | 9.16 | 8 |
| 3 | 14-3-3 protein beta/alpha | P31946 | 578 | 27.76 | 15 |
| 4 | 14-3-3 protein epsilon | P62258 | 763 | 42.49 | 14 |
| 5 | 14-3-3 protein eta | Q04917 | 349 | 30.20 | 13 |
| 6 | 14-3-3 protein gamma | P61981 | 418 | 37.80 | 10 |
| 7 | 14-3-3 protein sigma | P31947 | 298 | 21.30 | 6 |
| 8 | 14-3-3 protein theta | P27348 | 578 | 37.96 | 16 |
| 9 | 14-3-3 protein zeta/delta | P63104 | 761 | 49.39 | 20 |
| 10 | 15 kDa selenoprotein | O60613 | 51 | 8.64 | 3 |
| 11 | 1-phosphatidylinositol-3-phosphate 5-kinase | Q9Y2I7 | 40 | 2.05 | 2 |
| 12 | 1-phosphatidylinositol-4,5-bisphosphate phosphodiesterase beta-2 | Q00722 | 55 | 1.61 | 2 |
| 13 | 1-phosphatidylinositol-4,5-bisphosphate phosphodiesterase delta-3 | Q8N3E9 | 74 | 3.17 | 3 |
| 14 | 1-phosphatidylinositol-4,5-bisphosphate phosphodiesterase epsilon-1 | Q9P212 | 41 | 1.64 | 2 |
| 15 | 1-phosphatidylinositol-4,5-bisphosphate phosphodiesterase eta-1 | Q4KWH8 | 59 | 1.83 | 3 |
| 16 | 26S protease regulatory subunit 4 | P62191 | 70 | 6.14 | 3 |
| 17 | 26S protease regulatory subunit 6A | P17980 | 76 | 6.52 | 2 |
| 18 | 26S protease regulatory subunit 6B | P43686 | 203 | 10.77 | 4 |
| 19 | 26S protease regulatory subunit 7 | P35998 | 275 | 20.55 | 8 |
| 20 | 26S protease regulatory subunit 8 | P62195 | 150 | 10.72 | 3 |
| 21 | 26S proteasome non-ATPase regulatory subunit 1 | Q99460 | 216 | 7.35 | 7 |
| 22 | 26S proteasome non-ATPase regulatory subunit 11 | O00231 | 244 | 19.24 | 7 |
| 23 | 26S proteasome non-ATPase regulatory subunit 12 | O00232 | 93 | 4.40 | 3 |
| 24 | 26S proteasome non-ATPase regulatory subunit 13 | Q9UNM6 | 102 | 10.08 | 4 |
| 25 | 26S proteasome non-ATPase regulatory subunit 2 | Q13200 | 245 | 9.36 | 7 |
| 26 | 26S proteasome non-ATPase regulatory subunit 3 | O43242 | 142 | 10.30 | 6 |
| 27 | 26S proteasome non-ATPase regulatory subunit 6 | Q15008 | 283 | 24.42 | 8 |
| 28 | 26S proteasome non-ATPase regulatory subunit 7 | P51665 | 333 | 17.79 | 4 |
| 29 | 26S proteasome non-ATPase regulatory subunit 8 | P48556 | 175 | 14.40 | 5 |
| 30 | 26S proteasome non-ATPase regulatory subunit 9 | O00233 | 116 | 10.76 | 2 |
| 31 | 28S ribosomal protein S18c | Q9Y3D5 | 54 | 3.85 | 3 |
| 32 | 28S ribosomal protein S26 | Q9BYN8 | 68 | 19.02 | 5 |
| 33 | 28S ribosomal protein S29, mitochondrial | P51398 | 69 | 7.79 | 3 |
| 34 | 28S ribosomal protein S35, mitochondrial | P82673 | 72 | 19.07 | 3 |
| 35 | 28S ribosomal protein S5, mitochondrial | P82675 | 45 | 5.12 | 2 |
| 36 | 28S ribosomal protein S7, mitochondrial | Q9Y2R9 | 67 | 11.57 | 2 |
| 37 | 28S ribosomal protein S9, mitochondrial | P82933 | 90 | 6.82 | 3 |
| 38 | 2-hydroxyacyl-CoA lyase 1 | Q9UJ83 | 44 | 3.46 | 2 |
| 39 | 2-oxoglutarate dehydrogenase E1 component-like | Q9ULD0 | 44 | 2.28 | 2 |
| 40 | 39S ribosomal protein L15 | Q9P015 | 54 | 3.72 | 2 |
| 41 | 39S ribosomal protein L16, mitochondria | Q9NX20 | 58 | 8.37 | 2 |
| 42 | 39S ribosomal protein L34 | Q9BQ48 | 75 | 13.04 | 3 |
| 43 | 39S ribosomal protein L38, mitochondrial | Q96DV4 | 80 | 8.67 | 3 |
| 44 | 3-hydroxyacyl-CoA dehydrogenase type-2 | Q99714 | 94 | 7.31 | 2 |
| 45 | 3-hydroxyisobutyryl-CoA hydrolase, mitochondrial | Q6NVY1 | 332 | 24.16 | 9 |
| 46 | 3-ketoacyl-CoA thiolase, mitochondrial | P42765 | 176 | 9.57 | 5 |
| 47 | 3-ketoacyl-CoA thiolase, peroxisomal | P09110 | 91 | 8.02 | 3 |
| 48 | 3-mercaptopyruvate sulfurtransferase | P25325 | 76 | 8.78 | 3 |
| 49 | 3-oxo-5-beta-steroid 4-dehydrogenase | P51857 | 71 | 6.85 | 2 |
| 50 | 3-phosphoinositide-dependent protein kinase 1 | O15530 | 42 | 4.27 | 2 |
| 51 | 40 kDa peptidyl-prolyl cis-trans isomerase | Q08752 | 109 | 15.18 | 5 |
| 52 | 40S ribosomal protein S10 | P46783 | 178 | 20.69 | 4 |
| 53 | 40S ribosomal protein S11 | P62280 | 382 | 38.22 | 7 |
| 54 | 40S ribosomal protein S13 | P62277 | 199 | 21.85 | 4 |
| 55 | 40S ribosomal protein S14 | P62263 | 223 | 19.21 | 3 |
| 56 | 40S ribosomal protein S15 | P62841 | 151 | 22.07 | 3 |
| 57 | 40S ribosomal protein S16 | P62249 | 242 | 31.51 | 5 |
| 58 | 40S ribosomal protein S17 | P08708 | 130 | 22.22 | 3 |
| 59 | 40S ribosomal protein S18 | P62269 | 431 | 40.79 | 10 |
| 60 | 40S ribosomal protein S19 | P39019 | 225 | 22.92 | 4 |
| 61 | 40S ribosomal protein S2 | P15880 | 301 | 23.89 | 7 |
| 62 | 40S ribosomal protein S20 | P60866 | 211 | 29.41 | 4 |
| 63 | 40S ribosomal protein S23 | P62266 | 167 | 21.83 | 3 |
| 64 | 40S ribosomal protein S25 | P62851 | 193 | 20.80 | 3 |
| 65 | 40S ribosomal protein S26 | P62854 | 106 | 20.87 | 3 |
| 66 | 40S ribosomal protein S3 | P23396 | 625 | 40.74 | 14 |
| 67 | 40S ribosomal protein S3a | P61247 | 722 | 51.33 | 16 |
| 68 | 40S ribosomal protein S4, X isoform | P62701 | 678 | 38.78 | 11 |
| 69 | 40S ribosomal protein S5 | P46782 | 174 | 13.30 | 5 |
| 70 | 40S ribosomal protein S6 | P62753 | 357 | 23.29 | 7 |
| 71 | 40S ribosomal protein S7 | P62081 | 278 | 20.62 | 7 |
| 72 | 40S ribosomal protein S8 | P62241 | 287 | 22.60 | 5 |
| 73 | 40S ribosomal protein S9 | P46781 | 454 | 41.45 | 12 |
| 74 | 40S ribosomal protein SA (Colon carcinoma laminin-binding protein) | P08865 | 363 | 22.79 | 6 |
| 75 | 4F2 cell-surface antigen heavy chain | P08195 | 288 | 18.90 | 9 |
| 76 | 4-hydroxyphenylpyruvate dioxygenase-like protein | Q96IR7 | 104 | 8.36 | 3 |
| 77 | 5'(3')-deoxyribonucleotidase, mitochondrial | Q9NPB1 | 59 | 5.56 | 2 |
| 78 | 5'-3' exoribonuclease 1 | Q8IZH2 | 40 | 1.48 | 2 |
| 79 | 5'-3' exoribonuclease 2 | Q9H0D6 | 105 | 5.47 | 4 |
| 80 | 5'-AMP-activated protein kinase subunit gamma-1 | P54619 | 51 | 5.88 | 2 |
| 81 | 5-azacytidine-induced protein 1 | Q9UPN4 | 56 | 2.22 | 2 |
| 82 | 5'-nucleotidase domain-containing protein 4 | Q86YG4 | 49 | 3.37 | 2 |
| 83 | 60S acidic ribosomal protein P0 | P19889 | 198 | 12.94 | 3 |
| 84 | 60S acidic ribosomal protein P2 | P05387 | 185 | 30.43 | 2 |
| 85 | 60S ribosomal protein L10a | P62906 | 213 | 19.91 | 4 |
| 86 | 60S ribosomal protein L11 | P62913 | 79 | 8.62 | 2 |
| 87 | 60S ribosomal protein L12 | P30050 | 247 | 23.64 | 3 |
| 88 | 60S ribosomal protein L13 | P26373 | 175 | 25.12 | 6 |
| 89 | 60S ribosomal protein L13a | P40429 | 138 | 19.70 | 5 |
| 90 | 60S ribosomal protein L14 | P50914 | 264 | 26.36 | 7 |
| 91 | 60S ribosomal protein L15 | P61313 | 447 | 30.88 | 9 |
| 92 | 60S ribosomal protein L17 | P18621 | 90 | 16.30 | 4 |
| 93 | 60S ribosomal protein L18 | Q07020 | 405 | 42.78 | 8 |
| 94 | 60S ribosomal protein L18a | Q02543 | 143 | 19.32 | 4 |
| 95 | 60S ribosomal protein L21 | P46778 | 83 | 13.21 | 2 |
| 96 | 60S ribosomal protein L22 | P35268 | 201 | 28.91 | 3 |
| 97 | 60S ribosomal protein L23 | P62829 | 165 | 13.57 | 3 |
| 98 | 60S ribosomal protein L23a | P62750 | 265 | 25.00 | 5 |
| 99 | 60S ribosomal protein L24 | P83731 | 108 | 14.65 | 3 |
| 100 | 60S ribosomal protein L27 | P61353 | 63 | 14.71 | 2 |
| 101 | 60S ribosomal protein L27a | P46776 | 221 | 17.92 | 3 |
| 102 | 60S ribosomal protein L28 | P46779 | 303 | 36.76 | 7 |
| 103 | 60S ribosomal protein L29 | P47914 | 147 | 14.91 | 3 |
| 104 | 60S ribosomal protein L3 | P39023 | 321 | 19.90 | 12 |
| 105 | 60S ribosomal protein L31 | P62899 | 91 | 14.40 | 2 |
| 106 | 60S ribosomal protein L35 | P42766 | 236 | 31.97 | 4 |
| 107 | 60S ribosomal protein L3-like | Q92901 | 43 | 3.44 | 2 |
| 108 | 60S ribosomal protein L4 | P36578 | 304 | 18.54 | 8 |
| 109 | 60S ribosomal protein L5 | P46777 | 192 | 19.59 | 7 |
| 110 | 60S ribosomal protein L6 | Q02878 | 257 | 18.12 | 6 |
| 111 | 60S ribosomal protein L7 | P18124 | 550 | 32.39 | 11 |
| 112 | 60S ribosomal protein L7a | P62424 | 603 | 39.47 | 13 |
| 113 | 60S ribosomal protein L8 | P62917 | 511 | 30.35 | 10 |
| 114 | 6-phosphofructo-2-kinase/fructose-2,6-biphosphatase 3 | Q16875 | 42 | 5.60 | 2 |
| 115 | 6-phosphofructokinase type C | Q01813 | 362 | 12.24 | 10 |
| 116 | 6-phosphofructokinase, liver type | P17858 | 79 | 5.91 | 4 |
| 117 | 6-phosphofructokinase, muscle type | P08237 | 85 | 4.74 | 4 |
| 118 | 6-phosphogluconate dehydrogenase, decarboxylating | P52209 | 211 | 12.84 | 6 |
| 119 | 6-phosphogluconolactonase | O95336 | 123 | 12.02 | 2 |
| 120 | 78 kDa glucose-regulated protein | P11021 | 761 | 26.87 | 16 |
| 121 | 7-dehydrocholesterol reductase | Q9UBM7 | 117 | 8.84 | 4 |
| 122 | 80 kDa MCM3-associated protein | O60318 | 46 | 1.92 | 2 |
| 123 | A disintegrin and metalloproteinase with thrombospondin motifs 15 | Q8TE58 | 43 | 2.42 | 2 |
| 124 | A disintegrin and metalloproteinase with thrombospondin motifs 5 | Q9UNA0 | 43 | 2.58 | 2 |
| 125 | Abhydrolase domain-containing protein 11 | Q8NFV4 | 52 | 6.11 | 2 |
| 126 | Abnormal spindle-like microcephaly-associated protein | Q8IZT6 | 81 | 1.18 | 4 |
| 127 | Absent in melanoma 1-like protein | Q8N1P7 | 45 | 4.14 | 2 |
| 128 | Acetyl-CoA acetyltransferase, cytosolic variant | Q9BWD1 | 403 | 22.28 | 7 |
| 129 | Acetyl-CoA acetyltransferase, mitochondrial | P24752 | 406 | 24.36 | 9 |
| 130 | Acetyl-CoA carboxylase 1 | Q13085 | 229 | 5.37 | 6 |
| 131 | Acetyl-CoA carboxylase 2 | O00763 | 97 | 1.91 | 5 |
| 132 | Acidic leucine-rich nuclear phosphoprotein 32 family member A | P39687 | 228 | 19.14 | 5 |
| 133 | Acidic leucine-rich nuclear phosphoprotein 32 family member B | Q92688 | 218 | 13.60 | 5 |
| 134 | Aconitate hydratase, mitochondrial | Q99798 | 189 | 7.31 | 7 |
| 135 | Actin, alpha cardiac muscle 1 | P68032 | 69 | 10.88 | 3 |
| 136 | Actin, aortic smooth muscle | P62736 | 112 | 13.26 | 4 |
| 137 | Actin, cytoplasmic 1/2 | P63261 | 1494 | 56.68 | 42 |
| 138 | Actin-like protein 6A | O96019 | 56 | 8.86 | 3 |
| 139 | Actin-related protein 2 | P61160 | 99 | 9.73 | 3 |
| 140 | Actin-related protein 2/3 complex subunit 3 | O15145 | 48 | 8.99 | 2 |
| 141 | Actin-related protein 5 | Q9H9F9 | 62 | 2.86 | 3 |
| 142 | Activating transcription factor 7-interacting protein 2 | Q5U623 | 48 | 3.23 | 2 |
| 143 | Activator of 90 kDa heat shock protein ATPase homolog 1 | O95433 | 158 | 16.41 | 5 |
| 144 | Active breakpoint cluster region-related protein | Q12979 | 49 | 2.07 | 2 |
| 145 | Acylamino-acid-releasing enzyme | P13798 | 78 | 3.83 | 2 |
| 146 | Acyl-CoA synthetase short-chain family member 3 | Q9H6R3 | 43 | 3.79 | 2 |
| 147 | Acyl-protein thioesterase 1 | O75608 | 74 | 9.13 | 2 |
| 148 | Adenine phosphoribosyltransferase | P07741 | 140 | 12.85 | 2 |
| 149 | Adenomatous polyposis coli protein 2 | O95996 | 93 | 3.50 | 5 |
| 150 | Adenosine deaminase domain-containing protein 2 | Q8NCV1 | 58 | 4.06 | 2 |
| 151 | Adenosine kinase | P55263 | 91 | 3.51 | 2 |
| 152 | Adenosylhomocysteinase | P23526 | 869 | 29.47 | 16 |
| 153 | Adenylate cyclase type 1 | Q08828 | 45 | 2.58 | 2 |
| 154 | Adenylate kinase isoenzyme 2, mitochondrial | P54819 | 295 | 23.53 | 5 |
| 155 | Adenylosuccinate lyase | P30566 | 136 | 8.68 | 6 |
| 156 | Adenylosuccinate synthetase isozyme 2 | P30520 | 90 | 10.31 | 4 |
| 157 | Adenylyl cyclase-associated protein | Q01518 | 47 | 5.06 | 2 |
| 158 | ADP/ATP translocase 1 | P12235 | 285 | 17.51 | 6 |
| 159 | ADP/ATP translocase 2 | P05141 | 578 | 33.33 | 11 |
| 160 | ADP/ATP translocase 3 | P12236 | 401 | 19.13 | 8 |
| 161 | ADP/ATP translocase 4 | Q9H0C2 | 107 | 7.62 | 3 |
| 162 | ADP-ribosylation factor 1 | P84077 | 106 | 27.27 | 3 |
| 163 | ADP-ribosylation factor 4 | P18085 | 100 | 16.76 | 2 |
| 164 | ADP-ribosylation factor-like protein 13A | Q5H913 | 42 | 7.74 | 2 |
| 165 | Afadin | P55196 | 54 | 2.00 | 3 |
| 166 | Aflatoxin B1 aldehyde reductase member 2 | O43488 | 104 | 10.03 | 3 |
| 167 | Aggrecan core protein | P16112 | 44 | 5.73 | 2 |
| 168 | A-kinase anchor protein 11 | Q9UKA4 | 58 | 2.59 | 3 |
| 169 | A-kinase anchor protein 3 | O75969 | 52 | 3.52 | 2 |
| 170 | A-kinase anchor protein 4 | Q5JQC9 | 45 | 11.11 | 2 |
| 171 | A-kinase anchor protein 7 isoforms alpha and beta | O43687 | 47 | 23.46 | 2 |
| 172 | A-kinase anchor protein 9 | Q99996 | 86 | 1.87 | 5 |
| 173 | A-kinase anchor protein SPHKAP | Q2M3C7 | 44 | 1.41 | 2 |
| 174 | Alanyl-tRNA synthetase | P49588 | 1385 | 31.61 | 31 |
| 175 | Alcohol dehydrogenase [NADP+] | P14550 | 351 | 27.47 | 8 |
| 176 | Alcohol dehydrogenase class-3 | P11766 | 47 | 7.91 | 3 |
| 177 | Aldehyde dehydrogenase X, mitochondrial | P30837 | 63 | 3.68 | 2 |
| 178 | Aldehyde dehydrogenase, mitochondrial | P05091 | 458 | 18.62 | 9 |
| 179 | Aldo-keto reductase family 1 member C3 | P42330 | 521 | 29.23 | 10 |
| 180 | Aldo-keto reductase family 1 member C-like protein 1 | Q5T2L2 | 113 | 8.28 | 2 |
| 181 | Aldose 1-epimerase | Q96C23 | 247 | 15.79 | 5 |
| 182 | ALK tyrosine kinase receptor | Q9UM73 | 69 | 5.11 | 3 |
| 183 | Alpha-(1,3)-fucosyltransferase 10 | Q6P4F1 | 42 | 3.74 | 2 |
| 184 | Alpha-2-HS-glycoprotein [Precursor] | P02765 | 101 | 3.54 | 2 |
| 185 | Alpha-2-macroglobulin [Precursor] | P01023 | 45 | 1.76 | 2 |
| 186 | Alpha-2-macroglobulin receptor-associated protein | P30533 | 221 | 19.61 | 8 |
| 187 | Alpha-actinin-1 | P12814 | 792 | 24.73 | 20 |
| 188 | Alpha-actinin-2 | P35609 | 149 | 6.15 | 6 |
| 189 | Alpha-actinin-4 | O43707 | 893 | 23.27 | 23 |
| 190 | Alpha-aminoadipic semialdehyde dehydrogenase | P49419 | 68 | 3.92 | 3 |
| 191 | Alpha-aminoadipic semialdehyde synthase | Q9UDR5 | 49 | 16.36 | 2 |
| 192 | Alpha-catulin | Q9UBT7 | 200 | 6.51 | 5 |
| 193 | Alpha-centractin | P61163 | 133 | 12.50 | 4 |
| 194 | Alpha-enolase | P06733 | 1705 | 63.97 | 32 |
| 195 | Alpha-ketoglutarate-dependent dioxygenase FTO | Q9C0B1 | 43 | 6.09 | 2 |
| 196 | Alpha-methylacyl-CoA racemase | Q9UHK6 | 65 | 7.85 | 4 |
| 197 | Alpha-synuclein | P37840 | 52 | 12.14 | 2 |
| 198 | Alstrom syndrome protein 1 | Q8TCU4 | 102 | 1.20 | 5 |
| 199 | Aminoacyl tRNA synthase complex-interacting multifunctional protein 2 | Q13155 | 192 | 13.75 | 3 |
| 200 | Amphiphysin | P49418 | 62 | 5.68 | 2 |
| 201 | Amyloid beta A4 precursor protein-binding family B member 2 | Q92870 | 55 | 2.90 | 2 |
| 202 | Anamorsin | Q6FI81 | 95 | 9.81 | 3 |
| 203 | Angiomotin-like protein 2 | Q9Y2J4 | 44 | 2.70 | 2 |
| 204 | Anion exchange protein 2 | P04920 | 49 | 1.69 | 2 |
| 205 | Anion exchange protein 4 | Q96Q91 | 48 | 1.73 | 2 |
| 206 | Ankyrin repeat and LEM domain-containing protein 1 | Q8NAG6 | 49 | 3.48 | 2 |
| 207 | Ankyrin repeat and LEM domain-containing protein 2 | Q86XL3 | 49 | 9.21 | 2 |
| 208 | Ankyrin repeat and SOCS box protein 16 | Q96NS5 | 49 | 6.62 | 3 |
| 209 | Ankyrin repeat domain-containing protein 12 | Q6UB98 | 40 | 0.78 | 2 |
| 210 | Ankyrin repeat domain-containing protein 17 | O75179 | 41 | 1.17 | 2 |
| 211 | Ankyrin repeat domain-containing protein 18A | Q8IVF6 | 53 | 2.51 | 3 |
| 212 | Ankyrin repeat domain-containing protein 23 | Q86SG2 | 43 | 9.51 | 2 |
| 213 | Ankyrin repeat domain-containing protein 24 | Q8TF21 | 46 | 2.67 | 2 |
| 214 | Ankyrin repeat domain-containing protein 43 | Q2M3V2 | 55 | 16.99 | 3 |
| 215 | Ankyrin repeat domain-containing protein 57 | Q53LP3 | 53 | 5.63 | 2 |
| 216 | Ankyrin repeat-containing protein C20orf12 | Q9NVP4 | 42 | 4.36 | 2 |
| 217 | Ankyrin-3 | Q12955 | 61 | 0.71 | 3 |
| 218 | Annexin A11 | P50995 | 62 | 4.16 | 2 |
| 219 | Annexin A2 | P07355 | 426 | 24.26 | 8 |
| 220 | Annexin A3 | P12429 | 862 | 51.55 | 17 |
| 221 | Annexin A4 | P09525 | 354 | 25.79 | 8 |
| 222 | Annexin A5 | P08758 | 644 | 29.69 | 8 |
| 223 | Annexin A6 | P08133 | 153 | 8.04 | 6 |
| 224 | Annexin A7 | P20073 | 274 | 24.25 | 10 |
| 225 | Antigen KI-67 | P46013 | 72 | 1.17 | 5 |
| 226 | AP-1 complex subunit beta-1 | Q10567 | 60 | 3.47 | 3 |
| 227 | AP-1 complex subunit gamma-1 | O43747 | 56 | 2.19 | 2 |
| 228 | AP-1 complex subunit mu-1 | Q9BXS5 | 85 | 11.37 | 4 |
| 229 | AP1B1 protein | Q20WL3 | 116 | 3.30 | 3 |
| 230 | AP-2 complex subunit alpha-2 | O94973 | 109 | 2.98 | 3 |
| 231 | AP-3 complex subunit beta-1 | O00203 | 54 | 2.25 | 2 |
| 232 | AP-3 complex subunit delta-1 | O14617 | 70 | 2.89 | 2 |
| 233 | AP-4 complex subunit beta-1 | Q9Y6B7 | 53 | 2.98 | 2 |
| 234 | Apolipoprotein A-I-binding protein | Q8NCW5 | 78 | 5.56 | 2 |
| 235 | Apolipoprotein B-100 | P04114 | 81 | 1.04 | 4 |
| 236 | Apolipoprotein E | P02649 | 141 | 16.72 | 5 |
| 237 | Apolipoprotein-L domain-containing protein 1 | Q96LR9 | 46 | 10.26 | 3 |
| 238 | Apolipoprotein-L5 | Q9BWW9 | 47 | 4.62 | 3 |
| 239 | Apoptotic chromatin condensation inducer in the nucleus | Q9UKV3 | 332 | 10.00 | 8 |
| 240 | Arf-GAP with coiled-coil, ANK repeat and PH domain-containing protein 2 | Q15057 | 49 | 2.70 | 2 |
| 241 | Arf-GAP with GTPase, ANK repeat and PH domain-containing protein 3 | Q96P47 | 54 | 2.40 | 3 |
| 242 | Arginine and glutamate-rich protein 1 | Q9NWB6 | 66 | 8.42 | 3 |
| 243 | Arginine-glutamic acid dipeptide repeats protein | Q9P2R6 | 89 | 2.66 | 5 |
| 244 | Argininosuccinate synthase | P00966 | 90 | 8.01 | 4 |
| 245 | Arginyl-tRNA synthetase, cytoplasmic | P54136 | 258 | 9.55 | 6 |
| 246 | Armadillo repeat-containing protein 2 | Q8NEN0 | 51 | 2.21 | 2 |
| 247 | Armadillo repeat-containing protein 4 | Q5T2S8 | 52 | 2.80 | 2 |
| 248 | Armadillo repeat-containing protein 5 | Q96C12 | 62 | 2.87 | 3 |
| 249 | Armadillo repeat-containing X-linked protein 2 | Q7L311 | 66 | 3.48 | 2 |
| 250 | ArsA arsenite transporter, ATP-binding, homolog 1 variant | Q53FC6 | 49 | 5.46 | 2 |
| 251 | Aryl hydrocarbon receptor nuclear translocator-like protein 2 | Q8WYA1 | 43 | 5.14 | 2 |
| 252 | Asparagine synthetase domain-containing protein 1 | Q9NWL6 | 47 | 2.33 | 2 |
| 253 | Asparaginyl-tRNA synthetase, cytoplasmic | O43776 | 106 | 5.66 | 4 |
| 254 | Aspartate aminotransferase | P17174 | 271 | 23.79 | 9 |
| 255 | Aspartate aminotransferase, mitochondrial | P00505 | 353 | 23.95 | 10 |
| 256 | Aspartyl aminopeptidase | Q9ULA0 | 85 | 5.82 | 2 |
| 257 | Aspartyl-tRNA synthetase, cytoplasmic | P14868 | 657 | 30.94 | 15 |
| 258 | Ataxin-2 | Q99700 | 96 | 4.17 | 3 |
| 259 | Ataxin-2-like protein | Q8WWM7 | 45 | 3.17 | 2 |
| 260 | AT-hook-containing transcription factor | Q7Z591 | 58 | 2.84 | 3 |
| 261 | ATP synthase subunit alpha, mitochondrial | P25705 | 1287 | 35.62 | 20 |
| 262 | ATP synthase subunit b, mitochondrial | P24539 | 104 | 15.90 | 3 |
| 263 | ATP synthase subunit beta | P06576 | 897 | 33.27 | 15 |
| 264 | ATP synthase subunit O, mitochondrial | P48047 | 202 | 31.92 | 7 |
| 265 | ATP synthase subunit s-like protein | Q9NW81 | 52 | 8.50 | 2 |
| 266 | ATPase family AAA domain-containing protein 2 | Q6PL18 | 47 | 1.86 | 2 |
| 267 | ATPase family AAA domain-containing protein 3A | Q9NVI7 | 60 | 4.10 | 3 |
| 268 | ATP-binding cassette sub-family A member 13 | Q86UQ4 | 50 | 0.67 | 4 |
| 269 | ATP-binding cassette sub-family A member 2 | Q9BZC7 | 80 | 2.25 | 4 |
| 270 | ATP-binding cassette sub-family A member 7 | Q8IZY2 | 43 | 1.16 | 2 |
| 271 | ATP-binding cassette sub-family B member 10 | Q9NRK6 | 62 | 6.38 | 4 |
| 272 | ATP-binding cassette sub-family B member 8 | Q9NUT2 | 61 | 2.58 | 4 |
| 273 | ATP-binding cassette sub-family D member 3 | P28288 | 50 | 3.64 | 2 |
| 274 | ATP-binding cassette sub-family E member 1 | P61221 | 103 | 7.46 | 3 |
| 275 | ATP-binding cassette sub-family F member 1 | Q8NE71 | 108 | 4.96 | 4 |
| 276 | ATP-citrate synthase | P53396 | 996 | 23.13 | 25 |
| 277 | ATP-dependent Clp protease ATP-binding subunit clpX-like | O76031 | 48 | 5.53 | 3 |
| 278 | ATP-dependent DNA helicase 2 subunit 1 | P12956 | 307 | 11.33 | 6 |
| 279 | ATP-dependent DNA helicase 2 subunit 2 | P13010 | 744 | 20.25 | 17 |
| 280 | ATP-dependent DNA helicase PIF1 | Q9H611 | 43 | 2.96 | 2 |
| 281 | ATP-dependent DNA helicase Q1 | P46063 | 51 | 3.34 | 2 |
| 282 | ATP-dependent RNA helicase A | Q08211 | 1066 | 21.57 | 23 |
| 283 | ATP-dependent RNA helicase DDX1 | Q92499 | 203 | 10.62 | 9 |
| 284 | ATP-dependent RNA helicase DDX39 | O00148 | 319 | 14.99 | 6 |
| 285 | ATP-dependent RNA helicase DDX3X | O00571 | 100 | 5.75 | 4 |
| 286 | ATP-dependent RNA helicase DDX42 | Q86XP3 | 117 | 5.44 | 3 |
| 287 | ATP-dependent RNA helicase DDX50 | Q9BQ39 | 142 | 8.82 | 6 |
| 288 | ATP-dependent RNA helicase DDX54 | Q8TDD1 | 95 | 3.29 | 3 |
| 289 | Atrial natriuretic peptide-converting enzyme | Q9Y5Q5 | 72 | 4.03 | 4 |
| 290 | AT-rich interactive domain-containing protein 1B | Q8NFD5 | 40 | 1.43 | 2 |
| 291 | AT-rich interactive domain-containing protein 3A | Q99856 | 61 | 7.30 | 3 |
| 292 | AT-rich interactive domain-containing protein 3B | Q8IVW6 | 50 | 5.16 | 2 |
| 293 | Axin-2 | Q9Y2T1 | 54 | 2.73 | 2 |
| 294 | Axonemal dynein light intermediate polypeptide 1 | O14645 | 43 | 8.95 | 2 |
| 295 | Baculoviral IAP repeat-containing protein 6 | Q9NR09 | 85 | 0.95 | 6 |
| 296 | BAG family molecular chaperone regulator 2 | O95816 | 55 | 8.53 | 2 |
| 297 | BAI1-associated protein 3 | O94812 | 44 | 1.35 | 2 |
| 298 | Band 4.1-like protein 2 | O43491 | 123 | 3.68 | 2 |
| 299 | Basement membrane-specific heparan sulfate proteoglycan core protein | P98160 | 61 | 0.98 | 3 |
| 300 | Basic fibroblast growth factor receptor 1 | P11362 | 53 | 1.95 | 2 |
| 301 | Basic leucine zipper and W2 domain-containing protein 2 | Q9Y6E2 | 109 | 7.69 | 3 |
| 302 | B-cell receptor CD22 | P20273 | 118 | 5.78 | 2 |
| 303 | Bcl-2-associated transcription factor 1 | Q9NYF8 | 282 | 14.41 | 6 |
| 304 | Bcl-2-related proline-rich protein | Q9HB09 | 43 | 18.48 | 2 |
| 305 | BCL-6 corepressor-like protein 1 | Q5H9F3 | 48 | 2.45 | 3 |
| 306 | Beta-1,4-galactosyltransferase 7 | Q9UBV7 | 44 | 17.32 | 2 |
| 307 | Beta-actin-like protein 2 | Q562R1 | 156 | 57.28 | 3 |
| 308 | Beta-centractin | P42025 | 73 | 10.64 | 3 |
| 309 | Beta-enolase | P13929 | 302 | 17.55 | 4 |
| 310 | Beta-type platelet-derived growth factor receptor [Precursor] | P09619 | 46 | 1.63 | 2 |
| 311 | BH3-interacting domain death agonist | P55957 | 49 | 16.27 | 4 |
| 312 | BH3-like motif-containing cell death inducer | Q8IZY5 | 51 | 19.44 | 2 |
| 313 | Bicaudal D-related protein 1 | Q6ZP65 | 57 | 6.10 | 2 |
| 314 | Bifunctional aminoacyl-tRNA synthetase | P07814 | 829 | 13.26 | 18 |
| 315 | Bifunctional heparan sulfate N-deacetylase/N-sulfotransferase 4 | Q9H3R1 | 50 | 2.29 | 2 |
| 316 | Bifunctional purine biosynthesis protein PURH | P31939 | 523 | 25.84 | 16 |
| 317 | Bone morphogenetic protein 3b | P55107 | 43 | 4.18 | 2 |
| 318 | Brain acid soluble protein 1 | P80723 | 49 | 6.17 | 2 |
| 319 | Brain-specific angiogenesis inhibitor 1 | O14514 | 54 | 1.20 | 2 |
| 320 | Branched-chain-amino-acid aminotransferase, mitochondrial | O15382 | 78 | 4.75 | 3 |
| 321 | Breast cancer type 1 susceptibility protein | P38398 | 58 | 2.73 | 3 |
| 322 | Bromodomain adjacent to zinc finger domain protein 1A | Q9NRL2 | 74 | 1.16 | 2 |
| 323 | Bromodomain adjacent to zinc finger domain protein 2B | Q9UIF8 | 57 | 2.14 | 2 |
| 324 | Bromodomain and WD repeat-containing protein 1 | Q9NSI6 | 81 | 2.20 | 5 |
| 325 | Bromodomain-containing protein 2 | P25440 | 422 | 35.17 | 8 |
| 326 | Bromodomain-containing protein 4 | O60885 | 54 | 1.73 | 3 |
| 327 | BTB/POZ domain-containing protein 12 | Q8IY92 | 54 | 1.30 | 2 |
| 328 | BUD13 homolog | Q9BRD0 | 53 | 6.78 | 3 |
| 329 | Bullous pemphigoid antigen 1 | O94833 | 82 | 1.16 | 5 |
| 330 | Bullous pemphigoid antigen 1, isoforms 1/2/3/4/5/8 | Q03001 | 55 | 0.59 | 2 |
| 331 | Butyrate response factor 1 | Q07352 | 47 | 3.85 | 2 |
| 332 | Butyrophilin | P78409 | 44 | 1.37 | 2 |
| 333 | C-1-tetrahydrofolate synthase | P11586 | 656 | 17.34 | 19 |
| 334 | CAD protein | P27708 | 867 | 12.74 | 22 |
| 335 | Cadherin 17 | Q2M2E0 | 325 | 14.18 | 10 |
| 336 | Calcium activated chloride channel family member 4 | Q6UX81 | 52 | 2.39 | 2 |
| 337 | Calcium binding protein 1 | Q9NZU7 | 54 | 7.35 | 3 |
| 338 | Calcium-activated chloride channel protein 2 | Q9UNF7 | 49 | 3.05 | 3 |
| 339 | Calcium-activated potassium channel subunit alpha-1 | Q12791 | 53 | 4.41 | 3 |
| 340 | Calcium-binding mitochondrial carrier protein Aralar2 | Q9UJS0 | 133 | 6.67 | 4 |
| 341 | Calcium-binding mitochondrial carrier protein SCaMC-1 | Q6NUK1 | 45 | 4.61 | 2 |
| 342 | Calcium-binding mitochondrial carrier protein SCaMC-2 | Q6KCM7 | 47 | 3.70 | 2 |
| 343 | Calcium-binding mitochondrial carrier protein SCaMC-3 | Q9BV35 | 56 | 6.22 | 3 |
| 344 | Calcium-binding protein 39-like | Q9H9S4 | 80 | 13.21 | 4 |
| 345 | Calcium-dependent secretion activator 2 | Q86UW7 | 84 | 4.33 | 3 |
| 346 | Calcyclin-binding protein | Q9HB71 | 85 | 8.77 | 2 |
| 347 | Calmodulin-like protein 4 | Q96GE6 | 55 | 8.98 | 2 |
| 348 | Calmodulin-regulated spectrin-associated protein 3 | Q9P1Y5 | 176 | 7.53 | 5 |
| 349 | Calnexin | P27824 | 318 | 11.32 | 7 |
| 350 | Calpain 1 catalytic subunit | P07384 | 43 | 2.15 | 2 |
| 351 | Calpain 11 | Q5T3G1 | 52 | 2.26 | 2 |
| 352 | Calpain 13 | Q6MZZ7 | 43 | 2.99 | 2 |
| 353 | Calpain small subunit 1 | P04632 | 89 | 8.96 | 2 |
| 354 | Calpain-10 | Q9HC96 | 44 | 2.53 | 2 |
| 355 | Calponin 2 | Q99439 | 86 | 12.66 | 4 |
| 356 | Calponin 3 | Q15417 | 309 | 22.49 | 13 |
| 357 | Calsyntenin-1 | O94985 | 54 | 15.94 | 3 |
| 358 | Calumenin | O43852 | 143 | 6.98 | 3 |
| 359 | cAMP-dependent protein kinase catalytic subunit alpha | P17612 | 50 | 5.43 | 2 |
| 360 | CAP-Gly domain-containing linker protein 1 | P30622 | 44 | 2.76 | 2 |
| 361 | CAP-Gly domain-containing linker protein 2 | Q9UDT6 | 59 | 2.77 | 3 |
| 362 | Caprin-1 | Q14444 | 82 | 4.03 | 4 |
| 363 | Carbamoyl-phosphate synthase | P31327 | 51 | 3.40 | 2 |
| 364 | Carbohydrate sulfotransferase 14 | Q8NCH0 | 49 | 6.51 | 2 |
| 365 | Carbonic anhydrase 2 | P00918 | 47 | 8.20 | 2 |
| 366 | Carbonyl reductase [NADPH] 1 | P16152 | 234 | 17.03 | 4 |
| 367 | Carbonyl reductase [NADPH] 3 | O75828 | 194 | 17.03 | 4 |
| 368 | Carboxypeptidase D | O75976 | 53 | 1.88 | 3 |
| 369 | Carnitine O-acetyltransferase | P43155 | 85 | 5.93 | 7 |
| 370 | Cartilage intermediate layer protein 2 | Q8IUL8 | 63 | 2.08 | 2 |
| 371 | Casein kinase II subunit alpha' | P19784 | 161 | 10.57 | 4 |
| 372 | Casein kinase II subunit beta | P67870 | 95 | 10.75 | 2 |
| 373 | Caskin-1 | Q8WXD9 | 65 | 2.60 | 3 |
| 374 | CASP8-associated protein 2 | Q9UKL3 | 73 | 2.44 | 4 |
| 375 | Caspase recruitment domain-containing protein 11 | Q9BXL7 | 65 | 3.64 | 3 |
| 376 | Cat eye syndrome critical region protein 5 | Q9BXW7 | 378 | 18.07 | 7 |
| 377 | Catenin alpha-3 | Q9UI47 | 49 | 7.45 | 3 |
| 378 | Catenin delta-1 | O60716 | 487 | 13.93 | 13 |
| 379 | Cathepsin S | P25774 | 43 | 4.45 | 2 |
| 380 | Cation-independent mannose-6-phosphate receptor | P11717 | 49 | 1.16 | 3 |
| 381 | CCR4-NOT transcription complex subunit 1 | A5YKK6 | 100 | 2.19 | 2 |
| 382 | CCR4-NOT transcription complex subunit 4 | O95628 | 60 | 3.27 | 2 |
| 383 | CDK5 regulatory subunit-associated protein 1 | Q96SZ6 | 48 | 1.79 | 2 |
| 384 | CDKN2A-interacting protein | Q9NXV6 | 43 | 3.01 | 2 |
| 385 | Cell cycle-related kinase | Q8IZL9 | 70 | 7.64 | 2 |
| 386 | Cell division control protein 2 homolog | P06493 | 328 | 18.48 | 6 |
| 387 | Cell division cycle 5-like protein | Q99459 | 164 | 7.62 | 6 |
| 388 | Cell division protein kinase 12 | Q9NYV4 | 41 | 1.41 | 2 |
| 389 | Cell division protein kinase 13 | Q14004 | 68 | 1.39 | 2 |
| 390 | Cell division protein kinase 14 | O94921 | 70 | 4.26 | 2 |
| 391 | Cell division protein kinase 18 | Q07002 | 93 | 4.45 | 2 |
| 392 | Cell division protein kinase 4 | Q6FG61 | 44 | 6.60 | 2 |
| 393 | Cell division protein kinase 6 | Q00534 | 53 | 6.56 | 2 |
| 394 | Cellular retinoic acid-binding protein 1 | P29762 | 138 | 27.21 | 2 |
| 395 | Centriolin | Q7Z7A1 | 48 | 3.11 | 2 |
| 396 | Centromere protein F | P49454 | 89 | 2.15 | 6 |
| 397 | Centromere protein V | Q7Z7K6 | 240 | 21.40 | 4 |
| 398 | Centromere-associated protein E | Q02224 | 92 | 1.62 | 4 |
| 399 | Centrosomal protein of 152 kDa | O94986 | 86 | 2.72 | 4 |
| 400 | Centrosomal protein of 290 kDa | O15078 | 61 | 1.41 | 3 |
| 401 | Centrosome-associated protein CEP250 | Q9BV73 | 70 | 1.52 | 3 |
| 402 | Cerebellar degeneration-related protein 2-like | Q86X02 | 69 | 8.60 | 4 |
| 403 | cGMP-dependent protein kinase 1, alpha isozyme | Q13976 | 52 | 6.57 | 3 |
| 404 | Charged multivesicular body protein 1b | Q7LBR1 | 108 | 9.18 | 3 |
| 405 | Charged multivesicular body protein 2b | Q9UQN3 | 55 | 8.45 | 2 |
| 406 | Charged multivesicular body protein 4b | Q9H444 | 62 | 8.48 | 2 |
| 407 | Chitinase domain-containing protein 1 | Q9BWS9 | 87 | 12.47 | 4 |
| 408 | Chloride channel protein 6 | P51797 | 48 | 6.25 | 2 |
| 409 | Chloride intracellular channel protein 1 | O00299 | 579 | 34.58 | 9 |
| 410 | Chloride intracellular channel protein 4 | Q9Y696 | 162 | 14.29 | 4 |
| 411 | Choline dehydrogenase, mitochondrial | Q8NE62 | 46 | 4.77 | 2 |
| 412 | Chordin | Q9H2X0 | 52 | 3.88 | 4 |
| 413 | Chromobox protein homolog 1 | P83916 | 47 | 4.32 | 2 |
| 414 | Chromobox protein homolog 2 | Q14781 | 41 | 3.57 | 2 |
| 415 | Chromobox protein homolog 5 | P45973 | 47 | 4.19 | 2 |
| 416 | Chromodomain helicase-DNA-binding protein 3 | Q12873 | 104 | 3.24 | 6 |
| 417 | Chromodomain-helicase-DNA-binding protein 3 | Q12873 | 74 | 4.99 | 3 |
| 418 | Chromodomain-helicase-DNA-binding protein 5 | Q8TDI0 | 91 | 3.36 | 2 |
| 419 | Chromodomain-helicase-DNA-binding protein 9 | Q3L8U1 | 50 | 0.98 | 3 |
| 420 | Cingulin | Q9P2M7 | 472 | 13.03 | 10 |
| 421 | Citrate synthase, mitochondrial | O75390 | 222 | 16.14 | 7 |
| 422 | Citron Rho-interacting kinase | O14578 | 63 | 1.11 | 3 |
| 423 | C-jun-amino-terminal kinase-interacting protein 4 | O60271 | 65 | 3.63 | 2 |
| 424 | Clathrin heavy chain 1 | Q00610 | 3216 | 34.29 | 53 |
| 425 | Clathrin light chain A | P09496 | 93 | 11.02 | 3 |
| 426 | Cleavage and polyadenylation specificity factor subunit 1 | Q10570 | 248 | 6.31 | 5 |
| 427 | Cleavage and polyadenylation specificity factor subunit 5 | O43809 | 100 | 13.22 | 3 |
| 428 | Cleavage and polyadenylation specificity factor subunit 6 | Q16630 | 47 | 3.91 | 2 |
| 429 | CLIP-associating protein 1 | Q7Z460 | 61 | 2.83 | 3 |
| 430 | CLIP-associating protein 2 | O75122 | 63 | 3.17 | 3 |
| 431 | CMP-N-acetylneuraminate-beta-galactosamide-alpha-2,3-sialyltransferase 4 | Q11206 | 51 | 6.40 | 2 |
| 432 | Coagulation factor XIII A chain | P00488 | 49 | 2.76 | 2 |
| 433 | Coatomer subunit alpha | P53621 | 482 | 14.22 | 11 |
| 434 | Coatomer subunit beta | P53618 | 342 | 9.83 | 9 |
| 435 | Coatomer subunit delta | P48444 | 226 | 11.96 | 7 |
| 436 | Coatomer subunit gamma | Q9Y678 | 504 | 16.70 | 13 |
| 437 | Cofilin-1 | P23528 | 123 | 13.33 | 3 |
| 438 | Coiled-coil alpha-helical rod protein 1 | Q8TD31 | 124 | 5.37 | 5 |
| 439 | Coiled-coil and C2 domain-containing protein 1A | Q6P1N0 | 62 | 3.58 | 4 |
| 440 | Coiled-coil domain-containing protein 109A | Q8NE86 | 64 | 5.98 | 2 |
| 441 | Coiled-coil domain-containing protein 120 | Q96HB5 | 42 | 2.64 | 2 |
| 442 | Coiled-coil domain-containing protein 14 | Q49A88 | 53 | 3.28 | 2 |
| 443 | Coiled-coil domain-containing protein 144A | A2RUR9 | 42 | 3.12 | 2 |
| 444 | Coiled-coil domain-containing protein 144B | Q3MJ40 | 48 | 1.93 | 2 |
| 445 | Coiled-coil domain-containing protein 146 | Q8IYE0 | 55 | 2.62 | 3 |
| 446 | Coiled-coil domain-containing protein 18 | Q5T9S5 | 46 | 2.76 | 2 |
| 447 | Coiled-coil domain-containing protein 24 | Q8N4L8 | 41 | 5.86 | 2 |
| 448 | Coiled-coil domain-containing protein 43 | Q96MW1 | 78 | 11.45 | 3 |
| 449 | Coiled-coil domain-containing protein 50 | Q8IVM0 | 44 | 4.77 | 2 |
| 450 | Coiled-coil domain-containing protein 57 | Q2TAC2 | 53 | 2.51 | 2 |
| 451 | Coiled-coil domain-containing protein 87 | Q9NVE4 | 43 | 2.71 | 2 |
| 452 | Coiled-coil-helix-coiled-coil-helix domain-containing protein 3, mitochondrial | Q9NX63 | 52 | 10.57 | 2 |
| 453 | Cold shock domain-containing protein E1 | O75534 | 229 | 10.56 | 9 |
| 454 | Collagen alpha-1(I) chain | P02452 | 70 | 4.52 | 3 |
| 455 | Collagen alpha-1(IX) chain | P20849 | 51 | 3.69 | 3 |
| 456 | Collagen alpha-1(XI) chain | P12107 | 53 | 1.16 | 2 |
| 457 | Collagen alpha-1(XII) chain | Q99715 | 51 | 0.78 | 2 |
| 458 | Collagen alpha-1(XIV) chain | Q05707 | 44 | 2.37 | 2 |
| 459 | Collagen alpha-1(XXII) chain | Q8NFW1 | 47 | 1.54 | 2 |
| 460 | Colorectal mutant cancer protein | P23508 | 42 | 2.26 | 2 |
| 461 | Complement C4-B | P0C0L5 | 60 | 2.06 | 3 |
| 462 | Complement factor H-related protein 5 | Q9BXR6 | 75 | 4.57 | 3 |
| 463 | Condensin complex subunit 1 | Q15021 | 137 | 5.19 | 4 |
| 464 | Condensin complex subunit 3 | Q9BPX3 | 61 | 2.27 | 2 |
| 465 | Contactin-5 | O94779 | 50 | 4.70 | 3 |
| 466 | Contactin-associated protein-like 3B | Q96NU0 | 68 | 3.42 | 3 |
| 467 | COP9 signalosome complex subunit 4 | Q9BT78 | 43 | 4.68 | 2 |
| 468 | Copine-7 | Q9UBL6 | 53 | 3.05 | 2 |
| 469 | Copper-transporting ATPase 2 | P35670 | 49 | 5.25 | 3 |
| 470 | Coproporphyrinogen oxidase variant | Q53F08 | 136 | 8.37 | 3 |
| 471 | Core histone macro-H2A.1 | O75367 | 509 | 31.54 | 9 |
| 472 | Core-binding factor subunit beta | Q13951 | 67 | 13.90 | 2 |
| 473 | Coronin-6 | Q6QEF8 | 46 | 3.85 | 2 |
| 474 | Coronin-7 | P57737 | 48 | 2.81 | 2 |
| 475 | Cortactin-binding protein 2 | Q8WZ74 | 48 | 1.62 | 3 |
| 476 | Creatine kinase B-type | P12277 | 1076 | 51.18 | 22 |
| 477 | Creatine kinase U-type, mitochondrial | P12532 | 68 | 27.42 | 3 |
| 478 | Crk-like protein | P46109 | 68 | 9.24 | 2 |
| 479 | CUB and sushi domain-containing protein 3 | Q7Z407 | 48 | 0.57 | 2 |
| 480 | CUGBP Elav-like family member 5 | Q8N6W0 | 75 | 14.40 | 4 |
| 481 | Cullin-9 | Q8IWT3 | 40 | 1.03 | 2 |
| 482 | Cullin-associated NEDD8-dissociated protein 1 | Q86VP6 | 278 | 8.02 | 6 |
| 483 | Cullin-associated NEDD8-dissociated protein 2 | O75155 | 141 | 6.61 | 5 |
| 484 | Cyclin-A1 | P78396 | 41 | 5.81 | 2 |
| 485 | Cyclin-dependent kinase inhibitor 2A, isoform 4 | Q8N726 | 50 | 15.64 | 2 |
| 486 | Cyclin-dependent kinase inhibitor 2A, isoforms 1/2/3 | P42771 | 53 | 16.89 | 3 |
| 487 | Cyclin-dependent kinase-like 1 | Q00532 | 46 | 1.91 | 2 |
| 488 | Cystathionine beta-synthase | P35520 | 46 | 3.64 | 2 |
| 489 | Cysteine and histidine-rich domain-containing protein 1 | Q9UHD1 | 408 | 28.61 | 10 |
| 490 | Cytochrome b-c1 complex subunit 1, mitochondrial | P31930 | 116 | 7.71 | 3 |
| 491 | Cytochrome b-c1 complex subunit 2, mitochondrial | P22695 | 81 | 13.47 | 4 |
| 492 | Cytochrome c1, heme protein, mitochondrial | P08574 | 158 | 16.31 | 5 |
| 493 | Cytohesin-4 | Q9UIA0 | 47 | 6.35 | 2 |
| 494 | Cytoplasmic FMR1-interacting protein 1 | Q7L576 | 231 | 8.94 | 5 |
| 495 | Cytoskeleton-associated protein 2-like | Q8IYA6 | 42 | 3.89 | 2 |
| 496 | Cytoskeleton-associated protein 5 | Q14008 | 144 | 2.85 | 4 |
| 497 | Cytosol aminopeptidase | P28838 | 195 | 10.27 | 5 |
| 498 | Cytosolic acyl coenzyme A thioester hydrolase | O00154 | 117 | 13.07 | 4 |
| 499 | Cytospin-B | Q5M775 | 49 | 1.97 | 2 |
| 500 | D-3-phosphoglycerate dehydrogenase | O43175 | 430 | 21.24 | 10 |
| 501 | DBF4-type zinc finger-containing protein 2 | Q9HCK1 | 49 | 1.65 | 2 |
| 502 | DCC-interacting protein 13-alpha | Q9UKG1 | 54 | 3.39 | 2 |
| 503 | DCN1-like protein 1 | Q96GG9 | 72 | 6.95 | 2 |
| 504 | DCN1-like protein 5 | Q9BTE7 | 57 | 9.82 | 3 |
| 505 | Decaprenyl-diphosphate synthase subunit 2 | Q86YH6 | 40 | 6.27 | 2 |
| 506 | Dedicator of cytokinesis 7 | Q96N67 | 109 | 3.51 | 2 |
| 507 | Dedicator of cytokinesis protein 4 | Q8N1I0 | 69 | 1.48 | 3 |
| 508 | Dedicator of cytokinesis protein 9 | Q9BZ29 | 61 | 1.66 | 3 |
| 509 | Deleted in malignant brain tumors 1 protein | Q9UGM3 | 42 | 1.24 | 4 |
| 510 | Delta(3,5)-Delta(2,4)-dienoyl-CoA isomerase, mitochondrial | Q13011 | 281 | 17.07 | 5 |
| 511 | Delta-1-pyrroline-5-carboxylate dehydrogenase, mitochondrial | P30038 | 42 | 3.91 | 2 |
| 512 | Delta-1-pyrroline-5-carboxylate synthase | P54886 | 185 | 8.30 | 7 |
| 513 | Denticleless protein homolog | Q9NZJ0 | 59 | 2.33 | 2 |
| 514 | Deoxyguanosine kinase, mitochondrial | Q16854 | 41 | 7.22 | 2 |
| 515 | Deoxyuridine triphosphatase (DUTP pyrophosphatase, Deoxyuridine 5'-triphosphate nucleotidohydrolase, mitochondrialisoform CRA_b) | P33316 | 66 | 7.14 | 2 |
| 516 | Desmin | P17661 | 143 | 4.05 | 3 |
| 517 | Desmoglein 1 | Q02413 | 42 | 2.10 | 2 |
| 518 | Desmoplakin | P15924 | 1033 | 15.22 | 28 |
| 519 | Developmentally-regulated GTP-binding protein 1 | Q9Y295 | 198 | 17.17 | 6 |
| 520 | Dexamethasone-induced Ras-related protein 1 | Q9Y272 | 60 | 14.29 | 3 |
| 521 | Diablo homolog, mitochondrial | Q9NR28 | 70 | 11.79 | 2 |
| 522 | Diacylglycerol kinase zeta | Q13574 | 52 | 3.55 | 2 |
| 523 | Dickkopf-related protein 1 | O94907 | 52 | 7.52 | 2 |
| 524 | Digestive organ expansion factor homolog | Q68CQ4 | 60 | 3.44 | 4 |
| 525 | Dihydrolipoyl dehydrogenase, mitochondrial | P09622 | 54 | 7.27 | 3 |
| 526 | Dihydrolipoyllysine-residue acetyltransferase component of pyruvate dehydrogenase complex | P10515 | 69 | 3.90 | 3 |
| 527 | Dihydrolipoyllysine-residue succinyltransferase component of 2-oxoglutarate dehydrogenase complex | P36957 | 160 | 9.49 | 4 |
| 528 | Dihydropyrimidinase | Q14117 | 45 | 8.04 | 2 |
| 529 | Dihydropyrimidinase-related protein 2 | Q16555 | 338 | 18.18 | 8 |
| 530 | Dipeptidyl peptidase 4 | P27487 | 566 | 17.89 | 14 |
| 531 | Diphosphomevalonate decarboxylase | P53602 | 110 | 14.75 | 6 |
| 532 | Disintegrin and metalloproteinase domain-containing protein 8 | P78325 | 61 | 2.79 | 2 |
| 533 | Disks large homolog 5 | Q8TDM6 | 48 | 13.58 | 2 |
| 534 | DNA damage-binding protein 1 | Q16531 | 120 | 4.39 | 7 |
| 535 | DNA helicase B | Q8NG08 | 43 | 2.85 | 2 |
| 536 | DNA mismatch repair protein Msh2 | P43246 | 94 | 3.99 | 4 |
| 537 | DNA mismatch repair protein Msh6 | P52701 | 314 | 7.94 | 6 |
| 538 | DNA polymerase epsilon catalytic subunit A | Q07864 | 89 | 2.04 | 4 |
| 539 | DNA polymerase iota | Q9UNA4 | 70 | 4.48 | 4 |
| 540 | DNA repair protein RAD50 | Q92878 | 165 | 6.48 | 3 |
| 541 | DNA repair protein XRCC2 | O43543 | 62 | 8.58 | 3 |
| 542 | DNA replication licensing factor MCM2 | P49736 | 526 | 20.02 | 12 |
| 543 | DNA replication licensing factor MCM3 | P25205 | 163 | 9.53 | 7 |
| 544 | DNA replication licensing factor MCM4 | P33991 | 323 | 14.02 | 12 |
| 545 | DNA replication licensing factor MCM5 | P33992 | 323 | 16.62 | 12 |
| 546 | DNA replication licensing factor MCM6 | Q14566 | 352 | 13.63 | 10 |
| 547 | DNA replication licensing factor MCM7 | P33993 | 245 | 17.31 | 7 |
| 548 | DNA topoisomerase 1 | P11387 | 209 | 7.75 | 6 |
| 549 | DNA topoisomerase 2-alpha | P11388 | 182 | 3.14 | 4 |
| 550 | DNA topoisomerase 2-binding protein 1 | Q92547 | 41 | 1.31 | 2 |
| 551 | DNA-(apurinic or apyrimidinic site) lyase | P27695 | 200 | 16.35 | 4 |
| 552 | DNA2-like helicase | P51530 | 41 | 1.93 | 2 |
| 553 | DNA-binding protein SATB1 | Q01826 | 41 | 3.64 | 2 |
| 554 | DNA-binding protein SMUBP-2 | P38935 | 55 | 3.92 | 3 |
| 555 | DNA-dependent protein kinase catalytic subunit | P78527 | 732 | 9.08 | 22 |
| 556 | DNA-directed RNA polymerase I subunit RPA49 | Q9GZS1 | 46 | 3.12 | 2 |
| 557 | DnaJ homolog subfamily B member 1 | P25685 | 243 | 16.22 | 6 |
| 558 | DnaJ homolog subfamily B member 12 | Q9NXW2 | 54 | 7.20 | 2 |
| 559 | DnaJ homolog subfamily B member 9 | Q9UBS3 | 42 | 8.52 | 2 |
| 560 | DnaJ homolog subfamily C member 10 | Q8IXB1 | 73 | 5.09 | 4 |
| 561 | DnaJ homolog subfamily C member 13 (HSP40) | O75165 | 126 | 2.76 | 4 |
| 562 | DnaJ homolog subfamily C member 9 | Q8WXX5 | 68 | 7.69 | 2 |
| 563 | DNA-repair protein complementing XP-C cells | Q01831 | 44 | 3.30 | 2 |
| 564 | Dolichyl-diphosphooligosaccharide-protein glycosyltransferase | P39656 | 51 | 11.84 | 5 |
| 565 | Dolichyl-diphosphooligosaccharide--protein glycosyltransferase subunit 1 | P04843 | 448 | 17.46 | 9 |
| 566 | Doublesex- and mab-3-related transcription factor 2 | Q9Y5R5 | 47 | 6.06 | 2 |
| 567 | Double-stranded RNA-specific adenosine deaminase | P55265 | 149 | 8.59 | 6 |
| 568 | Down syndrome cell adhesion molecule | O60469 | 41 | 1.32 | 2 |
| 569 | Down syndrome cell adhesion molecule-like protein 1 | Q8TD84 | 59 | 1.51 | 4 |
| 570 | Drebrin | Q16643 | 188 | 9.72 | 5 |
| 571 | Drebrin-like protein | Q9UJU6 | 58 | 4.65 | 2 |
| 572 | Dual oxidase 1 | Q9NRD9 | 51 | 1.87 | 2 |
| 573 | Dual specificity mitogen-activated protein kinase kinase 2 | P36507 | 67 | 7.50 | 3 |
| 574 | Dual specificity protein phosphatase 14 | O95147 | 51 | 3.75 | 3 |
| 575 | Dual specificity tyrosine-phosphorylation-regulated kinase 1B | Q9Y463 | 45 | 3.06 | 3 |
| 576 | Dynamin-1 | Q05193 | 148 | 5.09 | 4 |
| 577 | Dynamin-1-like protein | O00429 | 67 | 3.46 | 3 |
| 578 | Dynamin-2 | P50570 | 305 | 9.43 | 9 |
| 579 | Dynein heavy chain 10, axonemal | Q8IVF4 | 69 | 1.28 | 4 |
| 580 | Dynein heavy chain 11, axonemal | Q96DT5 | 58 | 5.23 | 3 |
| 581 | Dynein heavy chain 2, axonemal | Q9P225 | 53 | 0.69 | 3 |
| 582 | Dynein heavy chain 3, axonemal | Q8TD57 | 69 | 1.09 | 4 |
| 583 | Dynein heavy chain 7, axonemal | Q8WXX0 | 66 | 0.62 | 2 |
| 584 | Dynein heavy chain 8, axonemal | Q96JB1 | 46 | 0.53 | 2 |
| 585 | Dynein heavy chain domain-containing protein 1 | Q96M86 | 63 | 2.45 | 3 |
| 586 | Dynein heavy chain, axonemal | Q9P2D7 | 84 | 1.38 | 5 |
| 587 | Dynein intermediate chain 1, cytosolic | O14576 | 44 | 3.50 | 2 |
| 588 | E1A-binding protein p400 | Q96L91 | 51 | 0.82 | 3 |
| 589 | E3 SUMO-protein ligase RanBP2 | P49792 | 68 | 1.74 | 4 |
| 590 | E3 ubiquitin/ISG15 ligase TRIM25 | Q14258 | 50 | 4.50 | 2 |
| 591 | E3 ubiquitin-protein ligase BRE1A | Q5VTR2 | 86 | 5.44 | 5 |
| 592 | E3 ubiquitin-protein ligase BRE1B | O75150 | 80 | 4.60 | 4 |
| 593 | E3 ubiquitin-protein ligase Mdm2 | Q00987 | 64 | 0.86 | 5 |
| 594 | E3 ubiquitin-protein ligase MIB2 | Q96AX9 | 51 | 2.62 | 3 |
| 595 | E3 ubiquitin-protein ligase RNF168 | Q8IYW5 | 51 | 5.62 | 3 |
| 596 | E3 ubiquitin-protein ligase RNF220 | Q5VTB9 | 48 | 4.24 | 2 |
| 597 | E3 ubiquitin-protein ligase UBR4 | Q5T4S7 | 62 | 1.56 | 3 |
| 598 | Early endosome antigen 1 | Q15075 | 176 | 5.32 | 4 |
| 599 | Echinoderm microtubule-associated protein-like 4 | Q9HC35 | 158 | 9.07 | 4 |
| 600 | EF-hand calcium-binding domain-containing protein 6 | Q5THR3 | 43 | 3.13 | 4 |
| 601 | EF-hand domain-containing protein 1 | Q5JVL4 | 41 | 2.97 | 2 |
| 602 | Egl nine homolog 3 | Q9H6Z9 | 50 | 8.48 | 3 |
| 603 | EH domain-binding protein 1 | Q8NDI1 | 40 | 1.72 | 2 |
| 604 | ELAV-like protein 1 | Q15717 | 139 | 9.82 | 4 |
| 605 | Electron transfer flavoprotein subunit beta | P38117 | 389 | 31.89 | 8 |
| 606 | Ellis-van Creveld syndrome protein | P57679 | 45 | 2.31 | 2 |
| 607 | Elongation factor 1-alpha 1 | P68104 | 1059 | 30.74 | 20 |
| 608 | Elongation factor 1-alpha 2 | Q05639 | 69 | 4.32 | 2 |
| 609 | Elongation factor 1-beta | P24534 | 192 | 12.05 | 4 |
| 610 | Elongation factor 1-delta | P29692 | 562 | 17.72 | 14 |
| 611 | Elongation factor 1-gamma | P26641 | 645 | 22.71 | 14 |
| 612 | Elongation factor 2 | P13639 | 1639 | 29.64 | 29 |
| 613 | Elongation factor G, mitochondrial | Q96RP9 | 87 | 3.20 | 3 |
| 614 | EMILIN-1 | Q9Y6C2 | 48 | 2.85 | 2 |
| 615 | Endonuclease VIII-like 1 | Q96FI4 | 42 | 5.14 | 2 |
| 616 | Endonuclease/exonuclease/phosphatase family domain-containing protein 1 | Q7L9B9 | 74 | 5.11 | 4 |
| 617 | Endophilin-A2 | Q99961 | 44 | 4.35 | 2 |
| 618 | Endoplasmic reticulum metallopeptidase 1 | Q7Z2K6 | 45 | 2.95 | 2 |
| 619 | Endoplasmic reticulum protein ERp29 | P30040 | 172 | 16.09 | 4 |
| 620 | Endoplasmin | P14625 | 1510 | 30.05 | 29 |
| 621 | Endothelial differentiation-related factor 1 | O60869 | 136 | 15.54 | 2 |
| 622 | Endothelin-converting enzyme-like 1 | O95672 | 57 | 3.35 | 3 |
| 623 | Enhancer of polycomb homolog 2 | Q52LR7 | 42 | 2.35 | 2 |
| 624 | Enkurin | Q8TC29 | 48 | 7.45 | 2 |
| 625 | Enoyl-CoA hydratase domain-containing protein 1 | Q9NTX5 | 49 | 6.51 | 2 |
| 626 | Enoyl-CoA hydratase domain-containing protein 2, mitochondrial | Q86YB7 | 47 | 7.66 | 2 |
| 627 | Enoyl-CoA hydratase, mitochondrial | P30084 | 243 | 12.41 | 3 |
| 628 | Envoplakin | Q92817 | 82 | 2.80 | 5 |
| 629 | EP300-interacting inhibitor of differentiation 2 | Q8N6I1 | 47 | 15.51 | 2 |
| 630 | Ephrin type-A receptor 4 [Precursor] | P54764 | 55 | 1.64 | 2 |
| 631 | Ephrin type-B receptor 1 | P54762 | 61 | 3.66 | 3 |
| 632 | Ephrin type-B receptor 6 | O15197 | 56 | 3.62 | 3 |
| 633 | Epiplakin | P58107 | 70 | 4.92 | 2 |
| 634 | Erlin-1 | O75477 | 84 | 5.49 | 2 |
| 635 | ERO1-like protein alpha | Q96HE7 | 82 | 6.84 | 3 |
| 636 | ES1 protein homolog, mitochondrial | P30042 | 64 | 5.97 | 2 |
| 637 | Estradiol 17-beta-dehydrogenase 12 | Q53GQ0 | 96 | 7.69 | 3 |
| 638 | Ethanolamine kinase 1 | Q9HBU6 | 50 | 6.86 | 3 |
| 639 | ETS translocation variant 1 | P50549 | 42 | 14.81 | 2 |
| 640 | ETS translocation variant 3 | P41162 | 44 | 3.32 | 2 |
| 641 | Eukaryotic initiation factor 4A-I | P60842 | 619 | 37.19 | 21 |
| 642 | Eukaryotic initiation factor 4A-II | Q14240 | 499 | 30.47 | 15 |
| 643 | Eukaryotic initiation factor 4A-III | P38919 | 458 | 29.61 | 11 |
| 644 | Eukaryotic peptide chain release factor GTP-binding subunit ERF3A | P15170 | 161 | 10.24 | 6 |
| 645 | Eukaryotic translation initiation factor 2 subunit 1 | P05198 | 440 | 24.52 | 11 |
| 646 | Eukaryotic translation initiation factor 2 subunit 2 | P20042 | 100 | 10.51 | 4 |
| 647 | Eukaryotic translation initiation factor 2 subunit 3 | P41091 | 128 | 10.19 | 4 |
| 648 | Eukaryotic translation initiation factor 2A | Q9BY44 | 127 | 6.50 | 3 |
| 649 | Eukaryotic translation initiation factor 3 subunit A | Q14152 | 840 | 20.12 | 18 |
| 650 | Eukaryotic translation initiation factor 3 subunit B | P55884 | 279 | 8.97 | 7 |
| 651 | Eukaryotic translation initiation factor 3 subunit C | Q99613 | 459 | 12.81 | 13 |
| 652 | Eukaryotic translation initiation factor 3 subunit E | P60228 | 182 | 14.16 | 7 |
| 653 | Eukaryotic translation initiation factor 3 subunit F | O00303 | 161 | 21.29 | 6 |
| 654 | Eukaryotic translation initiation factor 3 subunit G | O75821 | 42 | 5.00 | 2 |
| 655 | Eukaryotic translation initiation factor 3 subunit H | O15372 | 131 | 15.47 | 4 |
| 656 | Eukaryotic translation initiation factor 3 subunit I | Q13347 | 255 | 16.62 | 5 |
| 657 | Eukaryotic translation initiation factor 3 subunit L | Q9Y262 | 201 | 9.57 | 6 |
| 658 | Eukaryotic translation initiation factor 4 gamma 1 | Q04637 | 818 | 15.50 | 18 |
| 659 | Eukaryotic translation initiation factor 4 gamma, 2 | P78344 | 72 | 3.11 | 3 |
| 660 | Eukaryotic translation initiation factor 4B | P23588 | 135 | 8.51 | 5 |
| 661 | Eukaryotic translation initiation factor 4E | P06730 | 130 | 10.14 | 2 |
| 662 | Eukaryotic translation initiation factor 4H | Q15056 | 99 | 13.91 | 3 |
| 663 | Eukaryotic translation initiation factor 5A-1 | P63241 | 145 | 12.99 | 2 |
| 664 | Eukaryotic translation initiation factor 6 | P56537 | 84 | 10.19 | 2 |
| 665 | Exocyst complex component 4 | Q96A65 | 52 | 1.33 | 2 |
| 666 | Exophilin-5 | Q8NEV8 | 62 | 1.66 | 3 |
| 667 | Exosome complex exonuclease RRP41 | Q9NPD3 | 46 | 7.38 | 2 |
| 668 | Exosome complex exonuclease RRP44 | Q9Y2L1 | 152 | 6.70 | 6 |
| 669 | Exportin-1 | O14980 | 280 | 6.72 | 6 |
| 670 | Extended-synaptotagmin-1 | Q9BSJ8 | 144 | 5.53 | 4 |
| 671 | Extracellular calcium-sensing receptor | P41180 | 43 | 3.25 | 2 |
| 672 | Ezrin | P15311 | 337 | 11.28 | 10 |
| 673 | FACT complex subunit SPT16 | Q9Y5B9 | 125 | 6.88 | 4 |
| 674 | FACT complex subunit SSRP1 | Q08945 | 132 | 6.06 | 4 |
| 675 | F-actin-capping protein subunit alpha-2 | P47755 | 62 | 6.32 | 2 |
| 676 | F-actin-capping protein subunit beta | P47756 | 175 | 20.59 | 6 |
| 677 | Fanconi anemia group M protein | Q8IYD8 | 45 | 2.84 | 2 |
| 678 | Far upstream element-binding protein 1 | Q96AE4 | 178 | 8.55 | 5 |
| 679 | Far upstream element-binding protein 2 | Q92945 | 796 | 26.06 | 17 |
| 680 | Farnesyl pyrophosphate synthetase | P14324 | 386 | 24.36 | 9 |
| 681 | FAS-associated factor 1 | Q9UNN5 | 61 | 4.96 | 3 |
| 682 | Fascin | Q16658 | 233 | 14.63 | 7 |
| 683 | FAST kinase domain-containing protein 5 | Q7L8L6 | 47 | 2.72 | 2 |
| 684 | Fatty acid synthase | P49327 | 3693 | 31.04 | 63 |
| 685 | Fatty acid-binding protein, liver | P07148 | 221 | 33.86 | 4 |
| 686 | F-box only protein 38 | Q6PIJ6 | 46 | 2.86 | 2 |
| 687 | F-box only protein 41 | Q8TF61 | 50 | 5.03 | 3 |
| 688 | F-box/LRR-repeat protein 17 | Q9UF56 | 42 | 4.07 | 2 |
| 689 | F-box/LRR-repeat protein 19 | Q6PCT2 | 59 | 4.30 | 3 |
| 690 | F-box/LRR-repeat protein 8 | Q96CD0 | 48 | 13.10 | 3 |
| 691 | F-box/WD repeat-containing protein 10 | Q5XX13 | 53 | 8.88 | 2 |
| 692 | FERM domain-containing protein 1 | Q8N878 | 41 | 3.28 | 2 |
| 693 | FERM domain-containing protein 4B | Q9Y2L6 | 54 | 4.03 | 2 |
| 694 | FH1/FH2 domain-containing protein 1 | Q9Y613 | 66 | 1.89 | 3 |
| 695 | Fibrillin-1 | P35555 | 41 | 1.76 | 2 |
| 696 | Fibrillin-3 | Q75N90 | 41 | 0.96 | 2 |
| 697 | Fibroblast growth factor receptor 2 | P21802 | 41 | 2.55 | 2 |
| 698 | Fibroblast growth factor receptor 3 | P22607 | 69 | 2.20 | 2 |
| 699 | Fibrocystin-L | Q86WI1 | 62 | 0.90 | 3 |
| 700 | Fibronectin | P02751 | 196 | 4.82 | 5 |
| 701 | Fibronectin type III and SPRY domain-containing protein 1 | Q9BTV5 | 54 | 5.04 | 2 |
| 702 | Fibronectin type III domain-containing protein 1 | Q4ZHG4 | 47 | 1.03 | 2 |
| 703 | Fibrous sheath-interacting protein 2 | Q5CZC0 | 46 | 2.27 | 2 |
| 704 | Filaggrin | P20930 | 44 | 1.36 | 2 |
| 705 | Filamin A | P21333 | 832 | 11.45 | 20 |
| 706 | Filamin B | O75369 | 1380 | 20.92 | 37 |
| 707 | Filamin-A | P21333 | 562 | 8.35 | 19 |
| 708 | FK506-binding protein 11 | Q9NYL4 | 94 | 14.93 | 2 |
| 709 | Flavin reductase | P30043 | 95 | 20.49 | 3 |
| 710 | FMRFamide-related peptides | Q9HCQ7 | 48 | 15.31 | 2 |
| 711 | Folliculin-interacting protein 1 | Q8TF40 | 52 | 1.25 | 2 |
| 712 | Forkhead box protein K1 | P85037 | 52 | 1.25 | 2 |
| 713 | Forkhead-associated domain-containing protein 1 | B1AJZ9 | 49 | 6.82 | 2 |
| 714 | Formin-2 | Q9NZ56 | 47 | 1.23 | 3 |
| 715 | Formin-binding protein 4 | Q8N3X1 | 71 | 3.24 | 3 |
| 716 | Fragile X mental retardation syndrome-related protein 1 | P51114 | 53 | 4.35 | 3 |
| 717 | FRAS1-related extracellular matrix protein 2 | Q5SZK8 | 51 | 1.33 | 3 |
| 718 | FRAS1-related extracellular matrix protein 3 | P0C091 | 56 | 1.78 | 3 |
| 719 | Fructose-bisphosphate aldolase A | P04075 | 1513 | 53.30 | 27 |
| 720 | Fructose-bisphosphate aldolase C | P09972 | 521 | 38.46 | 12 |
| 721 | Fumarate hydratase, mitochondrial | P07954 | 110 | 5.69 | 2 |
| 722 | FYVE, RhoGEF and PH domain-containing protein 5 | Q6ZNL6 | 46 | 1.30 | 3 |
| 723 | FYVE, RhoGEF and PH domain-containing protein 6 | Q6ZV73 | 42 | 1.49 | 2 |
| 724 | G patch domain-containing protein 8 | Q9UKJ3 | 49 | 1.24 | 2 |
| 725 | G protein pathway suppressor 2 | Q13227 | 46 | 5.20 | 2 |
| 726 | G protein-regulated inducer of neurite outgrowth 1 | Q7Z2K8 | 80 | 5.25 | 4 |
| 727 | Galactokinase | P51570 | 440 | 35.20 | 14 |
| 728 | Galactosylgalactosylxylosylprotein 3-beta-glucuronosyltransferase 1 | Q9P2W7 | 54 | 5.69 | 2 |
| 729 | Galectin-3 | P17931 | 439 | 51.82 | 6 |
| 730 | Galectin-8 | O00214 | 45 | 9.91 | 2 |
| 731 | Gamma-aminobutyric acid type B receptor subunit 2 | O75899 | 48 | 2.27 | 2 |
| 732 | Gamma-enolase | P09104 | 221 | 12.24 | 4 |
| 733 | Gamma-glutamyl hydrolase | Q92820 | 114 | 11.01 | 3 |
| 734 | Gamma-soluble NSF attachment protein | Q99747 | 137 | 20.51 | 6 |
| 735 | Gamma-tubulin complex component 6 | Q96RT7 | 44 | 1.49 | 2 |
| 736 | Gasdermin-C | Q9BYG8 | 43 | 4.33 | 2 |
| 737 | GDP-fucose protein O-fucosyltransferase 1 | Q9H488 | 55 | 14.43 | 2 |
| 738 | GDP-fucose protein O-fucosyltransferase 2 | Q9Y2G5 | 42 | 16.31 | 2 |
| 739 | Gelsolin | P06396 | 118 | 11.15 | 3 |
| 740 | Gem-associated protein 5 | Q8TEQ6 | 108 | 3.98 | 3 |
| 741 | GEM-interacting protein | Q9P107 | 40 | 1.86 | 2 |
| 742 | General transcription factor 3C polypeptide 1 | Q12789 | 341 | 7.53 | 7 |
| 743 | General transcription factor 3C polypeptide 3 | Q9Y5Q9 | 47 | 1.47 | 2 |
| 744 | General transcription factor II-I | P78347 | 254 | 12.33 | 7 |
| 745 | Gigaxonin | Q9H2C0 | 64 | 3.35 | 2 |
| 746 | Girdin | Q3V6T2 | 63 | 1.71 | 3 |
| 747 | Glial fibrillary acidic protein | P14136 | 291 | 12.04 | 7 |
| 748 | Glioma tumor suppressor candidate region gene 2 protein | Q9NZM5 | 53 | 3.59 | 2 |
| 749 | Gliomedin | Q6ZMI3 | 41 | 5.44 | 2 |
| 750 | Glucosamine--fructose-6-phosphate aminotransferase [isomerizing] 1 | Q06210 | 93 | 5.87 | 5 |
| 751 | Glucose 1,6-bisphosphate synthase | Q6PCE3 | 52 | 4.34 | 2 |
| 752 | Glucose-6-phosphate isomerase | P06744 | 133 | 8.26 | 5 |
| 753 | Glucosidase 2 subunit beta | P14314 | 178 | 12.06 | 6 |
| 754 | Glutamate [NMDA] receptor subunit zeta-1 | Q05586 | 52 | 6.18 | 3 |
| 755 | Glutamate decarboxylase 1 | Q99259 | 44 | 2.86 | 2 |
| 756 | Glutamate dehydrogenase 1, mitochondrial | P00367 | 715 | 27.96 | 13 |
| 757 | Glutamate receptor-interacting protein 1 | Q9Y3R0 | 48 | 1.68 | 2 |
| 758 | Glutaminase kidney isoform, mitochondrial | O94925 | 106 | 1.84 | 3 |
| 759 | Glutaminase liver isoform, mitochondrial | Q9UI32 | 51 | 3.65 | 2 |
| 760 | Glutamine and serine-rich protein 1 | Q2KHR3 | 45 | 1.12 | 2 |
| 761 | Glutamine synthetase | P15104 | 74 | 9.14 | 4 |
| 762 | Glutaminyl-tRNA synthetase | P47897 | 230 | 10.19 | 10 |
| 763 | Glutaredoxin-3 | O76003 | 358 | 31.14 | 8 |
| 764 | Glutathione reductase | P00390 | 114 | 7.16 | 3 |
| 765 | Glutathione S-transferase A1 | P08263 | 502 | 29.41 | 11 |
| 766 | Glutathione S-transferase A2 | P09210 | 500 | 29.41 | 11 |
| 767 | Glutathione S-transferase A3 | Q16772 | 218 | 16.42 | 6 |
| 768 | Glutathione S-transferase P | P09211 | 563 | 41.15 | 8 |
| 769 | Glutathione synthetase | P48637 | 146 | 9.75 | 5 |
| 770 | Glyceraldehyde-3-phosphate dehydrogenase | P04406 | 433 | 21.79 | 9 |
| 771 | Glycerol-3-phosphate acyltransferase 2, mitochondrial | Q6NUI2 | 49 | 4.29 | 2 |
| 772 | Glycerophosphodiester phosphodiesterase 1 | Q9NZC3 | 46 | 7.25 | 2 |
| 773 | Glycine dehydrogenase | P23378 | 673 | 20.20 | 17 |
| 774 | Glycogen debranching enzyme | P35573 | 402 | 10.84 | 12 |
| 775 | Glycogen phosphorylase | P06737 | 273 | 8.27 | 7 |
| 776 | Glycolipid transfer protein | Q9NZD2 | 107 | 11.65 | 5 |
| 777 | Glycylpeptide N-tetradecanoyltransferase 1 | P30419 | 73 | 5.67 | 3 |
| 778 | Glycyl-tRNA synthetase | P41250 | 80 | 5.06 | 3 |
| 779 | Glyoxalase domain-containing protein 4 | Q9HC38 | 62 | 9.40 | 2 |
| 780 | Glyoxylate reductase/hydroxypyruvate reductase | Q9UBQ7 | 50 | 3.23 | 3 |
| 781 | GMP synthase [glutamine-hydrolyzing] | P49915 | 63 | 4.18 | 4 |
| 782 | Golgi apparatus protein 1 | Q92896 | 200 | 5.40 | 4 |
| 783 | Golgin subfamily A member 3 | Q08378 | 91 | 2.94 | 3 |
| 784 | Golgin subfamily A member 4 | Q13439 | 50 | 0.76 | 2 |
| 785 | Golgin subfamily B member 1 | Q14789 | 48 | 0.74 | 2 |
| 786 | G-protein coupled receptor 126 | Q86SQ4 | 47 | 1.80 | 2 |
| 787 | GRIP and coiled-coil domain-containing protein 2 | Q8IWJ2 | 52 | 1.71 | 2 |
| 788 | GRIP1-associated protein 1 | Q4V328 | 46 | 1.31 | 2 |
| 789 | GrpE protein homolog 1, mitochondrial | Q9HAV7 | 122 | 10.19 | 3 |
| 790 | GTP:AMP phosphotransferase mitochondrial | Q9UIJ7 | 224 | 19.28 | 4 |
| 791 | GTPase-activating protein and VPS9 domain-containing protein 1 | Q14C86 | 120 | 5.19 | 3 |
| 792 | GTP-binding nuclear protein Ran | P62825 | 484 | 26.98 | 9 |
| 793 | GTP-binding protein GUF1 homolog | Q8N442 | 60 | 3.10 | 2 |
| 794 | GTP-binding protein Rheb | Q15382 | 49 | 9.78 | 3 |
| 795 | Guanine nucleotide-binding protein G(i), alpha-2 subunit | P04899 | 118 | 11.02 | 3 |
| 796 | Guanine nucleotide-binding protein G(k) subunit alpha | P08754 | 159 | 13.88 | 4 |
| 797 | Guanine nucleotide-binding protein G(o) subunit alpha | P09471 | 53 | 5.38 | 2 |
| 798 | Guanine nucleotide-binding protein G(s) subunit alpha isoforms XLas | Q5JWF2 | 72 | 9.74 | 3 |
| 799 | Guanine nucleotide-binding protein-like 1 | P36915 | 45 | 3.46 | 2 |
| 800 | Guanylate-binding protein 4 | Q96PP9 | 72 | 4.38 | 3 |
| 801 | Hairy/enhancer-of-split related with YRPW motif protein 1 | Q9Y5J3 | 53 | 6.68 | 2 |
| 802 | Hairy/enhancer-of-split related with YRPW motif protein 2 | Q9UBP5 | 43 | 9.50 | 3 |
| 803 | HAUS augmin-like complex subunit 3 | Q68CZ6 | 45 | 2.82 | 2 |
| 804 | HAUS augmin-like complex subunit 4 | Q9H6D7 | 42 | 4.68 | 2 |
| 805 | HAUS augmin-like complex subunit 5 | O94927 | 58 | 2.84 | 2 |
| 806 | HEAT repeat-containing protein C7orf27 | Q6PJG6 | 41 | 3.41 | 2 |
| 807 | HEAT repeat-containing protein KIAA1833 | Q8NDA8 | 58 | 1.13 | 2 |
| 808 | Heat shock 70 kDa protein 1 | P08107 | 896 | 27.77 | 18 |
| 809 | Heat shock 70 kDa protein 1A/1B | P08107 | 50 | 10.00 | 3 |
| 810 | Heat shock 70 kDa protein 1-like | P34931 | 511 | 15.44 | 10 |
| 811 | Heat shock 70 kDa protein 9 | P38646 | 542 | 24.45 | 14 |
| 812 | Heat shock 70kDa protein 4 | P34932 | 1551 | 36.19 | 32 |
| 813 | Heat shock 70kDa protein 6 | P17066 | 500 | 16.49 | 10 |
| 814 | Heat shock 90 kDa protein B1 | P14625 | 134 | 13.72 | 4 |
| 815 | Heat shock cognate 71 kDa protein | P11142 | 1358 | 38.24 | 25 |
| 816 | Heat shock protein 105 kDa | Q92598 | 943 | 21.54 | 21 |
| 817 | Heat shock protein 60 kDa | P10809 | 1323 | 31.13 | 20 |
| 818 | Heat shock protein 75 kDa, mitochondrial | Q12931 | 149 | 3.97 | 3 |
| 819 | Heat shock protein 90-beta | P08238 | 2486 | 44.54 | 43 |
| 820 | Heat shock protein HSP 90-alpha | P07900 | 2103 | 37.98 | 35 |
| 821 | Hedgehog-interacting protein | Q96QV1 | 50 | 3.43 | 2 |
| 822 | Helicase POLQ-like | Q8TDG4 | 55 | 1.02 | 2 |
| 823 | Helicase SRCAP | Q6ZRS2 | 46 | 1.00 | 2 |
| 824 | Hematological and neurological expressed 1-like protein | Q9H910 | 84 | 16.67 | 2 |
| 825 | Hemicentin-2 | Q8NDA2 | 51 | 1.64 | 2 |
| 826 | Heparin-binding growth factor 2 | P09038 | 43 | 10.00 | 2 |
| 827 | Hepatoma-derived growth factor | P51858 | 145 | 16.67 | 5 |
| 828 | Hepatoma-derived growth factor 2 | Q7Z4V5 | 70 | 4.02 | 2 |
| 829 | Hermansky-Pudlak syndrome 4 protein | Q9NQG7 | 65 | 4.98 | 3 |
| 830 | Hermansky-Pudlak syndrome 6 protein | Q86YV9 | 42 | 2.71 | 2 |
| 831 | Heterogeneous nuclear ribonucleoprotein A/B | Q99729 | 364 | 25.00 | 10 |
| 832 | Heterogeneous nuclear ribonucleoprotein A1 | P09651 | 465 | 26.25 | 7 |
| 833 | Heterogeneous nuclear ribonucleoprotein A3 | P51991 | 802 | 42.06 | 15 |
| 834 | Heterogeneous nuclear ribonucleoprotein C-like 1 | O60812 | 306 | 18.36 | 6 |
| 835 | Heterogeneous nuclear ribonucleoprotein D0 | Q14103 | 199 | 16.62 | 5 |
| 836 | Heterogeneous nuclear ribonucleoprotein D-like | O14979 | 153 | 15.77 | 5 |
| 837 | Heterogeneous nuclear ribonucleoprotein F | P52597 | 376 | 20.77 | 7 |
| 838 | Heterogeneous nuclear ribonucleoprotein G | P38159 | 377 | 21.74 | 10 |
| 839 | Heterogeneous nuclear ribonucleoprotein H | P31943 | 429 | 13.39 | 7 |
| 840 | Heterogeneous nuclear ribonucleoprotein H2 | P55795 | 285 | 9.58 | 6 |
| 841 | Heterogeneous nuclear ribonucleoprotein H3 | P31942 | 353 | 24.86 | 8 |
| 842 | Heterogeneous nuclear ribonucleoprotein K | P61978 | 570 | 34.05 | 16 |
| 843 | Heterogeneous nuclear ribonucleoprotein L | P14866 | 393 | 15.77 | 10 |
| 844 | Heterogeneous nuclear ribonucleoprotein M | P52272 | 124 | 7.41 | 4 |
| 845 | Heterogeneous nuclear ribonucleoprotein Q | O60506 | 152 | 6.58 | 4 |
| 846 | Heterogeneous nuclear ribonucleoprotein R | O43390 | 67 | 4.24 | 2 |
| 847 | Heterogeneous nuclear ribonucleoprotein U | Q00839 | 684 | 17.13 | 13 |
| 848 | Heterogeneous nuclear ribonucleoprotein U-like protein 1 | Q9BUJ2 | 74 | 2.80 | 2 |
| 849 | Heterogeneous nuclear ribonucleoproteins A2/B1 | P22626 | 1238 | 58.64 | 21 |
| 850 | Heterogeneous nuclear ribonucleoproteins C1/C2 | P07910 | 565 | 23.86 | 12 |
| 851 | Hexokinase-1 | P19367 | 41 | 2.45 | 2 |
| 852 | Hexokinase-2 | P52789 | 61 | 5.23 | 4 |
| 853 | High affinity cAMP-specific and IBMX-insensitive 3',5'-cyclic phosphodiesterase 8B | O95263 | 57 | 4.10 | 2 |
| 854 | High affinity nerve growth factor receptor | P04629 | 637 | 22.31 | 16 |
| 855 | High mobility group protein B1 | P09429 | 78 | 7.94 | 2 |
| 856 | High mobility group protein B2 | P26583 | 171 | 11.54 | 3 |
| 857 | Histidine triad nucleotide-binding protein 1 | P49773 | 175 | 36.00 | 3 |
| 858 | Histidyl-tRNA synthetase, cytoplasmic | P12081 | 86 | 5.11 | 3 |
| 859 | Histone acetyltransferase KAT2A | Q92830 | 56 | 4.30 | 3 |
| 860 | Histone acetyltransferase type B catalytic subunit | O14929 | 71 | 4.49 | 2 |
| 861 | Histone demethylase JARID1C | P41229 | 45 | 1.16 | 2 |
| 862 | Histone H1.0 | P07305 | 77 | 17.62 | 3 |
| 863 | Histone H1.2 | P16403 | 647 | 37.56 | 9 |
| 864 | Histone H1.4 | P10412 | 656 | 36.53 | 9 |
| 865 | Histone H1.5 | P16401 | 625 | 35.56 | 10 |
| 866 | Histone H1t | P22492 | 221 | 13.11 | 3 |
| 867 | Histone H1x | Q92522 | 356 | 27.70 | 7 |
| 868 | Histone H2A type 1 | P0C0S8 | 373 | 35.38 | 5 |
| 869 | Histone H2A type 1-B | P04908 | 383 | 35.66 | 5 |
| 870 | Histone H2A.J | Q9BTM1 | 181 | 25.17 | 5 |
| 871 | Histone H2A.V | Q71UI9 | 262 | 31.50 | 4 |
| 872 | Histone H2A.x | P16104 | 53 | 16.20 | 2 |
| 873 | Histone H2B type 1-A | Q96A08 | 355 | 39.68 | 6 |
| 874 | Histone H2B type 1-B | P33778 | 495 | 57.60 | 9 |
| 875 | Histone H2B type 1-C/E/F/G/I | P62807 | 561 | 58.87 | 10 |
| 876 | Histone H3.1 | P68431 | 431 | 45.19 | 9 |
| 877 | Histone H3.1t | Q16695 | 294 | 27.41 | 8 |
| 878 | Histone H3.3 | P84243 | 368 | 45.19 | 9 |
| 879 | Histone H4 | P62805 | 921 | 61.17 | 16 |
| 880 | Histone-lysine N-methyltransferase MLL | Q03164 | 92 | 1.13 | 5 |
| 881 | Histone-lysine N-methyltransferase MLL3 | Q8NEZ4 | 64 | 0.61 | 3 |
| 882 | Histone-lysine N-methyltransferase MLL4 | Q9UMN6 | 45 | 1.40 | 3 |
| 883 | Histone-lysine N-methyltransferase MLL5 | Q8IZD2 | 55 | 7.95 | 2 |
| 884 | Histone-lysine N-methyltransferase SUV420H1 | Q4FZB7 | 41 | 2.03 | 2 |
| 885 | Homeobox protein C14 | Q7Z5D8 | 57 | 15.69 | 2 |
| 886 | Homeobox protein cut-like 2 | O14529 | 57 | 2.79 | 3 |
| 887 | Homeobox protein DLX-4 | Q92988 | 47 | 8.07 | 3 |
| 888 | Homeobox protein GBX-2 | P52951 | 58 | 19.55 | 3 |
| 889 | Hsc70-interacting protein | P50502 | 205 | 16.08 | 4 |
| 890 | Hsp70-binding protein 1 | Q9NZL4 | 50 | 5.85 | 2 |
| 891 | Huntingtin-interacting protein 1-related protein | O75146 | 52 | 4.96 | 3 |
| 892 | Hyaluronan mediated motility receptor | O75330 | 59 | 4.83 | 3 |
| 893 | Hydroxymethylglutaryl-CoA synthase, mitochondrial | P54868 | 145 | 9.45 | 6 |
| 894 | Hypoxia up-regulated protein 1 | Q9Y4L1 | 476 | 14.31 | 8 |
| 895 | Ig heavy chain V-I region HG3 | P01743 | 47 | 14.38 | 2 |
| 896 | Ig heavy chain V-III region GAL | P01781 | 55 | 16.22 | 2 |
| 897 | IgGFc-binding protein | Q9Y6R7 | 86 | 1.18 | 5 |
| 898 | Immunoglobulin superfamily DCC subclass member 4 | Q8TDY8 | 52 | 3.37 | 2 |
| 899 | Immunoglobulin superfamily member 3 | O75054 | 95 | 4.02 | 3 |
| 900 | Importin 7 | O95373 | 223 | 5.68 | 6 |
| 901 | Importin subunit alpha-2 | P52292 | 142 | 8.32 | 3 |
| 902 | Importin subunit beta-1 | Q14974 | 409 | 9.87 | 7 |
| 903 | Importin-5 | O00410 | 270 | 11.58 | 10 |
| 904 | Inactive serine protease RAMP | Q6UXH9 | 42 | 2.58 | 2 |
| 905 | Indian hedgehog protein | Q14623 | 52 | 7.30 | 2 |
| 906 | INO80 complex subunit D | Q53TQ3 | 53 | 5.27 | 2 |
| 907 | Inorganic pyrophosphatase | Q15181 | 57 | 6.23 | 2 |
| 908 | Inosine-5'-monophosphate dehydrogenase 2 | P12268 | 120 | 6.23 | 3 |
| 909 | Inositol 1,4,5-trisphosphate receptor type 1 | Q14643 | 74 | 1.96 | 6 |
| 910 | Inositol 1,4,5-trisphosphate receptor type 3 | Q14573 | 95 | 3.03 | 3 |
| 911 | Inositol polyphosphate 5-phosphatase OCRL-1 | Q01968 | 45 | 2.06 | 2 |
| 912 | Inositol-trisphosphate 3-kinase B | P27987 | 46 | 2.22 | 2 |
| 913 | Insulin-like growth factor 2 mRNA-binding protein 1 | Q9NZI8 | 1081 | 36.22 | 22 |
| 914 | Insulin-like growth factor 2 mRNA-binding protein 2 | Q9Y6M1 | 603 | 25.08 | 14 |
| 915 | Insulin-like growth factor 2 mRNA-binding protein 3 | O00425 | 189 | 7.43 | 5 |
| 916 | Integrin alpha-4 | P13612 | 58 | 5.35 | 2 |
| 917 | Integrin alpha-6 | P23229 | 56 | 2.92 | 3 |
| 918 | Integrin alpha-L | P20701 | 42 | 1.84 | 2 |
| 919 | Interferon alpha-1/13 | P01562 | 58 | 19.58 | 3 |
| 920 | Interferon-induced GTP-binding protein Mx2 | P20592 | 67 | 8.78 | 2 |
| 921 | Interferon-induced guanylate-binding protein 1 | P32455 | 48 | 5.07 | 3 |
| 922 | Interleukin enhancer-binding factor 2 | Q12905 | 237 | 19.49 | 8 |
| 923 | Interleukin enhancer-binding factor 3 | Q12906 | 414 | 12.98 | 13 |
| 924 | Interleukin-31 receptor subunit alpha | Q8NI17 | 42 | 3.70 | 2 |
| 925 | Intermediate conductance calcium-activated potassium channel protein 4 | O15554 | 53 | 7.73 | 2 |
| 926 | Intraflagellar transport protein 172 homolog | Q9UG01 | 44 | 2.48 | 2 |
| 927 | Inversin | Q9Y283 | 96 | 4.88 | 5 |
| 928 | IQ domain-containing protein G | Q9H095 | 60 | 4.30 | 2 |
| 929 | IQ motif and SEC7 domain-containing protein 3 | Q9UPP2 | 40 | 1.95 | 2 |
| 930 | Isocitrate dehydrogenase [NAD] subunit alpha, mitochondrial | P50213 | 51 | 6.60 | 2 |
| 931 | Isocitrate dehydrogenase [NADP] cytoplasmic | O75874 | 43 | 17.53 | 3 |
| 932 | Isocitrate dehydrogenase [NADP], mitochondrial | P48735 | 372 | 17.39 | 6 |
| 933 | JmjC domain-containing protein 8 | Q96S16 | 40 | 5.69 | 2 |
| 934 | Junction plakoglobin | P14923 | 188 | 8.72 | 6 |
| 935 | Junctional adhesion molecule A | Q9Y624 | 110 | 17.37 | 3 |
| 936 | Junctophilin-3 | Q8WXH2 | 49 | 2.67 | 2 |
| 937 | Keratin 10, type I, cytoskeletal | P13645 | 86 | 8.09 | 2 |
| 938 | Keratin, type I cytoskeletal 12 | Q99456 | 103 | 5.26 | 3 |
| 939 | Keratin, type I cytoskeletal 13 | P13646 | 142 | 12.23 | 6 |
| 940 | Keratin, type I cytoskeletal 14 | P02533 | 263 | 15.68 | 9 |
| 941 | Keratin, type I cytoskeletal 15 | P19012 | 170 | 8.11 | 5 |
| 942 | Keratin, type I cytoskeletal 17 | Q04695 | 309 | 15.55 | 9 |
| 943 | Keratin, type I cytoskeletal 18 | P05783 | 2152 | 65.97 | 42 |
| 944 | Keratin, type I cytoskeletal 19 | P08727 | 1931 | 82.75 | 38 |
| 945 | Keratin, type II cytoskeletal 1 | P04264 | 118 | 6.52 | 4 |
| 946 | Keratin, type II cytoskeletal 3 | P12035 | 130 | 7.15 | 4 |
| 947 | Keratin, type II cytoskeletal 4 | P19013 | 99 | 7.10 | 3 |
| 948 | Keratin, type II cytoskeletal 5 | P13647 | 86 | 4.58 | 3 |
| 949 | Keratin, type II cytoskeletal 6A | P02538 | 49 | 4.79 | 3 |
| 950 | Keratin, type II cytoskeletal 6C | P48668 | 72 | 6.03 | 3 |
| 951 | Keratin, type II cytoskeletal 7 | P08729 | 146 | 8.33 | 5 |
| 952 | Keratin, type II cytoskeletal 78 | Q8N1N4 | 243 | 4.03 | 4 |
| 953 | Keratin, type II cytoskeletal 79 | Q5XKE5 | 283 | 6.54 | 7 |
| 954 | Keratin, type II cytoskeletal 8 | P05787 | 2599 | 59.34 | 59 |
| 955 | Ketosamine-3-kinase | Q9HA64 | 58 | 6.80 | 2 |
| 956 | KH domain-containing, RNA-binding, signal transduction-associated protein 1 (p68) | Q07666 | 198 | 13.09 | 7 |
| 957 | Kin of IRRE-like protein 3 | Q8IZU9 | 59 | 2.19 | 2 |
| 958 | Kinesin heavy chain isoform 5A | Q12840 | 53 | 2.88 | 3 |
| 959 | Kinesin-1 heavy chain | P33176 | 184 | 9.35 | 8 |
| 960 | Kinesin-like protein KIF13A | Q9H1H9 | 67 | 1.81 | 4 |
| 961 | Kinesin-like protein KIF13B | Q9NQT8 | 65 | 1.91 | 4 |
| 962 | Kinesin-like protein KIF15 | Q9NS87 | 64 | 3.53 | 4 |
| 963 | Kinesin-like protein KIF17 | Q9P2E2 | 50 | 3.11 | 3 |
| 964 | Kinesin-like protein KIF1A | Q12756 | 79 | 2.72 | 5 |
| 965 | Kinesin-like protein KIF1B | O60333 | 58 | 1.54 | 4 |
| 966 | Kinesin-like protein KIF1C | O43896 | 57 | 3.63 | 3 |
| 967 | Kinesin-like protein KIF21A | Q7Z4S6 | 51 | 6.79 | 2 |
| 968 | Kinesin-like protein KIF21B | O75037 | 44 | 1.10 | 2 |
| 969 | Kinesin-like protein KIF22 | Q14807 | 55 | 4.96 | 2 |
| 970 | Kinesin-like protein KIF26A | Q9ULI4 | 90 | 2.83 | 5 |
| 971 | Kinesin-like protein KIF27 | Q86VH2 | 58 | 2.76 | 3 |
| 972 | Kinesin-like protein KIF2C | Q99661 | 108 | 7.03 | 5 |
| 973 | Kinesin-like protein KIF3B | O15066 | 43 | 2.95 | 2 |
| 974 | Kinesin-like protein KIF7 | Q2M1P5 | 65 | 3.10 | 4 |
| 975 | Kinetochore protein NDC80 homolog | O14777 | 73 | 6.07 | 4 |
| 976 | KN motif and ankyrin repeat domain-containing protein 2 | Q63ZY3 | 43 | 2.63 | 2 |
| 977 | Lactase-like protein | Q6UWM7 | 52 | 4.94 | 3 |
| 978 | Lactotransferrin | P02788 | 63 | 2.53 | 2 |
| 979 | Lactoylglutathione lyase | Q04760 | 118 | 8.74 | 3 |
| 980 | Lamin-A/C ( Renal carcinoma antigen NY-REN-32) | P02545 | 312 | 16.42 | 10 |
| 981 | Lamina-associated polypeptide 2, isoforms beta/gamma | P42167 | 117 | 11.26 | 4 |
| 982 | Lamin-B1 | P20700 | 282 | 12.97 | 10 |
| 983 | Lamin-B2 | Q03252 | 272 | 16.50 | 11 |
| 984 | Laminin subunit alpha-1 | P25391 | 70 | 1.20 | 4 |
| 985 | Laminin subunit alpha-2 | P24043 | 46 | 1.43 | 2 |
| 986 | Laminin subunit alpha-3 | Q16787 | 53 | 0.64 | 2 |
| 987 | Laminin subunit alpha-5 | O15230 | 59 | 0.51 | 2 |
| 988 | Laminin subunit beta-1 | P07942 | 133 | 3.14 | 5 |
| 989 | Laminin subunit beta-2 | P55268 | 52 | 5.51 | 2 |
| 990 | Laminin subunit gamma-1 | P11047 | 186 | 3.73 | 4 |
| 991 | Laminin subunit gamma-2 | Q13753 | 40 | 2.25 | 2 |
| 992 | L-aminoadipate-semialdehyde dehydrogenase-phosphopantetheinyl transferase | Q9NRN7 | 163 | 12.61 | 4 |
| 993 | LanC-like protein 1 | O43813 | 94 | 7.02 | 3 |
| 994 | La-related protein 1 | Q6PKG0 | 260 | 10.79 | 8 |
| 995 | Large neutral amino acids transporter small subunit 1 | Q01650 | 100 | 6.31 | 2 |
| 996 | Large neutral amino acids transporter small subunit 4 | Q8N370 | 89 | 6.95 | 3 |
| 997 | Large proline-rich protein BAT3 | P46379 | 511 | 11.63 | 9 |
| 998 | LEM domain-containing protein 2 | Q8NC56 | 55 | 8.55 | 4 |
| 999 | Leucine zipper-EF-hand-containing transmembrane protein 1 | O95202 | 118 | 7.44 | 5 |
| 1000 | Leucine-rich PPR motif-containing protein | P42704 | 816 | 20.01 | 17 |
| 1001 | Leucine-rich repeat and fibronectin type-III domain-containing protein 2 | Q9ULH4 | 41 | 17.86 | 2 |
| 1002 | Leucine-rich repeat and WD repeat-containing protein KIAA1239 | Q9ULI1 | 42 | 1.46 | 2 |
| 1003 | Leucine-rich repeat serine/threonine-protein kinase 2 | Q5S007 | 45 | 0.83 | 2 |
| 1004 | Leucine-rich repeat transmembrane protein FLRT1 | Q9NZU1 | 55 | 3.56 | 2 |
| 1005 | Leucine-rich repeat transmembrane protein FLRT3 | Q9NZU0 | 50 | 3.83 | 2 |
| 1006 | Leucine-rich repeat-containing protein 16C | Q6F5E8 | 74 | 2.31 | 4 |
| 1007 | Leucine-rich repeat-containing protein 47 | Q8N1G4 | 153 | 9.09 | 6 |
| 1008 | Leucine-rich repeat-containing protein 59 | Q96AG4 | 73 | 3.91 | 2 |
| 1009 | Leucine-rich repeat-containing protein 6 | Q86X45 | 40 | 7.06 | 2 |
| 1010 | Leukocyte elastase inhibitor | P30740 | 231 | 18.47 | 6 |
| 1011 | LIM and calponin homology domains-containing protein 1 | Q9UPQ0 | 51 | 1.36 | 2 |
| 1012 | LIM and SH3 domain protein 1 | Q14847 | 372 | 24.15 | 9 |
| 1013 | LIM domain and actin-binding protein 1 | Q9UHB6 | 377 | 16.21 | 12 |
| 1014 | LIM domain only protein 7 | Q8WWI1 | 126 | 5.01 | 4 |
| 1015 | Lin-7 homolog A | Q32LM6 | 91 | 10.73 | 2 |
| 1016 | Lipid phosphate phosphatase-related protein type 4 | Q7Z2D5 | 43 | 3.41 | 2 |
| 1017 | Lipopolysaccharide-responsive and beige-like anchor protein | P50851 | 80 | 1.54 | 2 |
| 1018 | Liprin-alpha-1 | Q13136 | 59 | 3.91 | 4 |
| 1019 | LisH domain and HEAT repeat-containing protein KIAA1468 | Q9P260 | 43 | 1.86 | 2 |
| 1020 | L-lactate dehydrogenase A chain | P00338 | 420 | 25.30 | 11 |
| 1021 | L-lactate dehydrogenase B chain | P07195 | 459 | 22.16 | 10 |
| 1022 | Lon protease homolog | P36776 | 132 | 4.83 | 4 |
| 1023 | Long-chain fatty acid transport protein 4 | Q6P1M0 | 62 | 2.50 | 2 |
| 1024 | Low-density lipoprotein receptor-related protein 11 | Q86VZ4 | 56 | 14.77 | 4 |
| 1025 | Lupus La protein | P05455 | 233 | 9.56 | 4 |
| 1026 | L-xylulose reductase | Q7Z4W1 | 131 | 16.46 | 4 |
| 1027 | Lymphocyte antigen 75 | O60449 | 41 | 0.94 | 2 |
| 1028 | Lymphoid-restricted membrane protein | Q12912 | 53 | 1.62 | 2 |
| 1029 | Lysine-specific demethylase 2A | Q9Y2K7 | 42 | 2.91 | 2 |
| 1030 | Lysine-specific demethylase 3A | Q9Y4C1 | 67 | 3.18 | 4 |
| 1031 | Lysine-specific demethylase 4A | O75164 | 46 | 1.79 | 2 |
| 1032 | Lysine-specific demethylase 5D | Q9BY66 | 70 | 3.05 | 4 |
| 1033 | Lysine-specific histone demethylase 1 A | O60341 | 64 | 2.93 | 2 |
| 1034 | Lysosomal acid phosphatase | P11117 | 58 | 5.20 | 3 |
| 1035 | Lysosomal alpha-mannosidase | O00754 | 68 | 2.78 | 2 |
| 1036 | Lysosomal-trafficking regulator | Q99698 | 74 | 0.76 | 3 |
| 1037 | Lysosome membrane protein 2 | Q14108 | 65 | 5.03 | 2 |
| 1038 | Lysozyme g-like protein 2 | Q86SG7 | 81 | 0.89 | 5 |
| 1039 | Lysyl-tRNA synthetase | Q15046 | 59 | 3.36 | 2 |
| 1040 | Macrophage-capping protein | P40121 | 47 | 6.61 | 2 |
| 1041 | MAGUK p55 subfamily member 2 | Q14168 | 44 | 3.16 | 2 |
| 1042 | Major centromere autoantigen B | P07199 | 45 | 5.34 | 2 |
| 1043 | Malate dehydrogenase, mitochondrial | P40926 | 241 | 18.05 | 5 |
| 1044 | Mannose-1-phosphate guanyltransferase alpha | Q96IJ6 | 72 | 8.10 | 5 |
| 1045 | Mannose-1-phosphate guanyltransferase beta | Q9Y5P6 | 51 | 5.43 | 2 |
| 1046 | Mannosyl-oligosaccharide glucosidase | Q13724 | 203 | 9.44 | 7 |
| 1047 | MAP kinase-activating death domain protein | Q8WXG6 | 42 | 1.28 | 3 |
| 1048 | MAP7 domain-containing protein 2 | Q96T17 | 50 | 3.14 | 2 |
| 1049 | Matrin-3 | P43243 | 783 | 22.90 | 14 |
| 1050 | Matrix-remodeling-associated protein 5 | Q9NR99 | 92 | 2.05 | 5 |
| 1051 | MAX gene-associated protein | Q8IWI9 | 50 | 3.26 | 2 |
| 1052 | Max-like protein X | Q9UH92 | 55 | 8.05 | 2 |
| 1053 | MBT domain-containing protein 1 | Q05BQ5 | 66 | 3.02 | 2 |
| 1054 | MDS1 and EVI1 complex locus protein EVI1 | Q03112 | 47 | 2.58 | 3 |
| 1055 | Medium-chain specific acyl-CoA dehydrogenase, mitochondrial | P11310 | 117 | 16.15 | 5 |
| 1056 | Melanoma-associated antigen D2 | Q9UNF1 | 44 | 3.65 | 2 |
| 1057 | Melanoma-associated antigen E1 | Q9HCI5 | 41 | 2.09 | 2 |
| 1058 | Membrane-associated guanylate kinase, WW and PDZ domain-containing protein 1 | Q96QZ7 | 45 | 1.64 | 2 |
| 1059 | Membrane-associated guanylate kinase, WW and PDZ domain-containing protein 2 | Q86UL8 | 95 | 2.82 | 6 |
| 1060 | Membrane-associated guanylate kinase, WW and PDZ domain-containing protein 3 | Q5TCQ9 | 65 | 2.30 | 4 |
| 1061 | Membrane-associated progesterone receptor component 1 | O00264 | 246 | 20.62 | 6 |
| 1062 | Mesothelin-like protein | Q96KJ4 | 42 | 1.99 | 3 |
| 1063 | Metal regulatory transcription factor 1 | Q14872 | 42 | 3.45 | 2 |
| 1064 | Metaxin-1 | Q13505 | 48 | 5.15 | 2 |
| 1065 | Methionine adenosyltransferase 2 subunit beta | Q9NZL9 | 140 | 14.29 | 3 |
| 1066 | Methionine aminopeptidase 1 | P53582 | 54 | 5.70 | 2 |
| 1067 | Methionine synthase | Q99707 | 48 | 2.37 | 2 |
| 1068 | Methionyl-tRNA synthetase | P56192 | 326 | 10.00 | 7 |
| 1069 | Methylmalonate-semialdehyde dehydrogenase [acylating], mitochondrial | Q02252 | 51 | 3.55 | 2 |
| 1070 | Methylosome subunit pICln | P54105 | 149 | 10.97 | 2 |
| 1071 | Methyltransferase-like protein 11B | Q5VVY1 | 51 | 6.55 | 2 |
| 1072 | MICAL-like protein 2 | Q8IY33 | 85 | 5.09 | 4 |
| 1073 | Microtubule-actin cross-linking factor 1, isoform 4 | Q96PK2 | 147 | 1.99 | 10 |
| 1074 | Microtubule-actin cross-linking factor 1, isoforms 1/2/3/5 | Q9UPN3 | 161 | 2.34 | 11 |
| 1075 | Microtubule-associated protein 1A | P78559 | 84 | 2.07 | 6 |
| 1076 | Microtubule-associated protein 4 | P27816 | 309 | 12.38 | 7 |
| 1077 | Microtubule-associated protein 9 | Q49MG5 | 44 | 4.02 | 2 |
| 1078 | Microtubule-associated protein RP/EB family member 1 | Q15691 | 335 | 23.60 | 4 |
| 1079 | Microtubule-associated serine/threonine-protein kinase 1 | Q9Y2H9 | 73 | 3.80 | 4 |
| 1080 | Microtubule-associated serine/threonine-protein kinase 4 | O15021 | 46 | 3.70 | 2 |
| 1081 | Microtubule-associated tumor suppressor 1 | Q9ULD2 | 86 | 3.29 | 4 |
| 1082 | Midasin | Q9NU22 | 76 | 0.80 | 5 |
| 1083 | Mitochondrial 28S ribosomal protein S34 | P82930 | 51 | 5.50 | 2 |
| 1084 | Mitochondrial import inner membrane translocase subunit TIM44 | O43615 | 116 | 10.36 | 4 |
| 1085 | Mitochondrial import inner membrane translocase subunit TIM50 | Q3ZCQ8 | 250 | 22.66 | 7 |
| 1086 | Mitochondrial import receptor subunit TOM34 | Q15785 | 145 | 10.68 | 3 |
| 1087 | Mitochondrial import receptor subunit TOM40 homolog | O96008 | 93 | 8.86 | 2 |
| 1088 | Mitochondrial inner membrane protein | Q16891 | 347 | 11.74 | 8 |
| 1089 | Mitochondrial Rho GTPase 1 | Q8IXI2 | 51 | 3.41 | 3 |
| 1090 | Mitochondrial-processing peptidase subunit alpha | Q10713 | 232 | 10.29 | 6 |
| 1091 | Mitochondrial-processing peptidase subunit beta, mitochondrial | O75439 | 94 | 9.20 | 3 |
| 1092 | Mitogen-activated protein kinase 1 | P28482 | 357 | 29.64 | 10 |
| 1093 | Mitogen-activated protein kinase 13 | O15264 | 102 | 10.70 | 5 |
| 1094 | Mitogen-activated protein kinase 15 | Q8TD08 | 67 | 6.43 | 3 |
| 1095 | Mitogen-activated protein kinase 4 | P31152 | 46 | 3.23 | 2 |
| 1096 | Mitogen-activated protein kinase 7 | Q13164 | 73 | 6.38 | 4 |
| 1097 | Mitotic checkpoint serine/threonine-protein kinase BUB1 beta | Q8WV50 | 42 | 2.67 | 2 |
| 1098 | MKL/myocardin-like protein 1 | Q969V6 | 45 | 3.26 | 3 |
| 1099 | MLL1/MLL complex subunit KIAA1267 | Q7Z3B3 | 50 | 3.93 | 2 |
| 1100 | MMS19 nucleotide excision repair protein homolog | Q96T76 | 104 | 3.10 | 3 |
| 1101 | Moesin | P26038 | 195 | 7.12 | 6 |
| 1102 | Monocarboxylate transporter 8 | P36021 | 52 | 3.06 | 2 |
| 1103 | MORC family CW-type zinc finger protein 4 | Q8TE76 | 43 | 1.92 | 2 |
| 1104 | Mortality factor 4-like protein 1 | Q9UBU8 | 43 | 11.52 | 4 |
| 1105 | MOSC domain-containing protein 1, mitochondrial | Q5VT66 | 45 | 7.54 | 2 |
| 1106 | M-phase inducer phosphatase 1 | P30304 | 74 | 3.93 | 3 |
| 1107 | mRNA export factor | P78406 | 48 | 6.79 | 3 |
| 1108 | mRNA-decapping enzyme 1B | Q8IZD4 | 45 | 6.15 | 2 |
| 1109 | Msx2-interacting protein | Q96T58 | 54 | 0.55 | 2 |
| 1110 | Mucin-16 | Q8WXI7 | 86 | 0.19 | 3 |
| 1111 | Multifunctional protein ADE2 | P22234 | 449 | 16.51 | 6 |
| 1112 | Multiple C2 and transmembrane domain-containing protein 1 | Q6DN14 | 51 | 2.19 | 2 |
| 1113 | Multiple epidermal growth factor-like domains 8 | Q7Z7M0 | 40 | 1.55 | 2 |
| 1114 | MutS protein homolog 4 | O15457 | 78 | 6.91 | 4 |
| 1115 | Myb-binding protein 1A | Q9BQG0 | 205 | 6.25 | 4 |
| 1116 | Myeloperoxidase | P05164 | 56 | 4.03 | 3 |
| 1117 | Myoferlin | Q9NZM1 | 116 | 2.43 | 2 |
| 1118 | Myomegalin | Q5VU43 | 45 | 1.68 | 2 |
| 1119 | Myomesin-2 | P54296 | 40 | 1.37 | 2 |
| 1120 | Myosin IIIA | Q8NEV4 | 45 | 1.61 | 2 |
| 1121 | Myosin light chain kinase 2, skeletal/cardiac muscle | Q9H1R3 | 42 | 9.67 | 2 |
| 1122 | Myosin regulatory light chain 2, nonsarcomeric | P19105 | 88 | 12.35 | 2 |
| 1123 | Myosin-10 | P35580 | 1273 | 15.36 | 27 |
| 1124 | Myosin-15 | Q9Y2K3 | 50 | 1.79 | 3 |
| 1125 | Myosin-7 | P12883 | 52 | 11.20 | 2 |
| 1126 | Myosin-9 | P35579 | 784 | 11.13 | 15 |
| 1127 | Myosin-binding protein C, cardiac-type | Q14896 | 53 | 1.49 | 2 |
| 1128 | Myosin-Ib | O43795 | 101 | 6.07 | 3 |
| 1129 | Myosin-Ic | O00159 | 478 | 13.76 | 16 |
| 1130 | Myosin-Id | O94832 | 200 | 6.11 | 6 |
| 1131 | Myosin-IXa | B2RTY4 | 52 | 0.98 | 3 |
| 1132 | Myosin-IXb | Q13459 | 55 | 1.53 | 3 |
| 1133 | Myosin-Va | Q9Y4I1 | 43 | 1.46 | 2 |
| 1134 | Myosin-VIIa | Q13402 | 48 | 1.05 | 2 |
| 1135 | Myosin-XVIIIa | Q92614 | 60 | 1.57 | 3 |
| 1136 | Myristoylated alanine-rich C-kinase substrate | P29966 | 243 | 14.80 | 4 |
| 1137 | N(G),N(G)-dimethylarginine dimethylaminohydrolase 1 | O94760 | 40 | 8.07 | 2 |
| 1138 | N(G),N(G)-dimethylarginine dimethylaminohydrolase 2 | O95865 | 136 | 22.15 | 5 |
| 1139 | Na(+)/H(+) exchange regulatory cofactor NHE-RF1 | O14745 | 305 | 24.09 | 6 |
| 1140 | Na(+)/H(+) exchange regulatory cofactor NHE-RF3 | Q5T2W1 | 69 | 5.39 | 3 |
| 1141 | N-acetylserotonin O-methyltransferase-like protein | O95671 | 63 | 3.14 | 2 |
| 1142 | NACHT, LRR and PYD domains-containing protein 5 | P59047 | 42 | 2.17 | 2 |
| 1143 | NAD(P) transhydrogenase, mitochondrial | Q13423 | 83 | 2.03 | 2 |
| 1144 | NAD-dependent deacetylase sirtuin-1 | Q96EB6 | 71 | 6.02 | 3 |
| 1145 | NADH dehydrogenase (Ubiquinone) flavoprotein 3 | Q8WU60 | 43 | 4.02 | 2 |
| 1146 | NADH dehydrogenase [ubiquinone] flavoprotein 1, mitochondrial | P49821 | 55 | 5.60 | 2 |
| 1147 | NADH dehydrogenase [ubiquinone] iron-sulfur protein 2, mitochondrial | O75306 | 44 | 1.89 | 2 |
| 1148 | NADH-ubiquinone oxidoreductase 75 kDa subunit, mitochondrial | P28331 | 49 | 3.30 | 2 |
| 1149 | NADP-dependent malic enzyme | P48163 | 99 | 4.37 | 2 |
| 1150 | N-alpha-acetyltransferase 15, NatA auxiliary subunit | Q9BXJ9 | 45 | 1.96 | 2 |
| 1151 | Natural cytotoxicity triggering receptor 3 | O14931 | 46 | 14.12 | 2 |
| 1152 | Nck-associated protein 1 | Q9Y2A7 | 101 | 3.37 | 4 |
| 1153 | Nebulette | O76041 | 55 | 4.44 | 2 |
| 1154 | Necdin | Q99608 | 41 | 6.54 | 2 |
| 1155 | NEDD8-conjugating enzyme Ubc12 | P61081 | 51 | 9.84 | 2 |
| 1156 | Negative elongation factor B | Q8WX92 | 79 | 7.22 | 3 |
| 1157 | Nesprin-1 | Q8NF91 | 111 | 0.70 | 6 |
| 1158 | Nesprin-2 | Q8WXH0 | 77 | 0.62 | 4 |
| 1159 | Neurabin-1 | Q9ULJ8 | 49 | 1.55 | 2 |
| 1160 | Neurexin-1-beta | P58400 | 44 | 9.27 | 2 |
| 1161 | Neuroblast differentiation-associated protein AHNAK | Q09666 | 65 | 1.63 | 3 |
| 1162 | Neurofilament heavy polypeptide | P12036 | 74 | 3.92 | 4 |
| 1163 | Neurogenin-2 | Q9H2A3 | 60 | 12.13 | 3 |
| 1164 | Neuron navigator 1 | Q8NEY1 | 54 | 1.07 | 2 |
| 1165 | Neuron navigator 2 | Q8IVL1 | 102 | 3.00 | 6 |
| 1166 | Neutral alpha-glucosidase AB | Q14697 | 554 | 21.53 | 18 |
| 1167 | NF-kappa-B inhibitor-like protein 2 | Q96HA7 | 40 | 1.56 | 2 |
| 1168 | Nibrin | O60934 | 42 | 2.79 | 2 |
| 1169 | Nicotinamide phosphoribosyltransferase | P43490 | 48 | 2.97 | 2 |
| 1170 | Nicotinate phosphoribosyltransferase | Q6XQN6 | 45 | 5.58 | 2 |
| 1171 | Nicotinate-nucleotide pyrophosphorylase [carboxylating] | Q15274 | 135 | 11.78 | 3 |
| 1172 | NIF3-like protein 1 | Q9GZT8 | 49 | 9.52 | 2 |
| 1173 | Ninein | Q8N4C6 | 73 | 3.05 | 3 |
| 1174 | Ninein-like protein | Q9Y2I6 | 44 | 3.62 | 3 |
| 1175 | Nitric oxide synthase, brain | P29475 | 46 | 1.61 | 2 |
| 1176 | Nodal modulator 1 | Q15155 | 199 | 4.71 | 4 |
| 1177 | Non-histone chromosomal protein HMG-14 | P05114 | 109 | 27.27 | 2 |
| 1178 | Non-receptor tyrosine-protein kinase TYK2 | P29597 | 47 | 2.02 | 3 |
| 1179 | Non-syndromic hearing impairment protein 5 | O60443 | 50 | 3.83 | 2 |
| 1180 | NSFL1 cofactor p47 | Q9UNZ2 | 403 | 31.72 | 10 |
| 1181 | N-terminal kinase-like protein | Q96KG9 | 51 | 2.67 | 2 |
| 1182 | Nuclear autoantigen Sp-100 | P23497 | 50 | 2.16 | 2 |
| 1183 | Nuclear autoantigenic sperm protein | P49321 | 149 | 6.22 | 3 |
| 1184 | Nuclear factor NF-kappa-B p100 subunit | Q00653 | 83 | 2.79 | 3 |
| 1185 | Nuclear migration protein nudC | Q9Y266 | 123 | 7.25 | 2 |
| 1186 | Nuclear mitotic apparatus protein 1 | Q14980 | 380 | 10.03 | 10 |
| 1187 | Nuclear pore complex protein Nup107 | P57740 | 50 | 2.70 | 2 |
| 1188 | Nuclear pore complex protein Nup153 | P49790 | 56 | 2.78 | 3 |
| 1189 | Nuclear pore complex protein Nup205 | Q92621 | 142 | 2.93 | 4 |
| 1190 | Nuclear pore complex protein Nup214 | P35658 | 123 | 2.88 | 2 |
| 1191 | Nuclear pore complex protein Nup88 | Q99567 | 68 | 3.64 | 2 |
| 1192 | Nuclear pore complex protein Nup93 | Q8N1F7 | 302 | 10.70 | 8 |
| 1193 | Nuclear pore membrane glycoprotein 210 | Q8TEM1 | 206 | 4.35 | 6 |
| 1194 | Nuclear protein Hcc-1 | P82979 | 123 | 13.97 | 2 |
| 1195 | Nuclear receptor coactivator 1 | Q15788 | 88 | 3.40 | 4 |
| 1196 | Nuclear receptor coactivator 5 | Q9HCD5 | 50 | 3.70 | 2 |
| 1197 | Nuclear receptor corepressor 1 | O75376 | 60 | 1.31 | 3 |
| 1198 | Nuclear receptor ROR-alpha | P35398 | 54 | 7.05 | 3 |
| 1199 | Nuclease-sensitive element-binding protein 1 | P67809 | 122 | 11.46 | 4 |
| 1200 | Nucleobindin-1 | Q02818 | 46 | 3.69 | 2 |
| 1201 | Nucleolar and coiled-body phosphoprotein 1 | Q14978 | 229 | 11.59 | 4 |
| 1202 | Nucleolar GTP-binding protein 2 | Q13823 | 77 | 5.75 | 4 |
| 1203 | Nucleolar MIF4G domain-containing protein 1 | Q5C9Z4 | 61 | 1.86 | 2 |
| 1204 | Nucleolar protein 56 | O00567 | 325 | 13.80 | 9 |
| 1205 | Nucleolar protein 58 | Q9Y2X3 | 75 | 10.09 | 4 |
| 1206 | Nucleolar protein 7 | Q9UMY1 | 77 | 10.36 | 4 |
| 1207 | Nucleolar RNA helicase 2 | Q9NR30 | 402 | 12.23 | 8 |
| 1208 | Nucleolar transcription factor 1 | P17480 | 49 | 2.75 | 2 |
| 1209 | Nucleolin | P19338 | 991 | 25.18 | 16 |
| 1210 | Nucleophosmin | P06748 | 977 | 57.50 | 15 |
| 1211 | Nucleoporin GLE1 | Q53GS7 | 44 | 3.73 | 2 |
| 1212 | Nucleoporin NUP188 homolog | Q5SRE5 | 94 | 5.24 | 4 |
| 1213 | Nucleoside diphosphate kinase A | P15531 | 456 | 55.63 | 10 |
| 1214 | Nucleosome assembly protein 1-like 1 | P55209 | 68 | 5.63 | 2 |
| 1215 | Nucleosome assembly protein 1-like 4 | Q99733 | 59 | 4.80 | 2 |
| 1216 | Nucleotide-binding protein-like | Q8TB37 | 45 | 7.21 | 2 |
| 1217 | NudC domain-containing protein 2 | Q8WVJ2 | 89 | 12.74 | 2 |
| 1218 | Obg-like ATPase 1 | Q9NTK5 | 344 | 25.25 | 9 |
| 1219 | Obscurin | Q5VST9 | 129 | 1.46 | 10 |
| 1220 | Oligodendrocyte transcription factor 2 | Q13516 | 48 | 26.32 | 2 |
| 1221 | Omega-amidase NIT2 | Q9NQR4 | 150 | 11.96 | 3 |
| 1222 | Opioid growth factor receptor | Q9NZT2 | 79 | 3.78 | 4 |
| 1223 | Optineurin | Q96CV9 | 41 | 3.64 | 2 |
| 1224 | Orexin receptor type 2 | O43614 | 62 | 4.28 | 3 |
| 1225 | Ornithine aminotransferase, mitochondrial | P04181 | 359 | 19.36 | 6 |
| 1226 | Ornithine carbamoyltransferase, mitochondrial | P00480 | 50 | 2.43 | 3 |
| 1227 | Paired amphipathic helix protein Sin3a | Q96ST3 | 84 | 3.46 | 2 |
| 1228 | Paired amphipathic helix protein Sin3b | O75182 | 45 | 2.93 | 2 |
| 1229 | Paired box protein Pax-4 | O43316 | 43 | 7.43 | 2 |
| 1230 | Papilin | O95428 | 45 | 1.38 | 2 |
| 1231 | Pappalysin-2 | Q9BXP8 | 64 | 1.23 | 3 |
| 1232 | Paralemmin | O75781 | 53 | 7.49 | 3 |
| 1233 | Paraneoplastic antigen-like protein 5 | Q96PV4 | 47 | 3.13 | 2 |
| 1234 | Paraplegin | Q9UQ90 | 51 | 3.02 | 2 |
| 1235 | Paraspeckle component 1 | Q8WXF1 | 327 | 19.96 | 9 |
| 1236 | Partitioning defective 6 homolog beta | Q9BYG5 | 118 | 11.02 | 4 |
| 1237 | Partitioning-defective 3 homolog B | Q8TEW8 | 56 | 1.58 | 3 |
| 1238 | PCI domain-containing protein 2 | Q5JVF3 | 92 | 9.76 | 3 |
| 1239 | PCTP-like protein | Q9Y365 | 121 | 15.12 | 4 |
| 1240 | PDZ and LIM domain protein 1 | O00151 | 303 | 21.95 | 8 |
| 1241 | PDZ domain-containing protein 7 | Q9H5P4 | 42 | 4.26 | 2 |
| 1242 | PDZ domain-containing protein 8 | Q8NEN9 | 47 | 2.34 | 2 |
| 1243 | Pecanex-like protein 2 | A6NKB5 | 51 | 3.22 | 2 |
| 1244 | Peptidylprolyl cis-trans isomerase A-like 4B | Q9Y536 | 601 | 61.21 | 15 |
| 1245 | Peptidyl-prolyl cis-trans isomerase B | P23284 | 70 | 8.17 | 2 |
| 1246 | Peptidyl-prolyl cis-trans isomerase FKBP3 | Q00688 | 84 | 8.48 | 2 |
| 1247 | Peptidyl-prolyl cis-trans isomerase FKBP4 | Q02790 | 156 | 10.92 | 5 |
| 1248 | Pericentriolar material 1 protein | Q15154 | 51 | 1.53 | 2 |
| 1249 | Perilipin-2 | Q99541 | 60 | 5.03 | 2 |
| 1250 | Perilipin-3 | O60664 | 259 | 14.29 | 5 |
| 1251 | Period circadian protein homolog 2 | O15055 | 47 | 1.91 | 2 |
| 1252 | Periodic tryptophan protein 2 homolog | Q15269 | 49 | 62.07 | 2 |
| 1253 | Peripheral plasma membrane protein CASK | O14936 | 74 | 2.70 | 2 |
| 1254 | Peripherin | P41219 | 140 | 3.62 | 3 |
| 1255 | Peroxiredoxin-1 | Q06830 | 518 | 42.71 | 16 |
| 1256 | Peroxiredoxin-2 | P32119 | 513 | 41.12 | 10 |
| 1257 | Peroxisomal multifunctional enzyme type 2 | P51659 | 44 | 2.99 | 2 |
| 1258 | Peroxisomal NADH pyrophosphatase NUDT12 | Q9BQG2 | 48 | 2.38 | 2 |
| 1259 | Pescadillo homolog 1 | O00541 | 45 | 3.26 | 2 |
| 1260 | PH domain leucine-rich repeat-containing protein phosphatase | O60346 | 88 | 3.67 | 5 |
| 1261 | PHD finger protein 2 | O75151 | 46 | 2.91 | 2 |
| 1262 | Phenylalanyl-tRNA synthetase alpha chain | Q9Y285 | 53 | 4.33 | 2 |
| 1263 | Phenylalanyl-tRNA synthetase beta chain | Q9NSD9 | 231 | 11.11 | 8 |
| 1264 | Phosphatase and actin regulator 3 | Q96KR7 | 51 | 3.24 | 2 |
| 1265 | Phosphate carrier protein, mitochondrial | Q00325 | 231 | 10.11 | 3 |
| 1266 | Phosphatidylethanolamine-binding protein 1 | P30086 | 67 | 8.60 | 2 |
| 1267 | Phosphatidylinositol-4-phosphate 3-kinase C2 domain-containing subunit alpha | O00443 | 48 | 0.89 | 2 |
| 1268 | Phosphoacetylglucosamine mutase | O95394 | 72 | 14.57 | 2 |
| 1269 | Phosphoglucomutase-1 | P36871 | 46 | 3.57 | 2 |
| 1270 | Phosphoglucomutase-2 | Q96G03 | 46 | 3.44 | 2 |
| 1271 | Phosphoglycerate kinase 1 | P00558 | 1209 | 56.12 | 29 |
| 1272 | Phosphoglycerate mutase 1 | P18669 | 542 | 24.80 | 7 |
| 1273 | Phosphopantothenate--cysteine ligase | Q9HAB8 | 67 | 11.58 | 3 |
| 1274 | Phosphoribosyl pyrophosphate synthetase-associated protein 2 | O60256 | 104 | 9.21 | 3 |
| 1275 | Phosphoribosylformylglycinamidine synthase | O15067 | 309 | 10.50 | 7 |
| 1276 | Phosphoribosyltransferase domain-containing protein 1 | Q9NRG1 | 76 | 12.44 | 3 |
| 1277 | Phosphorylase b kinase regulatory subunit beta | Q93100 | 43 | 1.83 | 2 |
| 1278 | Phosphoserine aminotransferase 1 | Q9Y617 | 41 | 6.48 | 3 |
| 1279 | Phosphoserine phosphatase | P78330 | 92 | 12.00 | 4 |
| 1280 | Phosphotriesterase-related protein | Q96BW5 | 59 | 9.17 | 2 |
| 1281 | Piwi-like protein 3 | Q7Z3Z3 | 40 | 1.93 | 2 |
| 1282 | PKHD domain-containing transmembrane protein C17orf101 | Q6PK18 | 50 | 6.04 | 2 |
| 1283 | Plakophilin-2 | Q99959 | 492 | 16.97 | 14 |
| 1284 | Plakophilin-3 | Q9Y446 | 72 | 4.14 | 3 |
| 1285 | Plasma membrane calcium-transporting ATPase 4 | P23634 | 67 | 2.26 | 3 |
| 1286 | Plasminogen activator inhibitor 1 RNA-binding protein | Q8NC51 | 262 | 13.48 | 6 |
| 1287 | Plastin-1 | Q14651 | 119 | 5.56 | 3 |
| 1288 | Plastin-2 | P13796 | 679 | 20.77 | 14 |
| 1289 | Plastin-3 | P13797 | 361 | 12.92 | 10 |
| 1290 | Platelet-activating factor acetylhydrolase IB subunit alpha | P43034 | 83 | 7.09 | 2 |
| 1291 | Platelet-activating factor acetylhydrolase IB subunit beta | P68402 | 267 | 20.96 | 4 |
| 1292 | Pleckstrin homology domain-containing family O member 1 | Q53GL0 | 67 | 2.91 | 3 |
| 1293 | Plectin 1 | Q15149 | 227 | 4.07 | 5 |
| 1294 | Plexin-B1 [Precursor] | O43157 | 46 | 1.73 | 4 |
| 1295 | Plexin-C1 | O60486 | 47 | 1.59 | 2 |
| 1296 | PNMA-like protein 2 | Q9ULN7 | 72 | 5.51 | 2 |
| 1297 | Poly [ADP-ribose] polymerase 1 | P09874 | 444 | 12.93 | 10 |
| 1298 | Poly [ADP-ribose] polymerase 14 | Q460N5 | 59 | 2.56 | 4 |
| 1299 | Poly(RC) binding protein 2 | Q15366 | 344 | 32.95 | 12 |
| 1300 | Poly(rC)-binding protein 1 | Q15365 | 420 | 28.09 | 11 |
| 1301 | Poly(rC)-binding protein 2 | Q15366 | 90 | 13.40 | 4 |
| 1302 | Poly(rC)-binding protein 3 | P57721 | 186 | 17.45 | 8 |
| 1303 | Polyadenylate-binding protein 1 | P11940 | 89 | 3.63 | 2 |
| 1304 | Polycystic kidney disease protein 1-like 1 | Q8TDX9 | 53 | 1.05 | 3 |
| 1305 | Polymerase delta-interacting protein 2 (p38) | Q9Y2S7 | 118 | 14.13 | 4 |
| 1306 | Polymerase delta-interacting protein 3 | Q9BY77 | 117 | 10.31 | 4 |
| 1307 | Polypyrimidine tract-binding protein 1 | P26599 | 235 | 13.18 | 5 |
| 1308 | Polyribonucleotide nucleotidyltransferase 1, mitochondrial | Q8TCS8 | 148 | 7.66 | 7 |
| 1309 | Potassium channel subfamily K member 12 | Q9HB15 | 52 | 4.88 | 3 |
| 1310 | Potassium voltage-gated channel subfamily B member 2 | Q92953 | 56 | 2.20 | 3 |
| 1311 | Potassium voltage-gated channel subfamily H member 6 | Q9H252 | 51 | 2.32 | 2 |
| 1312 | Potassium voltage-gated channel subfamily KQT member 2 | O43526 | 58 | 3.33 | 3 |
| 1313 | Potassium/sodium hyperpolarization-activated cyclic nucleotide-gated channel 3 | Q9P1Z3 | 55 | 4.01 | 3 |
| 1314 | Potassium-transporting ATPase alpha chain 1 | P20648 | 142 | 5.67 | 3 |
| 1315 | POTE ankyrin domain family member F | A5A3E0 | 82 | 24.18 | 2 |
| 1316 | PR domain zinc finger protein 15 | P57071 | 65 | 3.32 | 5 |
| 1317 | PR domain zinc finger protein 16 | Q9HAZ2 | 41 | 0.88 | 2 |
| 1318 | Prefoldin subunit 6 | O15212 | 49 | 14.73 | 2 |
| 1319 | Pre-mRNA-processing factor 19 | Q9UMS4 | 63 | 2.38 | 2 |
| 1320 | Pre-mRNA-processing factor 6 | O94906 | 163 | 5.63 | 7 |
| 1321 | Pre-mRNA-processing-splicing factor 8 | Q6P2Q9 | 614 | 8.74 | 18 |
| 1322 | Pre-mRNA-splicing factor ISY1 homolog | Q9ULR0 | 52 | 8.46 | 2 |
| 1323 | Pre-mRNA-splicing factor SYF1 | Q9HCS7 | 50 | 9.17 | 2 |
| 1324 | Prenylcysteine oxidase 1 | Q9UHG3 | 63 | 3.76 | 2 |
| 1325 | Pre-rRNA-processing protein TSR1 homolog | Q2NL82 | 202 | 9.08 | 8 |
| 1326 | Prickle-like protein 1 | Q96MT3 | 49 | 2.77 | 2 |
| 1327 | Probable 2-oxoglutarate dehydrogenase E1 component DHKTD1, mitochondrial | Q96HY7 | 53 | 2.94 | 2 |
| 1328 | Probable alanyl-tRNA synthetase, mitochondrial | Q5JTZ9 | 67 | 2.34 | 2 |
| 1329 | Probable aminopeptidase NPEPL1 | Q8NDH3 | 41 | 3.82 | 2 |
| 1330 | Probable ATP-dependent RNA helicase DDX10 | Q13206 | 103 | 6.04 | 5 |
| 1331 | Probable ATP-dependent RNA helicase DDX17 | Q92841 | 339 | 11.89 | 10 |
| 1332 | Probable ATP-dependent RNA helicase DDX23 | Q9BUQ8 | 112 | 4.27 | 4 |
| 1333 | Probable ATP-dependent RNA helicase DDX27 | Q96GQ7 | 73 | 3.90 | 3 |
| 1334 | Probable ATP-dependent RNA helicase DDX31 | Q9H8H2 | 56 | 4.70 | 3 |
| 1335 | Probable ATP-dependent RNA helicase DDX46 | Q7L014 | 240 | 10.27 | 5 |
| 1336 | Probable ATP-dependent RNA helicase DDX47 | Q9H0S4 | 219 | 9.23 | 4 |
| 1337 | Probable ATP-dependent RNA helicase DDX5 | P17844 | 136 | 7.49 | 5 |
| 1338 | Probable ATP-dependent RNA helicase DDX52 | Q9Y2R4 | 57 | 5.00 | 2 |
| 1339 | Probable ATP-dependent RNA helicase DDX6 | P26196 | 119 | 8.49 | 4 |
| 1340 | Probable carboxypeptidase PM20D1 | Q6GTS8 | 47 | 3.98 | 2 |
| 1341 | Probable cation-transporting ATPase 13A1 | Q9HD20 | 64 | 4.82 | 4 |
| 1342 | Probable E3 ubiquitin-protein ligase HERC2 | O95714 | 76 | 0.62 | 3 |
| 1343 | Probable global transcription activator SNF2L1 | P28370 | 203 | 7.51 | 4 |
| 1344 | Probable global transcription activator SNF2L2 | P51531 | 54 | 1.02 | 3 |
| 1345 | Probable G-protein coupled receptor 144 | Q7Z7M1 | 47 | 2.60 | 2 |
| 1346 | Probable G-protein coupled receptor 158 | Q5T848 | 56 | 1.48 | 2 |
| 1347 | Probable helicase senataxin | Q7Z333 | 66 | 0.90 | 3 |
| 1348 | Probable histidyl-tRNA synthetase, mitochondrial | P49590 | 44 | 3.36 | 2 |
| 1349 | Probable histone-lysine N-methyltransferase ASH1L | Q9NR48 | 74 | 3.34 | 4 |
| 1350 | Probable hydrolase PNKD | Q8N490 | 50 | 5.54 | 2 |
| 1351 | Probable methylcytosine dioxygenase TET2 | Q6N021 | 53 | 8.26 | 2 |
| 1352 | Probable rRNA-processing protein EBP2 | Q99848 | 126 | 13.40 | 3 |
| 1353 | Probable serine protease HTRA3 | P83110 | 61 | 11.76 | 3 |
| 1354 | Probable ubiquitin carboxyl-terminal hydrolase FAF-X | Q93008 | 511 | 7.54 | 13 |
| 1355 | Procollagen-lysine,2-oxoglutarate 5-dioxygenase 2 | O00469 | 229 | 8.96 | 6 |
| 1356 | Procollagen-lysine,2-oxoglutarate 5-dioxygenase 3 | O60568 | 86 | 10.49 | 5 |
| 1357 | Profilin-1 | P07737 | 396 | 50.72 | 5 |
| 1358 | Programmed cell death 6-interacting protein | Q8WUM4 | 158 | 5.99 | 5 |
| 1359 | Programmed cell death protein 10 | Q9BUL8 | 88 | 9.43 | 2 |
| 1360 | Programmed cell death protein 5 | O14737 | 114 | 17.74 | 2 |
| 1361 | Prohibitin | P35232 | 937 | 46.32 | 13 |
| 1362 | Prohibitin-2 | Q99623 | 855 | 55.18 | 15 |
| 1363 | Proliferating cell nuclear antigen | P12004 | 77 | 11.11 | 3 |
| 1364 | Proliferation-associated protein 2G4 | Q9UQ80 | 128 | 11.18 | 6 |
| 1365 | Proline-, glutamic acid-, leucine-rich protein 1 | Q8IZL8 | 135 | 4.60 | 4 |
| 1366 | Proline-rich protein 25 | Q96S07 | 74 | 9.20 | 3 |
| 1367 | Proline-rich transmembrane protein 3 | Q5FWE3 | 66 | 2.14 | 2 |
| 1368 | Proline-serine-threonine phosphatase-interacting protein 1 | O43586 | 44 | 5.05 | 2 |
| 1369 | Properdin | P27918 | 61 | 2.13 | 3 |
| 1370 | Propionyl-CoA carboxylase alpha chain, mitochondrial | P05165 | 45 | 3.84 | 2 |
| 1371 | ProSAP-interacting protein 1 | O60299 | 49 | 2.87 | 2 |
| 1372 | Prostaglandin F2 receptor negative regulator | Q9P2B2 | 50 | 2.38 | 2 |
| 1373 | Prostaglandin G/H synthase 2 | P35354 | 51 | 3.64 | 2 |
| 1374 | Proteasome activator complex subunit 2 | Q9UL46 | 83 | 12.61 | 3 |
| 1375 | Proteasome subunit alpha type-1 | P25786 | 277 | 24.16 | 7 |
| 1376 | Proteasome subunit alpha type-2 | P25787 | 225 | 18.45 | 5 |
| 1377 | Proteasome subunit alpha type-3 | P25788 | 348 | 26.38 | 7 |
| 1378 | Proteasome subunit alpha type-4 | P25789 | 134 | 8.43 | 3 |
| 1379 | Proteasome subunit alpha type-5 | P28066 | 225 | 14.11 | 3 |
| 1380 | Proteasome subunit alpha type-6 | P60900 | 147 | 13.01 | 4 |
| 1381 | Proteasome subunit alpha type-7 | O14818 | 261 | 21.37 | 5 |
| 1382 | Proteasome subunit alpha type-7-like | Q8TAA3 | 161 | 13.28 | 3 |
| 1383 | Proteasome subunit beta type-2 | P49721 | 58 | 22.39 | 3 |
| 1384 | Proteasome subunit beta type-5 | P28074 | 184 | 17.87 | 5 |
| 1385 | Proteasome subunit beta type-6 | P28072 | 226 | 17.57 | 5 |
| 1386 | Protein ajuba | Q96IF1 | 50 | 2.04 | 2 |
| 1387 | Protein arginine N-methyltransferase 1 | Q99873 | 107 | 12.36 | 3 |
| 1388 | Protein argonaute-2 | Q9UKV8 | 48 | 6.90 | 2 |
| 1389 | Protein BAT4 | O95872 | 57 | 9.55 | 3 |
| 1390 | Protein canopy homolog 3 | Q9BT09 | 47 | 17.48 | 2 |
| 1391 | Protein capicua homolog | Q96RK0 | 44 | 3.17 | 3 |
| 1392 | Protein CBFA2T1 | Q06455 | 59 | 4.97 | 3 |
| 1393 | Protein cramped-like | Q96RY5 | 67 | 1.76 | 2 |
| 1394 | Protein DEK | P35659 | 115 | 8.60 | 3 |
| 1395 | Protein diaphanous homolog 1 | O60610 | 544 | 13.44 | 8 |
| 1396 | Protein disulfide-isomerase | P07237 | 196 | 12.01 | 7 |
| 1397 | Protein disulfide-isomerase A3 | P30101 | 795 | 31.68 | 19 |
| 1398 | Protein disulfide-isomerase A4 | P13667 | 200 | 7.75 | 5 |
| 1399 | Protein disulfide-isomerase A6 | Q15084 | 1208 | 40.62 | 15 |
| 1400 | Protein DJ-1 | Q99497 | 167 | 17.46 | 4 |
| 1401 | Protein dpy-19 homolog 2 | Q6NUT2 | 54 | 2.37 | 2 |
| 1402 | Protein FAM105B | Q96BN8 | 44 | 5.70 | 2 |
| 1403 | Protein FAM115A | Q9Y4C2 | 43 | 2.07 | 2 |
| 1404 | Protein FAM116B | Q8NEG7 | 42 | 1.88 | 2 |
| 1405 | Protein FAM151A | Q8WW52 | 157 | 8.89 | 4 |
| 1406 | Protein FAM164A | Q96GY0 | 47 | 7.08 | 2 |
| 1407 | Protein FAM178A | Q8IX21 | 63 | 2.36 | 3 |
| 1408 | Protein FAM179A | Q6ZUX3 | 67 | 7.33 | 3 |
| 1409 | Protein FAM190A | Q9C0I3 | 51 | 2.11 | 2 |
| 1410 | Protein FAM38A | Q92508 | 79 | 2.26 | 4 |
| 1411 | Protein FAM50A | Q14320 | 186 | 20.94 | 6 |
| 1412 | Protein FAM65A | Q6ZS17 | 48 | 1.88 | 2 |
| 1413 | Protein FAM81A | Q8TBF8 | 41 | 3.01 | 2 |
| 1414 | Protein FAM83H | Q6ZRV2 | 45 | 3.16 | 2 |
| 1415 | Protein furry homolog-like | O94915 | 46 | 3.01 | 2 |
| 1416 | Protein GPR107 | Q5VW38 | 40 | 4.33 | 2 |
| 1417 | Protein Hook homolog 2 | Q96ED9 | 50 | 3.34 | 2 |
| 1418 | Protein Jumonji | Q92833 | 71 | 2.25 | 3 |
| 1419 | Protein KIAA0664 | O75153 | 727 | 16.88 | 19 |
| 1420 | Protein KIAA1967 | Q8N163 | 194 | 9.86 | 6 |
| 1421 | Protein kinase C and casein kinase substrate in neurons protein 2 | Q9UNF0 | 50 | 23.81 | 2 |
| 1422 | Protein kinase C delta type | Q05655 | 40 | 5.75 | 2 |
| 1423 | Protein kinase C epsilon type | Q02156 | 41 | 4.48 | 2 |
| 1424 | Protein kinase C eta type | P24723 | 46 | 2.64 | 2 |
| 1425 | Protein lin-7 homolog C | Q9NUP9 | 93 | 12.69 | 2 |
| 1426 | Protein LSM14 homolog A | Q8ND56 | 55 | 4.54 | 2 |
| 1427 | Protein monoglycylase TTLL8 | A6PVC2 | 47 | 4.15 | 2 |
| 1428 | Protein MTO1 homolog, mitochondrial | Q9Y2Z2 | 45 | 3.06 | 2 |
| 1429 | Protein NDRG1 | Q92597 | 84 | 7.61 | 2 |
| 1430 | Protein NLRC3 | Q7RTR2 | 50 | 3.86 | 3 |
| 1431 | Protein NLRC5 | Q86WI3 | 70 | 2.04 | 4 |
| 1432 | Protein phosphatase 1 regulatory subunit 12B | O60237 | 94 | 3.97 | 4 |
| 1433 | Protein phosphatase 1 regulatory subunit 3E | Q9H7J1 | 41 | 6.88 | 2 |
| 1434 | Protein phosphatase 1G | O15355 | 133 | 9.89 | 5 |
| 1435 | Protein phosphatase Slingshot homolog 1 | Q8WYL5 | 45 | 1.43 | 2 |
| 1436 | Protein RMD5 homolog A | Q9H871 | 40 | 21.43 | 3 |
| 1437 | Protein SCO1 homolog, mitochondrial | O75880 | 90 | 11.96 | 2 |
| 1438 | Protein SERAC1 | Q96JX3 | 58 | 5.65 | 3 |
| 1439 | Protein SET | Q01105 | 335 | 19.49 | 8 |
| 1440 | Protein shisa-2 homolog | Q6UWI4 | 41 | 12.88 | 2 |
| 1441 | Protein Shroom2 | Q13796 | 44 | 1.28 | 2 |
| 1442 | Protein Shroom3 | Q8TF72 | 54 | 2.68 | 3 |
| 1443 | Protein SOLO | Q8TER5 | 46 | 1.88 | 2 |
| 1444 | Protein transport protein Sec23A | Q15436 | 134 | 5.23 | 4 |
| 1445 | Protein transport protein Sec24C | P53992 | 52 | 1.94 | 2 |
| 1446 | Protein transport protein Sec31A | O94979 | 182 | 6.23 | 3 |
| 1447 | Protein transport protein Sec61 subunit beta | P60468 | 68 | 24.21 | 2 |
| 1448 | Protein unc-45 homolog B | Q8IWX7 | 41 | 2.26 | 2 |
| 1449 | Protein very KIND | Q76NI1 | 53 | 2.57 | 3 |
| 1450 | Protein Wiz | O95785 | 52 | 8.69 | 3 |
| 1451 | Protein Wnt-10a | Q9GZT5 | 51 | 8.87 | 2 |
| 1452 | Protein zyg-11 homolog B | Q9C0D3 | 47 | 2.92 | 2 |
| 1453 | Protein-glutamine gamma-glutamyltransferase 4 | P49221 | 44 | 2.19 | 2 |
| 1454 | Protein-L-isoaspartate(D-aspartate) O-methyltransferase | P22061 | 145 | 15.42 | 4 |
| 1455 | Protein-tyrosine kinase 2-beta | Q14289 | 45 | 1.88 | 2 |
| 1456 | Protocadherin alpha-1 | Q9Y5I3 | 68 | 3.05 | 2 |
| 1457 | Protocadherin alpha-5 | Q9Y5H7 | 48 | 2.36 | 2 |
| 1458 | Protocadherin Fat 4 | Q6V0I7 | 49 | 0.84 | 3 |
| 1459 | Protocadherin gamma-B4 | Q9UN71 | 43 | 2.24 | 2 |
| 1460 | Protocadherin-17 | O14917 | 56 | 2.14 | 2 |
| 1461 | Puromycin-sensitive aminopeptidase | P55786 | 204 | 6.09 | 6 |
| 1462 | Putative adenosylhomocysteinase 3 | Q96HN2 | 58 | 5.85 | 2 |
| 1463 | Putative ATP-dependent RNA helicase DHX30 | Q7L2E3 | 87 | 2.09 | 3 |
| 1464 | Putative deoxyribose-phosphate aldolase | Q9Y315 | 50 | 5.03 | 2 |
| 1465 | Putative heat shock protein HSP 90-beta 2 | Q58FF8 | 580 | 17.06 | 10 |
| 1466 | Putative helicase MOV-10 | Q9HCE1 | 79 | 5.68 | 5 |
| 1467 | Putative heterogeneous nuclear ribonucleoprotein A1-like protein 3 | P0C7M2 | 307 | 34.69 | 9 |
| 1468 | Putative hexokinase HKDC1 | Q2TB90 | 184 | 7.96 | 8 |
| 1469 | Putative high mobility group protein 1-like 10 | Q9UGV6 | 384 | 26.17 | 7 |
| 1470 | Putative methyltransferase NSUN5C | Q63ZY6 | 41 | 2.04 | 2 |
| 1471 | Putative Polycomb group protein ASXL2 | Q76L83 | 64 | 4.68 | 5 |
| 1472 | Putative pre-mRNA-splicing factor ATP-dependent RNA helicase DHX15 | O43143 | 281 | 9.94 | 8 |
| 1473 | Putative protein arginine N-methyltransferase 10 | Q6P2P2 | 47 | 4.38 | 3 |
| 1474 | Putative ribosomal RNA methyltransferase NOP2 | P46087 | 606 | 20.00 | 17 |
| 1475 | Putative RNA-binding protein Luc7-like 2 | Q9Y383 | 56 | 4.18 | 2 |
| 1476 | Putative splicing factor, arginine/serine-rich 14 | Q8IX01 | 90 | 4.91 | 4 |
| 1477 | Putative ubiquitin carboxyl-terminal hydrolase 17-like protein 1 | Q7RTZ2 | 81 | 3.58 | 3 |
| 1478 | Putative ubiquitin-conjugating enzyme E2 N-like | Q5JXB2 | 188 | 19.61 | 2 |
| 1479 | Putative uncharacterized protein C12orf47 | Q8N8E1 | 48 | 18.71 | 2 |
| 1480 | Putative uncharacterized protein C12orf63 | Q6ZTY8 | 52 | 1.67 | 2 |
| 1481 | Putative uncharacterized protein FLJ45355 | Q6ZSN1 | 42 | 11.66 | 2 |
| 1482 | Putative uncharacterized protein FLJ45840 | Q6ZS46 | 61 | 12.11 | 3 |
| 1483 | Putative uncharacterized protein PET112L (PET112-like) (Yeast) (PET112-like (Yeast), isoform CRA_a) | O75879 | 47 | 3.77 | 2 |
| 1484 | Putative uncharacterized protein UNQ6490/PRO21339 | Q6UXU1 | 63 | 10.71 | 2 |
| 1485 | Pyridoxal kinase | O00764 | 81 | 7.39 | 3 |
| 1486 | Pyridoxal-dependent decarboxylase domain-containing protein 1 | Q6P996 | 78 | 4.19 | 4 |
| 1487 | Pyridoxine-5'-phosphate oxidase | Q9NVS9 | 183 | 14.94 | 4 |
| 1488 | Pyrin and HIN domain-containing protein 1 | Q6K0P9 | 54 | 20.59 | 4 |
| 1489 | Pyrroline-5-carboxylate reductase 1, mitochondrial | P32322 | 118 | 10.34 | 4 |
| 1490 | Pyruvate dehydrogenase E1 component subunit alpha, somatic form, mitochondrial | P08559 | 157 | 12.82 | 6 |
| 1491 | Pyruvate dehydrogenase protein X component | O00330 | 144 | 7.58 | 4 |
| 1492 | Pyruvate kinase isozymes M1/M2 | P14618 | 409 | 13.40 | 8 |
| 1493 | Rab effector Noc2 | Q9UNE2 | 56 | 10.79 | 3 |
| 1494 | Rab GDP dissociation inhibitor beta | P50395 | 808 | 39.55 | 19 |
| 1495 | Rab GTPase-binding effector protein 2 | Q9H5N1 | 45 | 3.75 | 2 |
| 1496 | Rabenosyn-5 | Q9H1K0 | 51 | 2.48 | 2 |
| 1497 | Rabphilin-3A | Q9Y2J0 | 45 | 2.88 | 2 |
| 1498 | Ral guanine nucleotide dissociation stimulator-like 1 | Q9NZL6 | 40 | 2.21 | 2 |
| 1499 | RalA-binding protein 1 | Q15311 | 48 | 2.29 | 2 |
| 1500 | Ran GTPase-activating protein 1 | P46060 | 81 | 6.42 | 3 |
| 1501 | RANBP2-like and GRIP domain-containing protein 1/2 | Q68DN6 | 60 | 1.20 | 2 |
| 1502 | RanBP-type and C3HC4-type zinc finger-containing protein 1 | Q9BYM8 | 40 | 5.98 | 2 |
| 1503 | Rap guanine nucleotide exchange factor 6 | Q8TEU7 | 48 | 2.44 | 2 |
| 1504 | Rap guanine nucleotide exchange factor-like protein 1 | Q9UHV5 | 44 | 4.61 | 2 |
| 1505 | Rapamycin-insensitive companion of mTOR | Q6R327 | 59 | 1.00 | 2 |
| 1506 | Ras GTPase-activating protein SynGAP | Q96PV0 | 49 | 10.73 | 2 |
| 1507 | Ras GTPase-activating protein-binding protein 1 | Q13283 | 168 | 10.09 | 5 |
| 1508 | Ras GTPase-activating-like protein IQGAP1 | P46940 | 509 | 9.59 | 9 |
| 1509 | Ras GTPase-activating-like protein IQGAP2 | Q13576 | 56 | 2.34 | 3 |
| 1510 | RAS guanyl-releasing protein 4 | Q8TDF6 | 53 | 3.71 | 3 |
| 1511 | RAS protein activator like-3 | Q86YV0 | 44 | 2.67 | 2 |
| 1512 | RasGAP-activating-like protein 1 | O95294 | 44 | 3.06 | 2 |
| 1513 | Ras-GEF domain-containing family member 1B | Q0VAM2 | 50 | 6.57 | 3 |
| 1514 | Ras-related C3 botulinum toxin substrate 1 | P63000 | 58 | 5.76 | 2 |
| 1515 | Ras-related protein Rab-10 | P61026 | 199 | 22.50 | 4 |
| 1516 | Ras-related protein Rab-11B | Q15907 | 115 | 17.43 | 4 |
| 1517 | Ras-related protein Rab-13 | P51153 | 81 | 12.70 | 3 |
| 1518 | Ras-related protein Rab-1A | P62820 | 126 | 20.00 | 4 |
| 1519 | Ras-related protein Rab-1B | Q9H0U4 | 96 | 21.89 | 4 |
| 1520 | Ras-related protein Rab-21 | Q9UL25 | 45 | 11.61 | 2 |
| 1521 | Ras-related protein Rab-25 | P57735 | 52 | 8.20 | 2 |
| 1522 | Ras-related protein Rab-5B | P61020 | 92 | 10.23 | 3 |
| 1523 | Ras-related protein Rab-5C | P51148 | 62 | 10.19 | 3 |
| 1524 | Ras-related protein Rab-6A | P20340 | 85 | 11.54 | 3 |
| 1525 | Ras-related protein Rab-7a | P51149 | 341 | 37.68 | 7 |
| 1526 | Ras-related protein Ral-B | P11234 | 126 | 9.71 | 3 |
| 1527 | Ras-related protein R-Ras | P10301 | 54 | 9.63 | 2 |
| 1528 | RAS-responsive element-binding protein 1 | Q92766 | 42 | 1.15 | 3 |
| 1529 | Receptor-type tyrosine-protein phosphatase F | P10586 | 44 | 1.32 | 2 |
| 1530 | Receptor-type tyrosine-protein phosphatase gamma | P23470 | 55 | 1.38 | 3 |
| 1531 | Regulating synaptic membrane exocytosis protein 3 | Q9UJD0 | 41 | 9.74 | 2 |
| 1532 | Regulator of chromosome condensation | P18754 | 111 | 15.46 | 5 |
| 1533 | Regulator of differentiation 1 | O95758 | 110 | 7.93 | 5 |
| 1534 | Regulator of G-protein signaling 12 | O14924 | 64 | 2.03 | 3 |
| 1535 | Regulator of nonsense transcripts 1 | Q92900 | 395 | 13.46 | 9 |
| 1536 | Regulatory-associated protein of mTOR | Q8N122 | 49 | 1.20 | 3 |
| 1537 | Remodeling and spacing factor 1 | Q96T23 | 71 | 3.28 | 2 |
| 1538 | Replication factor C subunit 3 | P40938 | 182 | 16.57 | 5 |
| 1539 | Replication factor C subunit 4 | P35249 | 159 | 14.60 | 6 |
| 1540 | Replication protein A 70 kDa DNA-binding subunit | P27694 | 78 | 4.55 | 3 |
| 1541 | Reticulocalbin-1 | Q15293 | 40 | 6.34 | 2 |
| 1542 | Reticulon-2 | O75298 | 45 | 5.48 | 2 |
| 1543 | Reticulon-3 | O95197 | 92 | 5.10 | 2 |
| 1544 | Reticulon-4 receptor-like 1 | Q86UN2 | 40 | 4.73 | 2 |
| 1545 | Retinal dehydrogenase 1 | P00352 | 203 | 11.20 | 5 |
| 1546 | Retinal-specific ATP-binding cassette transporter | P78363 | 62 | 1.74 | 4 |
| 1547 | Retinoic acid-induced protein 1 | Q7Z5J4 | 56 | 1.94 | 4 |
| 1548 | Retinol-binding protein 3 | P10745 | 44 | 1.84 | 2 |
| 1549 | Rho GDP-dissociation inhibitor 1 | P52565 | 126 | 13.79 | 4 |
| 1550 | Rho GTPase-activating protein 20 | Q9P2F6 | 47 | 1.85 | 2 |
| 1551 | Rho GTPase-activating protein 7 | Q96QB1 | 50 | 2.09 | 2 |
| 1552 | Rho GTPase-activating protein RICH2 | Q17R89 | 51 | 2.57 | 2 |
| 1553 | Rho GTPase-activating protein SYDE2 | Q5VT97 | 45 | 1.70 | 2 |
| 1554 | Rho guanine nucleotide exchange factor 18 | Q6ZSZ5 | 41 | 1.81 | 2 |
| 1555 | Rho-associated protein kinase 2 | O75116 | 129 | 4.76 | 3 |
| 1556 | Rhophilin-2 | Q8IUC4 | 55 | 2.77 | 2 |
| 1557 | Ribonuclease inhibitor | P13489 | 233 | 15.43 | 6 |
| 1558 | Ribonucleases P/MRP protein subunit POP1 | Q99575 | 51 | 1.86 | 2 |
| 1559 | Ribonucleoside-diphosphate reductase large subunit | P23921 | 83 | 5.30 | 4 |
| 1560 | Ribonucleoside-diphosphate reductase subunit M2 | P31350 | 86 | 12.85 | 4 |
| 1561 | Ribosomal L1 domain-containing protein 1 | O76021 | 91 | 3.47 | 3 |
| 1562 | Ribosomal protein L10 | P27635 | 248 | 17.83 | 5 |
| 1563 | Ribosomal protein S6 kinase alpha-3 | P51812 | 46 | 3.92 | 2 |
| 1564 | Ribosomal RNA processing protein 1 homolog A | P56182 | 68 | 3.69 | 2 |
| 1565 | Ribosomal RNA-processing protein 7 homolog A | Q9Y3A4 | 44 | 4.02 | 2 |
| 1566 | Ribosome biogenesis protein BMS1 homolog | Q14692 | 47 | 1.64 | 2 |
| 1567 | Ribosome maturation protein SBDS | Q9Y3A5 | 89 | 10.44 | 3 |
| 1568 | Ribosome-binding protein 1 | Q9P2E9 | 682 | 18.32 | 8 |
| 1569 | RIMS-binding protein 2 | O15034 | 42 | 2.00 | 2 |
| 1570 | RNA polymerase II elongation factor ELL | P55199 | 55 | 6.19 | 2 |
| 1571 | RNA polymerase II elongation factor ELL2 | O00472 | 55 | 5.20 | 2 |
| 1572 | RNA polymerase II subunit A C-terminal domain phosphatase | Q9Y5B0 | 49 | 2.39 | 2 |
| 1573 | RNA-binding protein 10 | P98175 | 44 | 2.34 | 2 |
| 1574 | RNA-binding protein 12 | Q9NTZ6 | 76 | 3.35 | 3 |
| 1575 | RNA-binding protein 14 | Q96PK6 | 229 | 8.82 | 6 |
| 1576 | RNA-binding protein 25 | P49756 | 57 | 8.84 | 2 |
| 1577 | RNA-binding protein 28 | Q9NW13 | 49 | 3.03 | 2 |
| 1578 | RNA-binding protein 39 | Q14498 | 126 | 13.94 | 4 |
| 1579 | RNA-binding protein 4 | Q9BWF3 | 427 | 28.57 | 10 |
| 1580 | RNA-binding protein Raly | Q9UKM9 | 377 | 29.41 | 9 |
| 1581 | RNA-binding Raly-like protein | Q86SE5 | 86 | 6.87 | 2 |
| 1582 | Rootletin | Q5TZA2 | 72 | 1.51 | 3 |
| 1583 | rRNA 2'-O-methyltransferase fibrillarin | P22087 | 195 | 19.62 | 4 |
| 1584 | R-spondin-1 | Q2MKA7 | 47 | 11.02 | 2 |
| 1585 | RUN domain-containing protein 1 | Q96C34 | 64 | 9.14 | 3 |
| 1586 | Runt-related transcription factor 1 | Q01196 | 56 | 9.32 | 3 |
| 1587 | RuvB-like 1 | Q9Y265 | 164 | 11.92 | 4 |
| 1588 | RuvB-like 2 | Q9Y230 | 261 | 15.37 | 7 |
| 1589 | Ryanodine receptor 2 | Q92736 | 81 | 1.33 | 5 |
| 1590 | Ryanodine receptor 3 | Q15413 | 53 | 0.86 | 3 |
| 1591 | S-adenosylmethionine synthetase isoform type-2 | P31153 | 155 | 14.94 | 4 |
| 1592 | Scaffold attachment factor B1 | Q15424 | 242 | 11.69 | 3 |
| 1593 | Scaffold attachment factor B2 | Q14151 | 252 | 11.75 | 7 |
| 1594 | Scavenger receptor class F member 2 | Q96GP6 | 80 | 5.77 | 4 |
| 1595 | Scavenger receptor cysteine-rich type 1 protein M130 | Q86VB7 | 66 | 1.82 | 2 |
| 1596 | Secernin-1 | Q12765 | 186 | 10.87 | 6 |
| 1597 | Secernin-2 | Q96FV2 | 77 | 6.98 | 2 |
| 1598 | Secreted frizzled-related protein 4 | Q6FHJ7 | 42 | 7.45 | 2 |
| 1599 | Seizure protein 6 homolog | Q53EL9 | 63 | 2.76 | 3 |
| 1600 | Selenide, water dikinase 2 | Q99611 | 55 | 6.92 | 3 |
| 1601 | Selenoprotein P | P49908 | 43 | 7.61 | 2 |
| 1602 | Separin | Q14674 | 43 | 0.86 | 2 |
| 1603 | Sepiapterin reductase | P35270 | 122 | 13.13 | 2 |
| 1604 | Septin-2 | Q15019 | 233 | 18.47 | 7 |
| 1605 | Septin-7 | Q16181 | 95 | 10.05 | 4 |
| 1606 | Septin-8 | Q92599 | 115 | 6.02 | 4 |
| 1607 | Septin-9 | Q9UHD8 | 78 | 10.26 | 3 |
| 1608 | Serine hydroxymethyltransferase, cytosolic | P34896 | 93 | 5.32 | 3 |
| 1609 | Serine hydroxymethyltransferase, mitochondrial | P34897 | 517 | 20.63 | 10 |
| 1610 | Serine palmitoyltransferase 3 | Q9NUV7 | 45 | 9.35 | 3 |
| 1611 | Serine racemase | Q9GZT4 | 58 | 12.35 | 3 |
| 1612 | Serine/arginine repetitive matrix protein 2 | Q9UQ35 | 81 | 2.69 | 6 |
| 1613 | Serine/threonine kinase 31 | Q6PCD3 | 41 | 1.67 | 2 |
| 1614 | Serine/threonine kinase NLK | Q9UBE8 | 48 | 3.30 | 2 |
| 1615 | Serine/threonine-protein kinase 13 | Q9UQB9 | 43 | 9.48 | 2 |
| 1616 | Serine/threonine-protein kinase 25 | O00506 | 61 | 8.19 | 2 |
| 1617 | Serine/threonine-protein kinase 40 | Q8N2I9 | 41 | 5.52 | 2 |
| 1618 | Serine/threonine-protein kinase DCLK2 | Q8N568 | 52 | 3.79 | 2 |
| 1619 | Serine/threonine-protein kinase MRCK alpha | Q5VT25 | 53 | 1.34 | 2 |
| 1620 | Serine/threonine-protein kinase MRCK gamma | Q6DT37 | 56 | 4.63 | 3 |
| 1621 | Serine/threonine-protein kinase MST4 | Q9P289 | 64 | 8.93 | 3 |
| 1622 | Serine/threonine-protein kinase PLK3 | Q9H4B4 | 53 | 4.49 | 3 |
| 1623 | Serine/threonine-protein kinase receptor R3 | P37023 | 53 | 4.37 | 2 |
| 1624 | Serine/threonine-protein kinase SIK3 | Q9Y2K2 | 43 | 1.53 | 2 |
| 1625 | Serine/threonine-protein kinase SMG1 | Q96Q15 | 61 | 1.22 | 3 |
| 1626 | Serine/threonine-protein kinase SRPK1 | Q96SB4 | 64 | 3.82 | 3 |
| 1627 | Serine/threonine-protein kinase ULK1 | O75385 | 46 | 2.06 | 2 |
| 1628 | Serine/threonine-protein kinase WNK2 | Q9Y3S1 | 52 | 17.91 | 2 |
| 1629 | Serine/threonine-protein phosphatase 2A 55 kDa regulatory subunit B alpha isoform | P63151 | 112 | 5.59 | 3 |
| 1630 | Serine/threonine-protein phosphatase 2A regulatory subunit B'' subunit alpha | Q06190 | 46 | 2.61 | 3 |
| 1631 | Serine/threonine-protein phosphatase 6 | O00743 | 44 | 3.63 | 2 |
| 1632 | Serine/threonine-protein phosphatase PGAM5, mitochondrial | Q96HS1 | 161 | 12.94 | 3 |
| 1633 | Serine/threonine-protein phosphatase PP1-beta catalytic subunit | P62140 | 217 | 22.09 | 6 |
| 1634 | Serine/threonine-protein phosphatase PP1-gamma catalytic subunit | P36873 | 279 | 25.08 | 7 |
| 1635 | Serine-threonine kinase receptor-associated protein | Q9Y3F4 | 347 | 18.86 | 5 |
| 1636 | Serpin H1 | P50454 | 700 | 35.17 | 11 |
| 1637 | Serrate RNA effector molecule homolog | Q9BXP5 | 242 | 11.70 | 6 |
| 1638 | Serum albumin | P02768 | 200 | 7.22 | 5 |
| 1639 | S-formylglutathione hydrolase | P10768 | 251 | 14.54 | 3 |
| 1640 | SH2 domain-containing protein 3C | Q8N5H7 | 61 | 10.19 | 2 |
| 1641 | SH3 and multiple ankyrin repeat domains protein 1 | Q9Y566 | 47 | 0.97 | 2 |
| 1642 | SH3 domain-binding protein 5 | O60239 | 45 | 4.40 | 2 |
| 1643 | SH3 domain-containing guanine exchange factor | Q96DR7 | 59 | 1.61 | 2 |
| 1644 | Shootin-1 | A0MZ66 | 57 | 6.45 | 3 |
| 1645 | Short stature homeobox protein 2 | O60902 | 116 | 14.64 | 2 |
| 1646 | Short-chain specific acyl-CoA dehydrogenase, mitochondrial [Precursor] | P16219 | 80 | 5.34 | 2 |
| 1647 | Sialic acid synthase | Q9NR45 | 421 | 26.74 | 10 |
| 1648 | Sickle tail protein homolog | Q5T5P2 | 67 | 1.64 | 2 |
| 1649 | Sideroflexin-1 | Q9H9B4 | 422 | 22.12 | 8 |
| 1650 | Sigma non-opioid intracellular receptor 1 | Q99720 | 69 | 6.73 | 2 |
| 1651 | Signal recognition particle receptor subunit beta | Q9Y5M8 | 107 | 9.96 | 2 |
| 1652 | Signal transducer and activator of transcription 1-alpha/beta | P42224 | 170 | 9.87 | 6 |
| 1653 | Sister chromatid cohesion protein PDS5 homolog B | Q9NTI5 | 91 | 3.94 | 2 |
| 1654 | Slit homolog 3 protein | O75094 | 101 | 3.09 | 5 |
| 1655 | Small subunit processome component 20 homolog | O75691 | 49 | 1.11 | 3 |
| 1656 | Small ubiquitin-related modifier 1 | P63165 | 55 | 14.56 | 2 |
| 1657 | Smith-Magenis syndrome chromosomal region candidate gene 8 protein | Q8TEV9 | 50 | 2.15 | 2 |
| 1658 | Smoothelin | P53814 | 56 | 2.84 | 3 |
| 1659 | Sn1-specific diacylglycerol lipase alpha | Q9Y4D2 | 67 | 1.63 | 2 |
| 1660 | SNW domain-containing protein 1 | Q13573 | 111 | 6.72 | 3 |
| 1661 | Sodium channel protein type 1 subunit alpha | P35498 | 48 | 1.49 | 3 |
| 1662 | Sodium channel protein type 10 subunit alpha | Q9Y5Y9 | 52 | 1.53 | 3 |
| 1663 | Sodium/hydrogen exchanger 11 | Q5TAH2 | 83 | 1.69 | 3 |
| 1664 | Sodium/potassium-transporting ATPase alpha-3 chain | P13637 | 442 | 12.51 | 8 |
| 1665 | Sodium/potassium-transporting ATPase subunit alpha-1 | P05023 | 363 | 10.56 | 6 |
| 1666 | Sodium/potassium-transporting ATPase subunit beta-1 | P05026 | 55 | 10.56 | 3 |
| 1667 | Sodium-independent sulfate anion transporter | Q86WA9 | 60 | 7.10 | 3 |
| 1668 | Solute carrier family 17 member 9 | Q9BYT1 | 57 | 7.23 | 2 |
| 1669 | Solute carrier family 2, facilitated glucose transporter member 1 | P11166 | 93 | 3.68 | 3 |
| 1670 | Solute carrier family 2, facilitated glucose transporter member 14 | Q8TDB8 | 228 | 10.03 | 3 |
| 1671 | Solute carrier family 2, facilitated glucose transporter member 3 | P11169 | 212 | 10.03 | 4 |
| 1672 | Solute carrier family 2, member 1 | P11166 | 118 | 3.68 | 3 |
| 1673 | Sorbitol dehydrogenase | Q00796 | 107 | 12.08 | 5 |
| 1674 | Sorting nexin-5 | Q9Y5X3 | 126 | 13.83 | 5 |
| 1675 | Sorting nexin-6 | Q9UNH7 | 68 | 8.05 | 4 |
| 1676 | Spartin | Q8N0X7 | 66 | 2.48 | 2 |
| 1677 | Spectrin alpha chain, brain | Q13813 | 4138 | 31.03 | 70 |
| 1678 | Spectrin beta chain, brain 1 | Q01082 | 2413 | 25.06 | 43 |
| 1679 | Spectrin beta chain, brain 3 | Q9H254 | 88 | 2.50 | 4 |
| 1680 | Spectrin beta chain, brain 4 | Q9NRC6 | 60 | 0.95 | 4 |
| 1681 | Spectrin beta chain, erythrocyte | P11277 | 48 | 11.40 | 2 |
| 1682 | Spermatid perinuclear RNA-binding protein | Q96SI9 | 53 | 4.23 | 2 |
| 1683 | Spermatogenesis-associated protein 7 | Q9P0W8 | 49 | 4.76 | 3 |
| 1684 | Spermine synthase | P52788 | 154 | 12.30 | 5 |
| 1685 | Sperm-specific antigen 2 | P28290 | 46 | 1.66 | 2 |
| 1686 | Spliceosome RNA helicase BAT1 | Q13838 | 329 | 14.95 | 6 |
| 1687 | Splicing factor 3 subunit 1 | Q15459 | 47 | 2.40 | 2 |
| 1688 | Splicing factor 3B subunit 1 | O75533 | 122 | 4.98 | 3 |
| 1689 | Splicing factor 3B subunit 2 | Q13435 | 171 | 6.39 | 3 |
| 1690 | Splicing factor 3B subunit 3 | Q15393 | 548 | 14.30 | 10 |
| 1691 | Splicing factor U2AF 35 kDa subunit | Q01081 | 64 | 8.79 | 2 |
| 1692 | Splicing factor, arginine/serine-rich 1 | Q07955 | 468 | 41.70 | 12 |
| 1693 | Splicing factor, arginine/serine-rich 13A | O75494 | 59 | 9.16 | 2 |
| 1694 | Splicing factor, arginine/serine-rich 19 | Q9H7N4 | 47 | 1.68 | 2 |
| 1695 | Splicing factor, arginine/serine-rich 2 | Q01130 | 147 | 15.91 | 4 |
| 1696 | Splicing factor, arginine/serine-rich 5 | Q13243 | 161 | 16.56 | 5 |
| 1697 | Splicing factor, arginine/serine-rich 6 | Q13247 | 233 | 26.16 | 7 |
| 1698 | Splicing factor, arginine/serine-rich 7 | Q16629 | 126 | 33.58 | 5 |
| 1699 | Splicing factor, arginine/serine-rich 9 | Q13242 | 142 | 14.03 | 4 |
| 1700 | Splicing factor, proline- and glutamine-rich | P23246 | 633 | 20.98 | 12 |
| 1701 | SPS1/STE20-related protein kinase YSK4 | Q56UN5 | 72 | 2.80 | 4 |
| 1702 | SPX and EXS domain-containing protein 3 | Q54MJ9 | 65 | 4.18 | 4 |
| 1703 | Squalene synthetase | P37268 | 90 | 17.03 | 5 |
| 1704 | Squamous cell carcinoma antigen recognized by T-cells 3 | Q15020 | 139 | 7.89 | 2 |
| 1705 | Src substrate cortactin | Q14247 | 177 | 10.55 | 5 |
| 1706 | Stabilin-1 | Q9NY15 | 41 | 0.86 | 2 |
| 1707 | Stabilin-2 | Q8WWQ8 | 49 | 1.06 | 3 |
| 1708 | Staphylococcal nuclease domain-containing protein 1 | Q7KZF4 | 894 | 22.30 | 22 |
| 1709 | Stathmin ( Oncoprotein 18) | P16949 | 105 | 15.54 | 2 |
| 1710 | Sterile alpha and TIR motif-containing protein 1 | Q6SZW1 | 55 | 2.90 | 2 |
| 1711 | Sterol regulatory element-binding protein 1 | P36956 | 65 | 1.53 | 3 |
| 1712 | Sterol regulatory element-binding protein 2 | Q12772 | 45 | 2.01 | 2 |
| 1713 | Sterol-4-alpha-carboxylate 3-dehydrogenase, decarboxylating | Q15738 | 172 | 17.16 | 5 |
| 1714 | Stomatin-like protein 2 | Q9UJZ1 | 141 | 15.73 | 4 |
| 1715 | Stress-70 protein, mitochondrial | P38646 | 49 | 100.00 | 2 |
| 1716 | Stress-induced-phosphoprotein 1 | P31948 | 154 | 7.63 | 5 |
| 1717 | Striatin-4 | Q9NRL3 | 41 | 3.19 | 2 |
| 1718 | Structural maintenance of chromosomes | A6NHR9 | 102 | 3.88 | 3 |
| 1719 | Structural maintenance of chromosomes protein 1B | Q8NDV3 | 80 | 2.27 | 3 |
| 1720 | Structural maintenance of chromosomes protein 2 | O95347 | 99 | 5.13 | 2 |
| 1721 | Structural maintenance of chromosomes protein 3 | Q9UQE7 | 163 | 5.67 | 3 |
| 1722 | Structural maintenance of chromosomes protein 4 | Q9NTJ3 | 132 | 4.96 | 3 |
| 1723 | Succinate dehydrogenase [ubiquinone] flavoprotein subunit, mitochondrial | P31040 | 59 | 2.99 | 2 |
| 1724 | Succinate dehydrogenase [ubiquinone] iron-sulfur subunit, mitochondrial | P21912 | 53 | 6.07 | 2 |
| 1725 | Succinate-semialdehyde dehydrogenase, mitochondrial | P51649 | 179 | 5.61 | 4 |
| 1726 | Succinyl-CoA ligase [GDP-forming] subunit beta, mitochondrial | Q96I99 | 246 | 11.11 | 4 |
| 1727 | Sulfotransferase 1C2 | O00338 | 47 | 6.42 | 2 |
| 1728 | SUMO-activating enzyme subunit 2 | Q9UBT2 | 105 | 5.94 | 4 |
| 1729 | Superkiller viralicidic activity 2-like 2 | P42285 | 108 | 7.38 | 5 |
| 1730 | Superoxide dismutase [Mn], mitochondrial | P04179 | 111 | 13.96 | 3 |
| 1731 | Suppressor of G2 allele of SKP1 homolog | Q9Y2Z0 | 141 | 11.26 | 3 |
| 1732 | Sushi, von Willebrand factor type A, EGF and pentraxin domain-containing protein 1 | Q4LDE5 | 57 | 0.67 | 2 |
| 1733 | SWI/SNF-related matrix-associated actin-dependent regulator of chromatin subfamily C member 2 | Q8TAQ2 | 157 | 5.85 | 5 |
| 1734 | Symplekin | Q92797 | 40 | 0.86 | 2 |
| 1735 | Synaptic vesicle membrane protein VAT-1 homolog | Q99536 | 178 | 16.79 | 5 |
| 1736 | Synaptojanin-1 | O43426 | 42 | 1.90 | 2 |
| 1737 | Synaptonemal complex protein 1 | Q15431 | 62 | 3.60 | 4 |
| 1738 | Synaptotagmin-11 | Q9BT88 | 51 | 4.87 | 2 |
| 1739 | Synemin | O15061 | 65 | 3.01 | 4 |
| 1740 | Syntaxin-binding protein 2 | Q15833 | 107 | 8.47 | 3 |
| 1741 | Talin-1 | Q9Y490 | 619 | 9.29 | 13 |
| 1742 | TAR DNA-binding protein 43 | Q13148 | 197 | 9.42 | 3 |
| 1743 | TATA box-binding protein-like protein 2 | Q6SJ96 | 40 | 4.53 | 3 |
| 1744 | TATA-binding protein-associated factor 172 | O14981 | 747 | 16.58 | 17 |
| 1745 | TBC1 domain family member 1 | Q86TI0 | 48 | 1.89 | 2 |
| 1746 | TBC1 domain family member 12 | O60347 | 52 | 3.64 | 2 |
| 1747 | TBC1 domain family member 4 | O60343 | 181 | 5.54 | 4 |
| 1748 | T-cell receptor alpha chain C region | P01848 | 58 | 15.70 | 4 |
| 1749 | T-complex protein 1 subunit alpha | P17987 | 517 | 17.09 | 13 |
| 1750 | T-complex protein 1 subunit beta | P78371 | 457 | 20.79 | 10 |
| 1751 | T-complex protein 1 subunit delta | P50991 | 184 | 11.34 | 6 |
| 1752 | T-complex protein 1 subunit epsilon | Q4R6V2 | 182 | 10.91 | 6 |
| 1753 | T-complex protein 1 subunit eta | Q99832 | 131 | 7.37 | 4 |
| 1754 | T-complex protein 1 subunit gamma | P49368 | 599 | 22.39 | 14 |
| 1755 | T-complex protein 1 subunit theta | P50990 | 405 | 15.03 | 8 |
| 1756 | T-complex protein 1 subunit zeta | P40227 | 408 | 16.79 | 9 |
| 1757 | T-complex protein 10A homolog | Q12799 | 43 | 5.70 | 2 |
| 1758 | Telomere-associated protein RIF1 | Q5UIP0 | 43 | 0.85 | 2 |
| 1759 | Tenascin-X | P22105 | 50 | 1.02 | 3 |
| 1760 | Tensin-1 | Q9HBL0 | 48 | 2.25 | 2 |
| 1761 | Terminal uridylyltransferase 7 | Q5VYS8 | 45 | 1.27 | 2 |
| 1762 | Testicular haploid expressed gene protein | Q9P2T0 | 58 | 5.80 | 2 |
| 1763 | Testis-expressed sequence 13B protein | Q9BXU2 | 59 | 7.05 | 2 |
| 1764 | Testis-expressed sequence 15 protein | Q9BXT5 | 80 | 0.90 | 3 |
| 1765 | Testis-expressed sequence 2 protein | Q8IWB9 | 44 | 1.60 | 2 |
| 1766 | Testis-specific Y-encoded-like protein 3 | Q9H489 | 61 | 6.48 | 3 |
| 1767 | Testis-specific Y-encoded-like protein 5 | Q86VY4 | 61 | 3.71 | 2 |
| 1768 | Tether containing UBX domain for GLUT4 | Q9BZE9 | 44 | 3.25 | 2 |
| 1769 | Tetratricopeptide repeat protein 25 | Q96NG3 | 78 | 5.06 | 3 |
| 1770 | Tetratricopeptide repeat protein 28 | Q96AY4 | 43 | 1.09 | 3 |
| 1771 | THAP domain-containing protein 10 | Q9P2Z0 | 41 | 8.17 | 2 |
| 1772 | Thimet oligopeptidase | P52888 | 43 | 3.92 | 2 |
| 1773 | Thioredoxin | P10599 | 75 | 22.12 | 2 |
| 1774 | Thioredoxin domain-containing protein 5 | Q8NBS9 | 297 | 13.89 | 6 |
| 1775 | Thioredoxin-dependent peroxide reductase, mitochondrial | P30048 | 113 | 7.81 | 2 |
| 1776 | Thiosulfate sulfurtransferase | Q16762 | 142 | 10.81 | 3 |
| 1777 | THO complex subunit 3 | Q96J01 | 88 | 14.68 | 5 |
| 1778 | THO complex subunit 4 | Q86V81 | 220 | 15.23 | 3 |
| 1779 | Three prime repair exonuclease 2 | Q9BQ50 | 44 | 7.17 | 2 |
| 1780 | Threonyl-tRNA synthetase, cytoplasmic | P26639 | 332 | 12.31 | 9 |
| 1781 | Thrombospondin type-1 domain-containing protein 7A | Q9UPZ6 | 99 | 3.60 | 6 |
| 1782 | Thyroglobulin | P01266 | 50 | 1.12 | 2 |
| 1783 | Thyroid hormone receptor-associated protein 3 | Q9Y2W1 | 143 | 6.70 | 3 |
| 1784 | Thyroid peroxidase [Precursor] | P07202 | 43 | 2.57 | 2 |
| 1785 | Tight junction protein ZO-1 | Q07157 | 306 | 8.58 | 7 |
| 1786 | Tight junction protein ZO-2 | Q9UDY2 | 83 | 4.37 | 2 |
| 1787 | Tight junction protein ZO-3 | O95049 | 42 | 2.67 | 2 |
| 1788 | Titin | Q8WZ42 | 216 | 0.78 | 23 |
| 1789 | Torsin-1A-interacting protein 1 | Q5JTV8 | 48 | 6.00 | 3 |
| 1790 | TRAF2 and NCK-interacting protein kinase | Q9UKE5 | 66 | 2.65 | 3 |
| 1791 | Trafficking protein particle complex subunit 9 | Q96Q05 | 49 | 16.67 | 2 |
| 1792 | Transaldolase | P37837 | 577 | 30.65 | 14 |
| 1793 | Transcription activator BRG1 | P51532 | 207 | 5.24 | 7 |
| 1794 | Transcription elongation factor A protein 1 | P23193 | 97 | 9.63 | 3 |
| 1795 | Transcription elongation factor SPT5 | O00267 | 204 | 7.08 | 7 |
| 1796 | Transcription elongation factor SPT6 | Q7KZ85 | 92 | 3.13 | 3 |
| 1797 | Transcription elongation regulator 1 | O14776 | 82 | 3.53 | 3 |
| 1798 | Transcription factor 15 | Q12870 | 51 | 12.56 | 2 |
| 1799 | Transcription factor A, mitochondrial | Q00059 | 166 | 18.70 | 5 |
| 1800 | Transcription factor BTF3 | P20290 | 51 | 5.06 | 2 |
| 1801 | Transcription factor E2F2 | Q14209 | 41 | 4.81 | 2 |
| 1802 | Transcription factor HIVEP2 | P31629 | 48 | 0.60 | 2 |
| 1803 | Transcription factor SOX-30 | O94993 | 57 | 4.78 | 3 |
| 1804 | Transcription initiation factor TFIID subunit 1-like | Q8IZX4 | 69 | 1.70 | 3 |
| 1805 | Transcription initiation factor TFIID subunit 4 | O00268 | 45 | 2.85 | 2 |
| 1806 | Transcription intermediary factor 1-beta | Q13263 | 696 | 19.30 | 18 |
| 1807 | Transcriptional repressor CTCF | P49711 | 94 | 5.91 | 2 |
| 1808 | Transducin beta-like protein 2 | Q9Y4P3 | 56 | 4.47 | 2 |
| 1809 | Transducin beta-like protein 3 | Q12788 | 115 | 5.20 | 4 |
| 1810 | Transferrin receptor protein 1 | P02786 | 687 | 21.45 | 13 |
| 1811 | Transformation/transcription domain-associated protein | Q9Y4A5 | 94 | 1.62 | 5 |
| 1812 | Transformer-2 protein homolog beta | P62995 | 300 | 25.00 | 7 |
| 1813 | Transforming acidic coiled-coil-containing protein 1 | O75410 | 86 | 5.09 | 4 |
| 1814 | Transforming growth factor beta-3 | P10600 | 59 | 9.39 | 3 |
| 1815 | Transgelin | Q01995 | 160 | 24.00 | 7 |
| 1816 | Transgelin-2 | P37802 | 79 | 28.79 | 5 |
| 1817 | Transient receptor potential cation channel subfamily M member 5 | Q9NZQ8 | 48 | 1.81 | 2 |
| 1818 | Transitional endoplasmic reticulum ATPase | P55072 | 297 | 9.19 | 10 |
| 1819 | Transketolase | P29401 | 839 | 28.73 | 19 |
| 1820 | Translation initiation factor IF-3, mitochondrial | Q9H2K0 | 44 | 6.12 | 2 |
| 1821 | Translational activator GCN1 | Q92616 | 796 | 9.59 | 21 |
| 1822 | Translationally-controlled tumor protein | P13693 | 148 | 17.02 | 5 |
| 1823 | Translin | Q15631 | 108 | 12.72 | 4 |
| 1824 | Transmembrane 9 superfamily member 2 | Q99805 | 42 | 1.66 | 2 |
| 1825 | Transmembrane 9 superfamily member 3 | Q9HD45 | 54 | 4.24 | 2 |
| 1826 | Transmembrane and coiled-coil domain-containing protein C6orf129 | Q9P0B6 | 69 | 21.93 | 3 |
| 1827 | Transmembrane and TPR repeat-containing protein 2 | Q8N394 | 70 | 3.71 | 3 |
| 1828 | Transmembrane channel-like protein 6 | Q7Z403 | 45 | 5.95 | 2 |
| 1829 | Transmembrane emp24 domain-containing protein 10 | P49755 | 84 | 8.44 | 2 |
| 1830 | Transmembrane emp24 domain-containing protein 5 | Q9Y3A6 | 77 | 9.30 | 2 |
| 1831 | Transmembrane emp24 domain-containing protein 7 | Q9Y3B3 | 45 | 12.69 | 2 |
| 1832 | Transmembrane protease, serine 13 | Q9BYE2 | 58 | 4.30 | 2 |
| 1833 | Transmembrane protein 97 | Q5BJF2 | 109 | 13.07 | 3 |
| 1834 | Transportin-2 | O14787 | 97 | 3.64 | 3 |
| 1835 | Treacle protein | Q13428 | 236 | 9.68 | 6 |
| 1836 | Triadin | Q13061 | 47 | 4.12 | 2 |
| 1837 | Tricarboxylate transport protein, mitochondrial | P53007 | 134 | 11.95 | 4 |
| 1838 | Trichoplein keratin filament-binding protein | Q9BT92 | 52 | 4.22 | 2 |
| 1839 | Trifunctional enzyme subunit alpha, mitochondrial | P40939 | 64 | 5.24 | 3 |
| 1840 | Trifunctional purine biosynthetic protein adenosine-3 | P22102 | 697 | 19.41 | 16 |
| 1841 | TRIO and F-actin-binding protein | Q9H2D6 | 68 | 2.55 | 5 |
| 1842 | Triosephosphate isomerase | P60174 | 894 | 41.53 | 11 |
| 1843 | Triple functional domain protein | O75962 | 52 | 2.00 | 3 |
| 1844 | tRNA (cytosine-5-)-methyltransferase NSUN2 | Q08J23 | 126 | 5.60 | 4 |
| 1845 | tRNA (guanine-N(7)-)-methyltransferase subunit WDR4 | P57081 | 65 | 7.52 | 3 |
| 1846 | tRNA dimethylallyltransferase, mitochondrial | Q9H3H1 | 49 | 6.07 | 2 |
| 1847 | tRNA pseudouridine synthase A | Q9Y606 | 81 | 10.03 | 4 |
| 1848 | tRNA wybutosine-synthesizing protein 2 homolog | Q53H54 | 40 | 4.53 | 2 |
| 1849 | Tropomyosin alpha-3 chain | P06753 | 752 | 54.03 | 15 |
| 1850 | Tropomyosin alpha-4 chain | P67936 | 500 | 36.44 | 9 |
| 1851 | Tropomyosin beta chain | P07951 | 202 | 18.31 | 5 |
| 1852 | TSC22 domain family protein 3 | Q99576 | 53 | 22.52 | 2 |
| 1853 | Tubby-related protein 1 | O00294 | 59 | 2.95 | 2 |
| 1854 | Tubulin alpha chain | P50719 | 276 | 15.08 | 5 |
| 1855 | Tubulin alpha-1B chain | P68363 | 920 | 40.58 | 17 |
| 1856 | Tubulin alpha-8 chain | Q9NY65 | 428 | 25.39 | 8 |
| 1857 | Tubulin beta chain | P07437 | 831 | 29.05 | 29 |
| 1858 | Tubulin beta-2A chain | Q13885 | 1097 | 39.10 | 29 |
| 1859 | Tubulin beta-2C chain | P68371 | 1354 | 44.49 | 36 |
| 1860 | Tubulin beta-3 chain | Q13509 | 906 | 29.56 | 25 |
| 1861 | Tubulin beta-6 chain | Q9BUF5 | 387 | 15.92 | 13 |
| 1862 | Tubulin polyglutamylase TTLL7 | Q6ZT98 | 57 | 2.59 | 2 |
| 1863 | Tubulin-folding cofactor B | Q99426 | 303 | 26.42 | 5 |
| 1864 | Tubulin-specific chaperone A | O75347 | 120 | 18.69 | 2 |
| 1865 | Tudor domain-containing protein 1 | Q9BXT4 | 41 | 1.44 | 2 |
| 1866 | Tudor domain-containing protein 6 | O60522 | 47 | 0.81 | 2 |
| 1867 | Tumor necrosis factor, alpha-induced protein 2 | Q03169 | 45 | 5.50 | 2 |
| 1868 | Tumor necrosis factor, alpha-induced protein 8-like protein 3 | Q5GJ75 | 97 | 7.76 | 5 |
| 1869 | Tumor protein 63 | Q9H3D4 | 40 | 6.39 | 2 |
| 1870 | Tumor protein D54 | O43399 | 69 | 15.31 | 3 |
| 1871 | Tumor suppressor p53-binding protein 1 | Q12888 | 40 | 1.16 | 2 |
| 1872 | Twinfilin-1 | Q12792 | 138 | 8.97 | 3 |
| 1873 | Twinfilin-2 | Q6IBS0 | 125 | 16.91 | 4 |
| 1874 | Type I iodothyronine deiodinase | P49895 | 43 | 12.05 | 2 |
| 1875 | Tyrosine-protein kinase BLK | P51451 | 62 | 2.98 | 2 |
| 1876 | Tyrosine-protein kinase HCK | P08631 | 79 | 3.43 | 2 |
| 1877 | Tyrosine-protein kinase ITK/TSK | Q08881 | 41 | 5.32 | 2 |
| 1878 | Tyrosine-protein kinase JAK3 | P52333 | 57 | 3.74 | 3 |
| 1879 | Tyrosine-protein kinase SYK | P43405 | 42 | 8.66 | 2 |
| 1880 | Tyrosine-protein phosphatase non-receptor type 13 | Q12923 | 56 | 1.69 | 3 |
| 1881 | Tyrosine-protein phosphatase non-receptor type 21 | Q16825 | 48 | 2.47 | 2 |
| 1882 | Tyrosyl-DNA phosphodiesterase 1 | Q9NUW8 | 45 | 5.70 | 2 |
| 1883 | Tyrosyl-tRNA synthetase, cytoplasmic | P54577 | 45 | 3.61 | 2 |
| 1884 | U1 small nuclear ribonucleoprotein 70 kDa | P08621 | 169 | 12.13 | 6 |
| 1885 | U2 small nuclear ribonucleoprotein A' | P09661 | 255 | 25.49 | 7 |
| 1886 | U2-associated protein SR140 | O15042 | 98 | 8.06 | 2 |
| 1887 | U3 small nucleolar RNA-associated protein 15 homolog | Q8TED0 | 59 | 5.21 | 2 |
| 1888 | U3 small nucleolar RNA-associated protein 18 homolog | Q9Y5J1 | 75 | 8.09 | 3 |
| 1889 | U3 small nucleolar RNA-interacting protein 2 | O43818 | 94 | 6.74 | 4 |
| 1890 | U4/U6.U5 tri-snRNP-associated protein 1 | O43290 | 93 | 2.38 | 4 |
| 1891 | U5 small nuclear ribonucleoprotein 200 kDa helicase | O75643 | 921 | 11.19 | 19 |
| 1892 | Ubiquitin | P62988 | 452 | 69.74 | 7 |
| 1893 | Ubiquitin associated protein 2-like | Q14157 | 96 | 8.57 | 2 |
| 1894 | Ubiquitin carboxyl-terminal hydrolase 14 | P54578 | 118 | 6.09 | 3 |
| 1895 | Ubiquitin carboxyl-terminal hydrolase 20 | Q9Y2K6 | 44 | 3.18 | 2 |
| 1896 | Ubiquitin carboxyl-terminal hydrolase 24 | Q9UPU5 | 51 | 1.23 | 3 |
| 1897 | Ubiquitin carboxyl-terminal hydrolase 47 | Q96K76 | 40 | 1.31 | 2 |
| 1898 | Ubiquitin carboxyl-terminal hydrolase 5 | P45974 | 142 | 5.27 | 4 |
| 1899 | Ubiquitin carboxyl-terminal hydrolase 6 | P35125 | 49 | 9.17 | 3 |
| 1900 | Ubiquitin conjugation factor E4 B | O95155 | 63 | 2.07 | 3 |
| 1901 | Ubiquitin fusion degradation protein 1 homolog | Q92890 | 161 | 18.66 | 5 |
| 1902 | Ubiquitin-conjugating enzyme E2 Q2 | Q8WVN8 | 45 | 4.27 | 2 |
| 1903 | Ubiquitin-conjugating enzyme E2 variant 1 | Q13404 | 154 | 32.04 | 4 |
| 1904 | Ubiquitin-conjugating enzyme E2 variant 2 | Q15819 | 79 | 11.81 | 2 |
| 1905 | Ubiquitin-conjugating enzyme E2 Z | Q9H832 | 48 | 9.13 | 2 |
| 1906 | Ubiquitin-like modifier-activating enzyme 1 | P22314 | 1065 | 20.70 | 21 |
| 1907 | Ubiquitin-like modifier-activating enzyme 5 | Q9GZZ9 | 89 | 7.18 | 3 |
| 1908 | UBX domain-containing protein 1 | Q04323 | 80 | 9.62 | 3 |
| 1909 | UDP-GalNAc:beta-1,3-N-acetylgalactosaminyltransferase 2 | Q8NCR0 | 48 | 6.02 | 2 |
| 1910 | UDP-glucose 6-dehydrogenase | O60701 | 72 | 4.66 | 2 |
| 1911 | UDP-glucose:glycoprotein glucosyltransferase 1 | Q9NYU2 | 576 | 10.80 | 12 |
| 1912 | UDP-glucuronosyltransferase 1-6 | P19224 | 132 | 8.83 | 4 |
| 1913 | UDP-glucuronosyltransferase 2B15 | P54855 | 44 | 3.82 | 2 |
| 1914 | UMP-CMP kinase 2, mitochondrial | Q5EBM0 | 44 | 4.99 | 2 |
| 1915 | Uncharacterized protein C11orf61 | Q6P1R3 | 44 | 7.16 | 2 |
| 1916 | Uncharacterized protein C12orf40 | Q86WS4 | 67 | 5.70 | 3 |
| 1917 | Uncharacterized protein C14orf166B | Q0VAA2 | 62 | 5.59 | 3 |
| 1918 | Uncharacterized protein C14orf81 | Q96MM1 | 63 | 15.47 | 3 |
| 1919 | Uncharacterized protein C15orf39 | Q6ZRI6 | 50 | 6.35 | 2 |
| 1920 | Uncharacterized protein C16orf45 | Q96MC5 | 50 | 2.51 | 2 |
| 1921 | Uncharacterized protein C17orf55 | Q8N8I6 | 44 | 7.20 | 2 |
| 1922 | Uncharacterized protein C19orf44 | Q9H6X5 | 45 | 3.40 | 2 |
| 1923 | Uncharacterized protein C19orf60 | Q96EN9 | 54 | 7.78 | 2 |
| 1924 | Uncharacterized protein C1orf173 | Q5RHP9 | 47 | 1.90 | 2 |
| 1925 | Uncharacterized protein C1orf222 | Q69YW0 | 46 | 5.25 | 2 |
| 1926 | Uncharacterized protein C1orf77 | Q9Y3Y2 | 100 | 10.31 | 5 |
| 1927 | Uncharacterized protein C20orf117 | O94964 | 48 | 1.34 | 2 |
| 1928 | Uncharacterized protein C22orf34 | Q6ZV56 | 73 | 27.15 | 3 |
| 1929 | Uncharacterized protein C2orf16 | Q68DN1 | 59 | 1.61 | 3 |
| 1930 | Uncharacterized protein C4orf14 | Q8NC60 | 40 | 2.58 | 2 |
| 1931 | Uncharacterized protein C4orf17 | Q53FE4 | 44 | 16.43 | 2 |
| 1932 | Uncharacterized protein C6orf10 | Q5SRN2 | 46 | 3.37 | 2 |
| 1933 | Uncharacterized protein C6orf167 | Q6ZRQ5 | 54 | 2.02 | 2 |
| 1934 | Uncharacterized protein C7orf43 | Q8WVR3 | 44 | 2.93 | 2 |
| 1935 | Uncharacterized protein C7orf51 | Q6ZVC0 | 52 | 3.09 | 2 |
| 1936 | Uncharacterized protein C9orf131 | Q5VYM1 | 55 | 1.67 | 2 |
| 1937 | Uncharacterized protein C9orf4 | Q9P0K9 | 70 | 7.85 | 3 |
| 1938 | Uncharacterized protein FLJ46347 | Q6ZRH9 | 70 | 7.17 | 3 |
| 1939 | Uncharacterized protein HSD47 | Q6ZVN6 | 41 | 5.88 | 2 |
| 1940 | Uncharacterized protein KIAA0240 | Q6AI39 | 70 | 3.56 | 3 |
| 1941 | Uncharacterized protein KIAA0319 | Q5VV43 | 43 | 1.98 | 2 |
| 1942 | Uncharacterized protein KIAA0528 | Q86YS7 | 70 | 18.90 | 4 |
| 1943 | Uncharacterized protein KIAA0562 | O60308 | 61 | 2.16 | 2 |
| 1944 | Uncharacterized protein KIAA0564 | A3KMH1 | 41 | 7.81 | 2 |
| 1945 | Uncharacterized protein KIAA0802 | Q9Y4B5 | 76 | 1.77 | 3 |
| 1946 | Uncharacterized protein KIAA0819 | O94909 | 52 | 2.83 | 2 |
| 1947 | Uncharacterized protein KIAA1107 | Q9UPP5 | 50 | 2.03 | 2 |
| 1948 | Uncharacterized protein KIAA1671 | Q9BY89 | 44 | 29.69 | 2 |
| 1949 | Uncharacterized protein KIAA1683 | Q9H0B3 | 45 | 1.76 | 2 |
| 1950 | Uncharacterized protein KIAA1841 | Q6NSI8 | 50 | 2.80 | 2 |
| 1951 | UPF0027 protein C22orf28 | Q9Y3I0 | 102 | 6.14 | 3 |
| 1952 | UPF0160 protein MYG1, mitochondrial | Q9HB07 | 156 | 16.49 | 7 |
| 1953 | UPF0366 protein C11orf67 | Q9H7C9 | 109 | 23.77 | 2 |
| 1954 | UPF0399 protein C6orf153 | Q96EU6 | 55 | 8.49 | 2 |
| 1955 | UPF0546 membrane protein C1orf91 | Q8WY98 | 40 | 12.22 | 2 |
| 1956 | UPF0670 protein C8orf55 | Q8WUY1 | 53 | 9.13 | 2 |
| 1957 | Uridine 5'-monophosphate synthase | P11172 | 216 | 11.46 | 5 |
| 1958 | Uridine-cytidine kinase 2 | Q9BZX2 | 81 | 6.90 | 2 |
| 1959 | UTP--glucose-1-phosphate uridylyltransferase | Q16851 | 308 | 15.58 | 8 |
| 1960 | Utrophin | P46939 | 70 | 1.02 | 3 |
| 1961 | Vacuolar ATP synthase catalytic subunit A | P38606 | 64 | 2.59 | 2 |
| 1962 | Vacuolar ATP synthase subunit B, brain isoform | P21281 | 56 | 5.09 | 3 |
| 1963 | Vacuolar protein sorting-associated protein 13A | Q96RL7 | 95 | 1.83 | 4 |
| 1964 | Vacuolar protein sorting-associated protein 35 | Q96QK1 | 103 | 4.63 | 3 |
| 1965 | Vacuolar protein sorting-associated protein 72 homolog | Q15906 | 53 | 4.95 | 2 |
| 1966 | Vacuolar protein sorting-associated protein VTA1 homolog | Q9NP79 | 79 | 14.33 | 4 |
| 1967 | Vacuolar proton pump subunit C 1 | P21283 | 69 | 9.45 | 4 |
| 1968 | Vacuolar proton pump subunit E 1 | P36543 | 51 | 9.73 | 3 |
| 1969 | Valyl-tRNA synthetase | P26640 | 464 | 9.41 | 10 |
| 1970 | Vascular cell adhesion protein 1 | P19320 | 44 | 2.30 | 2 |
| 1971 | Vascular non-inflammatory molecule 3 | Q9NY84 | 49 | 6.79 | 2 |
| 1972 | Vasodilator-stimulated phosphoprotein | P50552 | 59 | 5.80 | 2 |
| 1973 | Very long-chain specific acyl-CoA dehydrogenase, mitochondrial | P49748 | 107 | 4.43 | 3 |
| 1974 | Very low density lipoprotein receptor | P98155 | 56 | 2.72 | 2 |
| 1975 | Vesicle transport through interaction with t-SNAREs homolog 1A | Q96AJ9 | 42 | 4.93 | 2 |
| 1976 | Vesicle-associated membrane protein-associated protein A | Q9P0L0 | 314 | 23.97 | 5 |
| 1977 | Vesicle-associated membrane protein-associated protein B/C | O95292 | 132 | 16.12 | 5 |
| 1978 | Vesicular glutamate transporter 2 | Q9P2U8 | 41 | 3.44 | 2 |
| 1979 | Vesicular integral-membrane protein VIP36 | Q12907 | 128 | 7.02 | 3 |
| 1980 | Vigilin | Q00341 | 617 | 13.41 | 12 |
| 1981 | Villin-1 | P09327 | 732 | 17.53 | 13 |
| 1982 | Vinculin | P18206 | 375 | 16.24 | 13 |
| 1983 | Vinexin | O60504 | 47 | 3.93 | 2 |
| 1984 | Visual system homeobox 2 | P58304 | 47 | 6.09 | 2 |
| 1985 | Voltage-dependent anion-selective channel protein 1 | P21796 | 692 | 41.13 | 9 |
| 1986 | Voltage-dependent anion-selective channel protein 2 | P45880 | 282 | 22.70 | 5 |
| 1987 | Voltage-dependent L-type calcium channel subunit alpha-1C | Q13936 | 40 | 1.13 | 2 |
| 1988 | Voltage-dependent L-type calcium channel subunit alpha-1F | O60840 | 47 | 2.80 | 2 |
| 1989 | Voltage-dependent L-type calcium channel subunit beta-2 | Q08289 | 44 | 3.14 | 2 |
| 1990 | Voltage-dependent N-type calcium channel subunit alpha-1B | Q00975 | 53 | 1.28 | 3 |
| 1991 | von Willebrand factor | P04275 | 49 | 3.89 | 2 |
| 1992 | von Willebrand factor A domain containing 5B2 | Q8N398 | 45 | 3.16 | 2 |
| 1993 | V-type proton ATPase subunit H | Q9UI12 | 54 | 4.65 | 2 |
| 1994 | Wall-associated protein | Q07833 | 65 | 1.76 | 3 |
| 1995 | WD repeat-containing protein 1 | O75083 | 149 | 6.28 | 5 |
| 1996 | WD repeat-containing protein 33 | Q9C0J8 | 121 | 3.37 | 3 |
| 1997 | WD repeat-containing protein 36 | Q8NI36 | 88 | 3.69 | 3 |
| 1998 | WD repeat-containing protein 60 | Q8WVS4 | 49 | 2.63 | 2 |
| 1999 | WD repeat-containing protein 65 | Q96MR6 | 45 | 3.44 | 2 |
| 2000 | WD repeat-containing protein 67 | Q96DN5 | 52 | 2.25 | 3 |
| 2001 | WD repeat-containing protein 74 | Q6RFH5 | 49 | 7.65 | 2 |
| 2002 | WD repeat-containing protein 90 | Q96KV7 | 64 | 4.43 | 4 |
| 2003 | Xin actin-binding repeat-containing protein 2 | A4UGR9 | 66 | 1.26 | 4 |
| 2004 | X-ray radiation resistance-associated protein 1 | Q6P2D8 | 56 | 3.96 | 3 |
| 2005 | Y-box-binding protein 2 | Q9Y2T7 | 56 | 8.52 | 3 |
| 2006 | YTH domain-containing protein 1 | Q96MU7 | 54 | 3.19 | 2 |
| 2007 | Zinc finger and SCAN domain-containing protein 22 | P10073 | 40 | 2.04 | 2 |
| 2008 | Zinc finger BED domain-containing protein 5 | Q49AG3 | 40 | 3.75 | 2 |
| 2009 | Zinc finger CCCH domain-containing protein 18 | Q86VM9 | 102 | 5.14 | 2 |
| 2010 | Zinc finger CCCH domain-containing protein 3 | Q8IXZ2 | 52 | 3.48 | 2 |
| 2011 | Zinc finger CCCH domain-containing protein 6 | P61129 | 61 | 3.20 | 3 |
| 2012 | Zinc finger CCHC domain-containing protein 2 | Q9C0B9 | 48 | 5.52 | 2 |
| 2013 | Zinc finger E-box-binding homeobox 1 | P37275 | 61 | 3.02 | 3 |
| 2014 | Zinc finger homeobox protein 3 | Q15911 | 48 | 0.54 | 2 |
| 2015 | Zinc finger homeobox protein 4 | Q86UP3 | 113 | 1.28 | 5 |
| 2016 | Zinc finger MYM-type protein 2 | Q9UBW7 | 51 | 1.81 | 2 |
| 2017 | Zinc finger MYND domain-containing protein 11 | Q15326 | 42 | 2.64 | 2 |
| 2018 | Zinc finger protein 229 | Q9UJW7 | 60 | 7.14 | 2 |
| 2019 | Zinc finger protein 275 | Q9NSD4 | 45 | 3.93 | 2 |
| 2020 | Zinc finger protein 292 | O60281 | 44 | 0.85 | 2 |
| 2021 | Zinc finger protein 324A | O75467 | 45 | 9.09 | 2 |
| 2022 | Zinc finger protein 324B | Q6AW86 | 45 | 3.86 | 2 |
| 2023 | Zinc finger protein 40 | P15822 | 106 | 2.50 | 6 |
| 2024 | Zinc finger protein 462 | Q96JM2 | 69 | 4.11 | 5 |
| 2025 | Zinc finger protein 469 | Q96JG9 | 62 | 1.46 | 3 |
| 2026 | Zinc finger protein 507 | Q8TCN5 | 52 | 2.00 | 2 |
| 2027 | Zinc finger protein 516 | Q92618 | 76 | 2.32 | 2 |
| 2028 | Zinc finger protein 554 | Q86TJ5 | 72 | 5.40 | 5 |
| 2029 | Zinc finger protein 793 | Q6ZN11 | 52 | 8.85 | 2 |
| 2030 | Zinc finger protein 8 | P17098 | 42 | 3.13 | 2 |
| 2031 | Zinc finger protein 829 | Q3KNS6 | 45 | 7.26 | 2 |
| 2032 | Zinc finger protein 837 | Q96EG3 | 44 | 6.27 | 3 |
| 2033 | Zinc finger protein 845 | Q96IR2 | 43 | 2.67 | 2 |
| 2034 | Zinc finger protein castor homolog 1 | Q86V15 | 69 | 2.57 | 3 |
| 2035 | Zinc finger protein ENSP00000375192 | Q8WTZ3 | 93 | 4.63 | 5 |
| 2036 | Zinc finger protein GLIS1 | Q8NBF1 | 46 | 3.87 | 3 |
| 2037 | Zinc finger protein GLIS3 | Q8NEA6 | 41 | 2.15 | 3 |
| 2038 | Zinc finger protein with KRAB and SCAN domains 5 | Q9Y2L8 | 52 | 2.03 | 2 |
| 2039 | Zinc finger protein ZFPM2 | Q8WW38 | 54 | 2.35 | 3 |
| 2040 | Zinc finger X-linked protein ZXDA | P98168 | 48 | 3.50 | 2 |
| 2041 | Zinc phosphodiesterase ELAC protein 2 | Q9BQ52 | 50 | 5.51 | 3 |
| 2042 | Zygote arrest protein 1 | Q86SH2 | 53 | 6.60 | 2 |
|  |  |  |  |  |  |
