# Supplementary material for: Impact of clostridial glucosylating toxins on the proteome of colonic cells determined by isotope-coded protein labeling and LC-MALDI
Source: Proteome Sci. 2011 Aug 17;9:48. doi: 10.1186/1477-5956-9-48 (PMC3176154; doi:10.1186/1477-5956-9-48)
Supplement: Additional file 2 — A summary of regulated proteins in Caco-2 cells after treatment with rTcdA wt or mutant rTcdA. [file 1477-5956-9-48-S2.DOCX]

**Additional file 2**

**A summary of regulated proteins in Caco-2 cells after treatment with rTcdA wt or mutant rTcdA**

| **After treatment with mutant rTcdA, 5 h** | | | | | | | | | | | | | | | | | |
| --- | --- | --- | --- | --- | --- | --- | --- | --- | --- | --- | --- | --- | --- | --- | --- | --- | --- |
| **Nr.** | | **Protein name** | | **Accession number** | **Gene name** | **Sequence coverage [%]** | | | **Mascot score** | | | **Peptide sequence** | | | | **Subcellular location** | |
| 1 | | Acetyl-CoA acetyltransferase, cytosolic variant | | Q9BWD1 | ACAT 2 | 19.8 | | | 173 | | | \| GLIEVKTDEFPR \| \| --- \| \| EDQDKVAVLSQNR  SKMNAGSDPVVIVSAAR  ATVAPEDVSEVIFGHVLAAGCGQNPVR  ILVTLLHTLER \| | | | | cytoplasm | |
| 2 | | Alpha-enolase | | P06733 | ENO1 | 12.0 | | | 291 | | | NFRNPLAK  LAKYNQLLR  AAVPSGASTGIYEALELR  GNPTVEVDLFTSKGLFR | | | | cytoplasm | |
| 3 | | Annexin A3 | | P12429 | ANXA3 | 14.3 | | | 154 | | | NTPAFLAER  KALLTLADGR  ALKGIGTDEFTLNR  SFPQLKLTFDEYR | | | | phagocytic vesicle membrane | |
| 4 | | Glucosamine--fructose-6-phosphate aminotransferase | | Q06210 | GFPT1 | 9.74 | | | 209 | | | DHTYAKCQNALQQVVAR  EILETLIKGLQR  GYDVDFPR  SEHKLSTDHIPILYR  VIFLEDDDVAAVVDGR | | | | cytoplasm | |
| 5 | | Macrophage migration inhibitory factor | | P14174 | MIF | 21.1 | | | 142 | | | PMFIVNTNVPR  SYSKLLCGLLAER | | | | secreted | |
| 6 | | Peroxiredoxin-1 | | Q06830 | PRDX2 | 28.1 | | | 228 | | | SVDETLR  QITVNDLPVGR  TIAQDYGVLKADEGISFR | | | | cytoplasm | |
| 7 | | Peroxiredoxin-2 | | P32119 | PRDX1 | 29.4 | | | 254 | | | GLFIIDGKGVLR  KEGGLGPLNIPLLADVTR  LSEDYGVLKTDEGIAYR | | | | cytoplasm | |
| **After treatment with rTcdA wt, 5 h** | | | | | | | | | | | | | | | | | |
| **Nr.** | | **Protein name** | **Accession number** | **Gene name** | | | **Sequence coverage [%]** | | | **Mascot score** | | | **Peptide sequence** | | **Subcellular location** | | |
| 1 | | 40S ribosomal protein S16 | P62249 | RPS16 | | | 23.3 | | | 141 | | | YQKSYR  VKGGGHVAQIYAIR  PSKGPLQSVQVFGR | | cytosolic small ribosomal subunit | | |
| 2 | | 40S ribosomal protein S17 | P08708 | RPS17 | | | 22.2 | | | 110 | | | VIIEKYYTR  LGNDFHTNKR  GISIKLQEEER | | cytosolic small ribosomal subunit | | |
| 3 | | 40S ribosomal protein S3 | P23396 | RPS3 | | | 29.6 | | | 232 | | | ELTAVVQKR  YKLLGGLAVR  TQNVLGEKGR  GLCAIAQAESLR  ELAEDGYSGVEVR  FGFPEGSVELYAEKVATR | | cytosolic small ribosomal subunit | | |
| 4 | | 40S ribosomal protein S9 | P46781 | RPS9 | | | 33.2 | | | 246 | | | KTYVTPR  SPYGGGRPGR  LFEGNALLR  KQVVNIPSFIVR  LDSQKHIDFSLR  LDQELKLIGEYGLR | | cytosolic small ribosomal subunit | | |
| 5 | | 60S ribosomal protein L18 | Q07020 | RPL18 | | | 28.3 | | | 193 | | | LAADDFR  IVLQIDNAR  QSVENDIHGLR  KVIDDTNITR  AQIFANTVDNAR  NLKASLENSLR  TNRPPLSLSR  TNSTFNQVVLKR  ENKTAVVVGTITDDVR | | cytosolic large ribosomal subunit | | |
| 6 | | 60S ribosomal protein L18a | Q02543 | RPL18A | | | 23.5 | | | 159 | | | EYKVVGR  IFAPNHVVAKSR  DLTTAGAVTQCYR | | cytosolic large ribosomal subunit | | |
| 7 | | 60S ribosomal protein L8 | P62917 | RPL8 | | | 14.0 | | | 109 | | | KGAGSVFR  KVGLIAAR  GAPLAKVVFR  IDKPILKAGR | | cytosolic large ribosomal subunit | | |
| **Nr.** | | **Protein name** | **Accession number** | **Gene name** | | | **Sequence coverage [%]** | | | **Mascot score** | | | **Peptide sequence** | | **Subcellular location** | | |
| 8 | | 60S ribosomal protein L9 | P32969 | RPL9 | | | 24.0 | | | 145 | | | TVIVKGPR  NFLGEKYIR  SVYAHFPINVVIQENGSLVEIR  IDKPILKAGR | | ribosome | | |
| 9 | | Glutamate dehydrogenase 1, mitochondrial | P00367 | GLUD1 | | | 6.8 | | | 132 | | | TPCKGGIR  GASIVEDKLVEDLR  IIKPCNHVLSLSFPIR | | mitochondrial matrix | | |
| 10 | | Heterogeneous nuclear ribonucleoprotein A1 | P09651 | HNRNPA1 | | | 15.9 | | | 261 | | | NQGGYGGSSSSSSYGSGR  SSGPYGGGGQYFAKPR  KLFIGGLSFETTDESLR | | cytoplasm | | |
| 11 | | Polypyrimidine tract-binding protein 1 | P26599 | PTBP1 | | | 13.8 | | | 240 | | | ITLSKHQNVQLPR  IAIPGLAGAGNSVLLVSNLNPER  VTPQSLFILFGVYGDVQR  EGQEDQGLTKDYGNSPLHR | | heterogeneous nuclear ribonucleoprotein complex | | |
| 12 | | Glucosidase 2 subunit alpha | Q14697 | GANAB | | | 16.8 | | | 486 | | | AFFAGSQR  GLLEFEHQR  SIRPGLSPYR  NLGLYVKTR  FRIDELEPR  LKVTEGGEPYR  YRVPDVLVADPPIAR  LSFQHDPETSVLVLR  VSQGSKDPAEGDGAQPEETPR  QYASLTGTQALPPLFSLGYHQSR  AHAHLDTGRR | | endoplasmic reticulum lumen | | |
| 13 | | Glucosidase 2 subunit beta | P14314 | PRKCSH | | | 9.1 | | | 123 | | | NKFEEAER  TVKEEAEKPER  LLCGKETMVTSTTEPSR | | endoplasmic reticulum lumen | | |
| 14 | | Endoplasmin | P14625 | HSP90B1 | | | 13.5 | | | 513 | | | LAKLLR  KEAESSPFVER  ELISNASDALDKIR  SGYLLPDTKAYGDR  DDEVDVDGTVEEDLGKSR  FQSSHHPTDITSLDQYVER  EEEAIQLDGLNASQIR  TDDEVVQREEEAIQLDGLNASQIR | | endoplasmic reticulum lumen | | |
| **Nr.** | | **Protein name** | **Accession number** | **Gene name** | | | **Sequence coverage [%]** | | | **Mascot score** | | | **Peptide sequence** | | **Subcellular location** | | |
| 15 | | Protein disulfide-isomerase | P07237 | P4HB | | | 45.8 | | | 275 | | | GYPTIKFFR  NGDTASPKEYTAGR  DAPEEEDHVLVLR  LAKVDATEESDLAQQYGVR | | endoplasmic reticulum lumen | | |
| 16 | | Protein disulfide-isomerase A4 | P13667 | PDIA4 | | | 3.7 | | | 116 | | | KGRPYDYNGPR  FDVSGYPTLKIFR  TQEEIVAKVR  ELSDFISYLQR | | endoplasmic reticulum lumen | | |
| **After treatment with mutant rTcdA, 24 h** | | | | | | | | | | | | | | | |  |  |
| **Nr.** | | **Protein name** | **Accession number** | **Gene name** | | **Sequence coverage [%]** | | | **Mascot score** | | | **Peptide sequence** | | | **Subcellular location** |  |  |
| 1 | | Filamin A | P21333 | FLNA | | 3.3 | | | 264 | | | DAPQDFHPDR  ALTQTGGPHVKAR  NDNDTFTVKYTPR  VANPSGNLTETYVQDR  SPYTVTVGQACNPSACR  EGPYSISVLYGDEEVPR  VSGQGLHEGHTFEPAEFIIDTR | | | actin cytoskeleton |  |  |
| **After treatment with rTcdA wt, 24 h** | | | | | | | | | | | | | | | | | |
| **Nr.** | | **Protein name** | **Accession number** | **Gene name** | | **Sequence coverage [%]** | | | **Mascot score** | | | **Peptide sequence** | | **Subcellular location** | | | |
| 1 | | Actin, cytoplasmic 1 | P60709 | ACTB | | 28.6 | | | 445 | | | GYSFTTTAER  AVFPSIVGRPR  DLTDYLMKILTER  VAPEEHPVLLTEAPLNPKANR  KDLYANTVLSGGTTMYPGIADR  TTGIVMDSGDGVTHTVPIYEGYALPHAILR | | actin cytoskeleton | | | |
| **Nr.** | | **Protein name** | **Accession number** | **Gene name** | | **Sequence coverage [%]** | | | **Mascot score** | | | **Peptide sequence** | | **Subcellular location** | | | |
| 2 | | Filamin A | P21333 | FLNA | | 3.3 | | | 264 | | | DAPQDFHPDR  ALTQTGGPHVKAR  NDNDTFTVKYTPR  VANPSGNLTETYVQDR  SPYTVTVGQACNPSACR  EGPYSISVLYGDEEVPR  VSGQGLHEGHTFEPAEFIIDTR | | actin cytoskeleton | | | |
| 3 | | Filamin B | O75369 | FLNB | | 9.3 | | | 782 | | | VKESITR  GLQPKGVR  DEPCLLKR  ENSTAKFIPR  VKVEPAVDTSR  IGNLQTDLSDGLR  TFEMSDFIVDTR  LPNNHIGISFIPR  LIALLEVLSQKR  GEAGVPAEFSIWTR  KVVPCLVTPVTGR  GAGGQGKLDVTILSPSR  LVSPGSANETSSILVESVTR  YTIKVLFASQEIPASPFR  VLQSFTVDSSKAGLAPLEVR  DAGEGLLAVQITDQEGKPKR | | actin cytoskeleton | | | |
| 4 | | Villin-1 | P09327 | VIL1 | | 5.1 | | | 195 | | | EVQGNESEAFR  GYFKQGLVIR  LQEENLVITPR  LFECSNKTGR | | cytoskeleton | | | |
| 5 | | Apolipoprotein E | P02649 | APOE | | 7.0 | | | 82 | | | LSKELQAAQAR  AKLEEQAQQIR | | secreted | | | |
| 6 | | Creatine kinase B-type | P12277 | CKB | | 16.5 | | | 351 | | | GFCLPPHCSR  PFSNSHNALKLR  GTGGVDTAAVGGVFDVSNADR  AIEKLAVEALSSLDGDLAGR | | cytoplasm | | | |
| 7 | | Glucosidase 2 subunit beta | P14314 | PRKCSH | | 7.3 | | | 105 | | | NKFEEAER  TVKEEAEKPER | | endoplasmic reticulum lumen | | | |
| **Nr.** | | **Protein name** | **Accession number** | **Gene name** | | **Sequence coverage [%]** | | | **Mascot score** | | | **Peptide sequence** | | **Subcellular location** | | | |
| 8 | | Peroxiredoxin-1 | Q06830 | PRDX1 | | 28.1 | | | 295 | | | SVDETLR  GLFIIDDKGILR  TIAQDYGVLKADEGISFR  QITVNDLPVGR | | cytoplasm | | | |
| 9 | | Peroxiredoxin-2 | P32119 | PRDX2 | | 29.4 | | | 337 | | | QITVNDLPVGR  GLFIIDGKGVLR  KEGGLGPLNIPLLADVTR  LSEDYGVLKTDEGIAYR | | cytoplasm | | | |
| 10 | | Peroxiredoxin-6 | P30041 | PRDX6 | | 23.8 | | | 302 | | | NFDEILR  DFTPVCTTELGR  VVISLQLTAEKR  PGGLLLGDVAPNFEANTTVGR | | cytoplasm | | | |
| 11 | | Annexin A3 | P12429 | ANXA3 | | 20.1 | | | 282 | | | NTPAFLAER  SEIDLLDIR  KALLTLADGR  ALKGIGTDEFTLNR  DYPDFSPSVDAEAIQKAIR | | phagocytic vesicle membrane | | | |
